# Cytotoxic Microspinosamides from the Marine Sponge *Geodia microspinosa* That Decrease Diffuse Pleural Mesothelioma Viability

**DOI:** 10.3390/md24090308

**Published:** 2026-09-04

**Authors:** Maria Orfanoudaki, Dongdong Wang, Lin Du, Vivek Singh, Ekaterina I. Goncharova, Nathanael Pruett, Chuong D. Hoang, Brice A. P. Wilson, Barry R. O’Keefe

**Affiliations:** 1 Molecular Targets Program, Center for Cancer Research, National Cancer Institute, Frederick, MD 21702-1201, USA; orfmaria@gmail.com (M.O.); dongdong.wang@nih.gov (D.W.); lin.du@nih.gov (L.D.); okeefeba@nih.gov (B.R.O.); 2 Thoracic Surgery Branch, National Cancer Institute, National Institutes of Health, Bethesda, MD 20892, USA; vivek.singh@nih.gov (V.S.); nathanael.pruett@nih.gov (N.P.); chuong.hoang@nih.gov (C.D.H.); 3 Advanced Biomedical Computational Science, Frederick National Laboratory for Cancer Research, Frederick, MD 21702-1201, USA; katya.goncharova@nih.gov; 4 Natural Products Branch, Division of Cancer Treatment and Diagnosis, National Cancer Institute, Frederick, MD 21702-1201, USA

**Keywords:** microspinosamide, depsipeptide, diffuse pleural mesothelioma, high-throughput screen, *Geodia microspinosa*

## Abstract

A fraction from an extract of the marine sponge *Geodia microspinosa* was identified as active in a high-throughput screen for molecules that impair the viability of diffuse pleural mesothelioma (DPM) cell lines. Bioassay-guided isolation led to the identification of microspinosamides B and D, and a new artifact, microspinosamide C, together with two previously reported analogues, microspinosamide and polydiscamide B, as the active principles. Their planar structures were solved by NMR and HRESIMS analyses, while advanced Marfey’s reaction, ROESY, and ECD were used for the establishment of their absolute configurations. All pure metabolites demonstrated low micromolar potency for the reduction in viability of the DPM cell lines.

## 1. Introduction

Marine sponges are one of the largest sources of new bioactive molecules, although an increasing number of studies suggest that microbial symbionts, e.g., marine-derived fungi and bacteria, may be the actual producers of “sponge” derived metabolites [1,2]. Macrolides and peptides are the most dominant chemical classes isolated from sponges, and many of them exhibit mammalian cell cytotoxicity in addition to less frequent reports of antibacterial, antifungal, and enzyme-inhibitory activity [2].

Cárdenas et al. have extensively examined the molecular taxonomy and phylogeny of the Geodiidae family, which includes several species from the genera *Caminella*, *Melophlus*, *Caminus*, *Geodia*, *Pachymatisma*, *Erylus*, *Penares*, and *Rhabdastrella* [3]. In their studies, the *Isops* and *Sidonops* genera have been transferred to the *Geodia* genus based on molecular analysis and reevaluation of their morphological characteristics. Including over 160 species, many of which are found in deep waters, the genus *Geodia* is a producer of a wide spectrum of bioactive compounds [4]. From this large group, there has been only one study examining the chemical profile of the species *G. microspinosa* collected from Indonesia [5], which was reported as *Sidonops microspinosa* in 2001 [6]. In that study, Rashid et al. reported the discovery of microspinosamide (**2**), a 13-amino-acid-residue cyclic depsipeptide which includes a rare *β*-hydroxy-*p*-bromophenylalanine residue (*β*-OH-*p*-BrPhe) and several uncommon amino acid residues [6]. The cyclic depsipeptide **2** demonstrated antiviral activity against HIV (EC_50_ = 119 nM), which overlapped with cellular cytotoxicity in a CEM-SS lymphoblastic cell line [6].

While cytotoxicity in the context of antiviral activity is undesirable, anticancer cytotoxicity could be a potentially useful application for these compounds. For example, the poor prognostic outcomes and lack of available targeted therapies make diffuse pleural mesothelioma (DPM) an ideal disease target for the discovery of new anticancer modalities [7]. Unique amongst the many solid tumour types, DPM generally arises decades after exposure to asbestos. This environmental exposure leads to the development of a highly malignant carcinoma in the pleural mesothelium of the chest cavity. Although there are three DPM subtypes (epithelioid, biphasic, and sarcomatoid) categorized morphologically, each of these generally has very poor survival outcomes [8,9]. In an effort to discover new natural products and develop new treatment strategies for DPM, the National Cancer Institute launched a high-throughput screening campaign of a natural product fraction library (∼34,000 fractionated natural product extracts) against two patient-derived mesothelioma cell lines, MB24 (sarcomatoid) and MB52 (epithelial). To test for potential DPM specificity, all leads were also counter-screened against a patient-derived cell line model of normal human pleura NP1 [10].

## 2. Results

**Figure 1:**
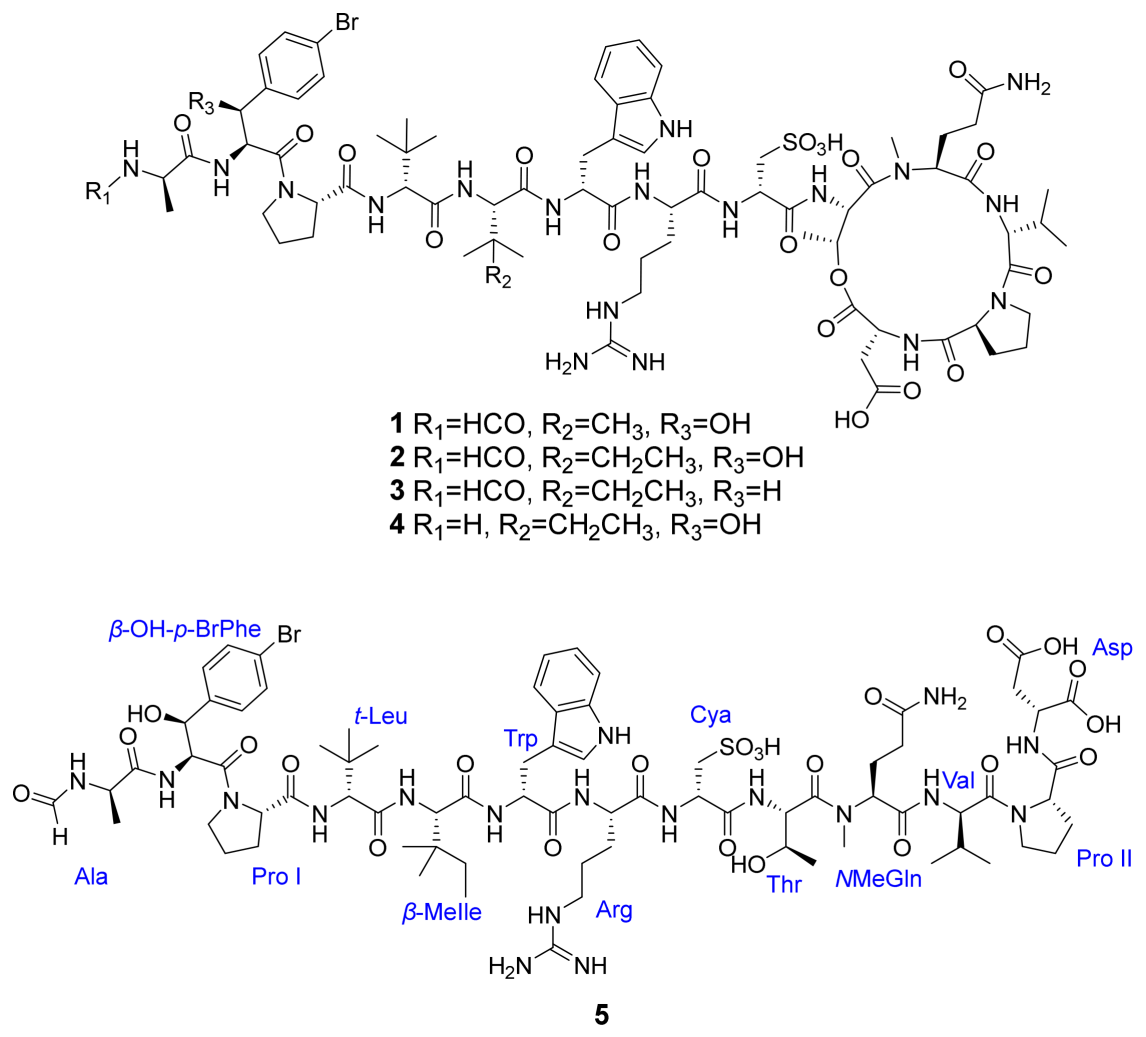
Chemical structures of microspinosamide B (**1**), microspinosamide (**2**), polydiscamide B (**3**), microspinosamide C (**4**), and microspinosamide D (**5**).

**Figure 2:**
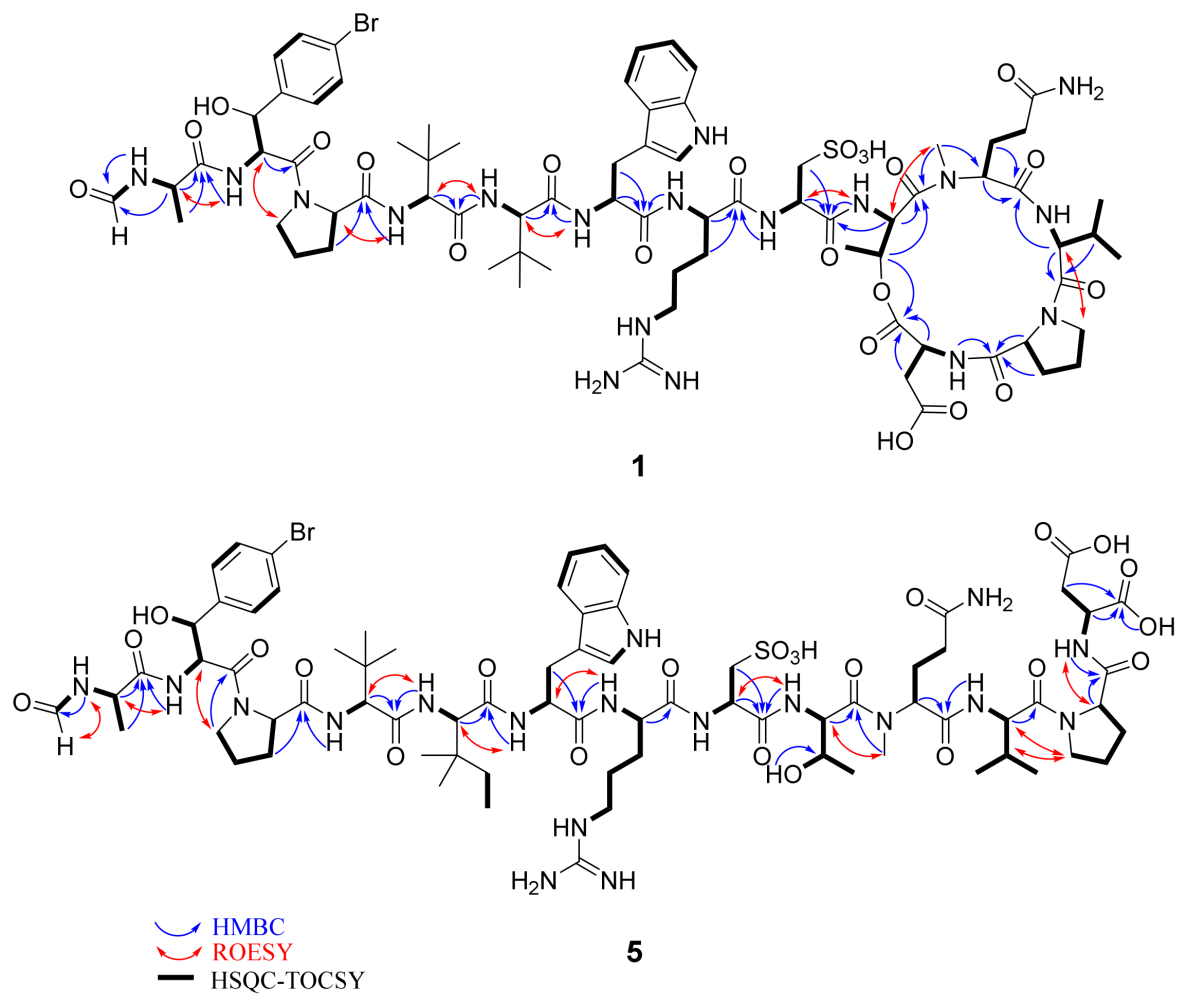
Key 2D NMR correlations for the establishment of structures of **1** and **5**.

**Figure 3:**
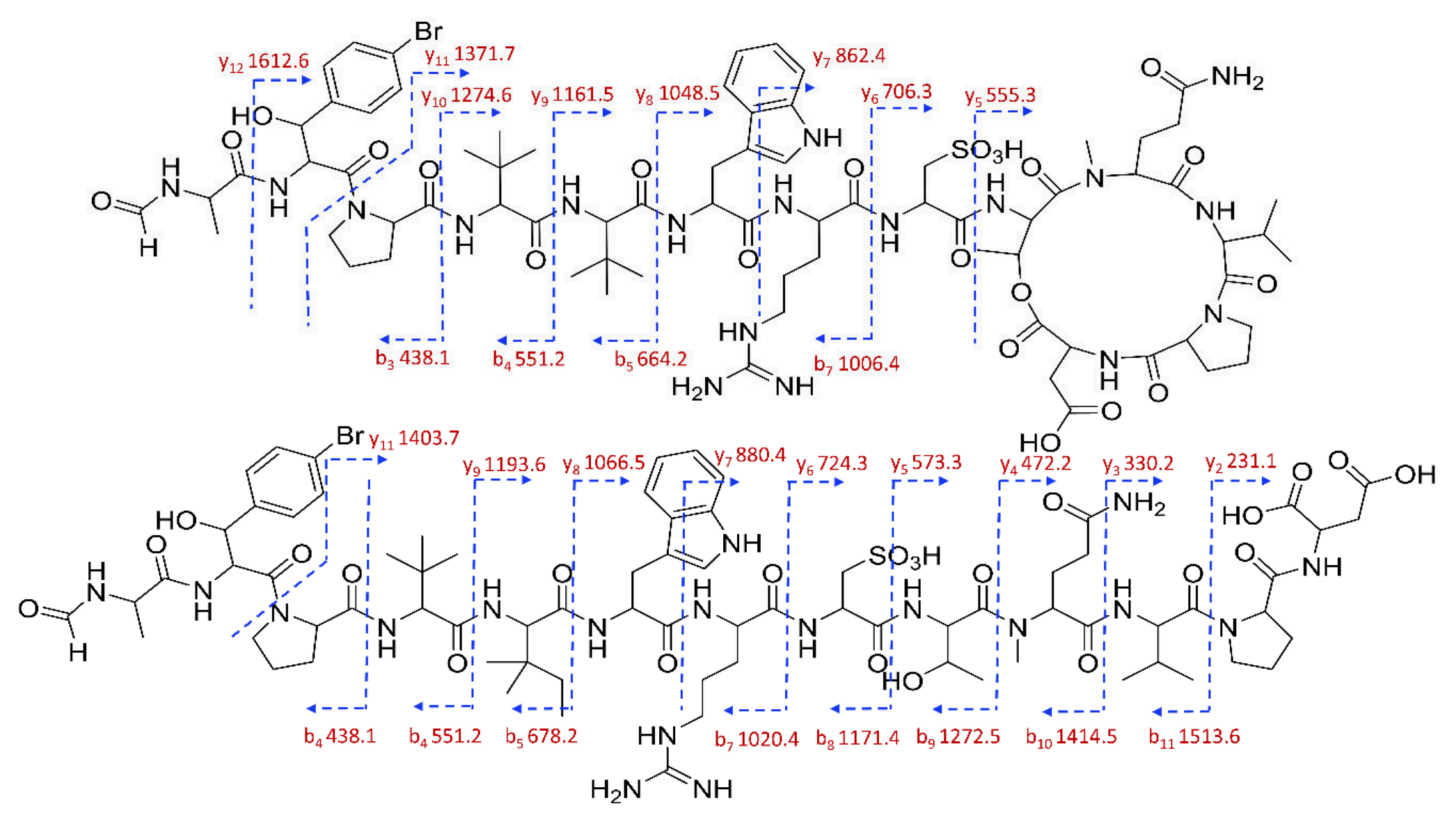
HR-ESI-MS^2^ fragmentation patterns of **1** and **5**.

Microspinosamide B (**1**) was isolated as a white amorphous solid. Two protonated molecular ion peaks [M+H]^+^ of the HRESIMS spectrum at *m*/*z* 1711.6703 (calculated for C_74_H_108_^79^BrN_18_O_22_S^+^, 1711.6784) and 1713.6743 (calculated for C_74_H_108_^81^BrN_18_O_22_S^+^, 1713.6786) in a ~1:1 ratio pattern confirmed the molecular formula. Analysis of the ^1^H and ^13^C NMR data revealed that **1** was a peptide, as shown by a series of amide groups and signals for *α*-amino resonances. Interpretation of the 2D NMR data revealed the amino acid residues to be one alanine (Ala), one aspartic acid (Asp), one tryptophan (Trp), one valine (Val), one arginine (Arg), two prolines (Pro I and Pro II), and one threonine (Thr), together with five unusual amino acid residues, including one *β*-hydroxy-*p*-bromophenylalanine (*β*-OH-*p*-BrPhe), one cysteic acid (Cya), one *N*-methylglutamine (*N*MeGln), and two *t*-leucines (*t*-Leu I and *t*-Leu II, Figure 1). Additionally, a carbon resonance at *δ*_C_ 162.0 with a corresponding proton resonance at *δ*_H_ 8.05 revealed the presence of a formamide moiety. Close examination of ^1^H and ^13^C NMR data (Table 1) identified **1** as a close analogue of the cyclic depsipeptide microspinosamide (**2**) [6]. The replacement of the *β*-methylisoleucine (*β*-MeIle) in **2** by a second *t*-leucine (*t*-Leu II) in **1** was evident due to the presence of three equivalent methyl groups (*γ*-CH_3_, *δ*_H_ 0.86 and *δ*_C_ 27.6) attached to the *β* carbon (*δ*_C_ 34.0) of the amino acid residue by ^1^H–^13^C HMBC correlations from *t*-Leu II-H*γ* to *t*-Leu II-C*β* (*δ*_C_ 34.0) and *t*-Leu II-C*α* (*δ*_C_ 63.5). The position of this amino acid residue was established by ROESY correlations between its *Ν*H (*δ*_H_ 8.19) and *t*-Leu I-H*α* (*δ*_H_ 4.70) and the Trp-*N*H (*δ*_H_ 8.45) and *t*-Leu II-H*α* (*δ*_H_ 4.18) and was supported by ^1^H–^13^C HMBC correlations as shown in Figure 2. HRESIMS fragment ions observed at the HR-ESI-MS^2^ mass spectrum further confirmed the planar structure of **1** (Figure 3).

**Figure 4:**
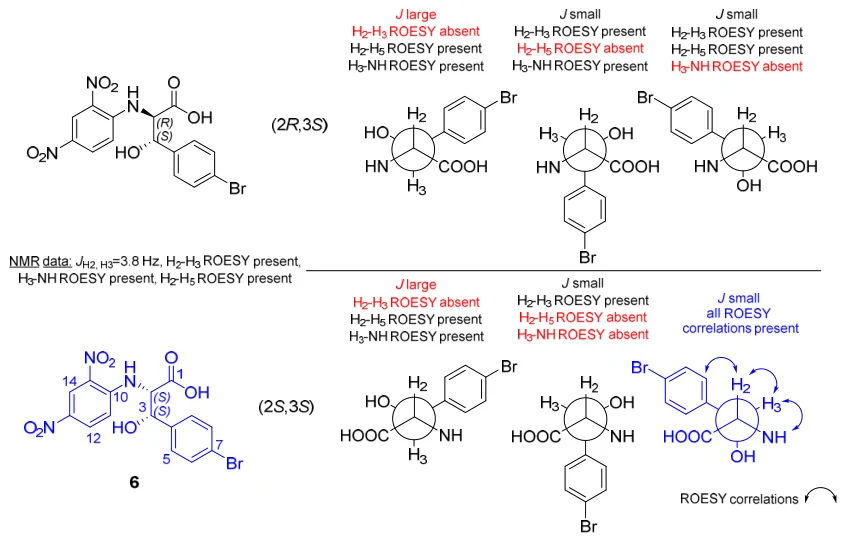
Newman projections showing all possible relative conformations, and ROESY correlations of the *β*-hydroxy-*p*-bromophenylalanine residue of **6** (opposing/missing NMR data marked in red, conformation in agreement with NMR data marked in blue).

**Figure 5:**
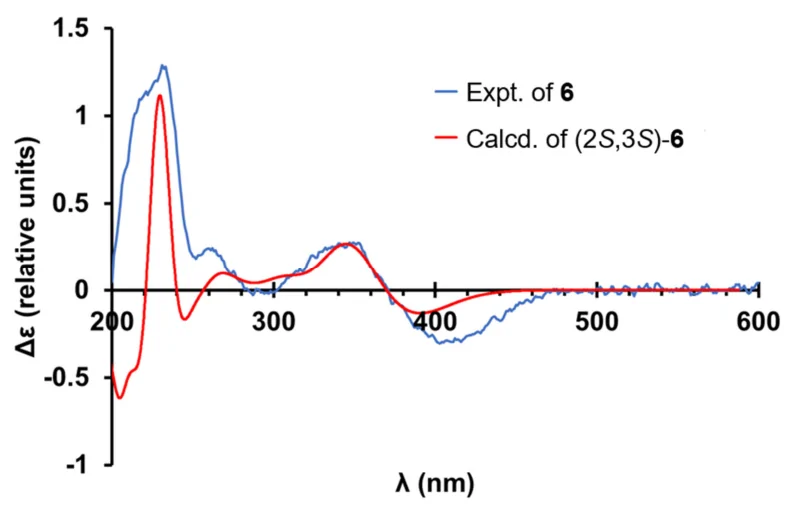
Experimental and calculated ECD spectra of **6** (blue and red, respectively).

The absolute stereochemistry of the amino acid residues of **1** was assigned by advanced Marfey’s reaction and LC-MS comparison with amino acid standards (Table S1). Tryptophan can be easily destroyed during conventional hydrolysis with 6 N HCl [11,12]; therefore, the ʟ- and ᴅ- standards of its by-product kynurenine [13,14] were used for its stereochemistry assignment (Table S1). Detection of ᴅ-kynurenine in the hydrolysate mixture of **1** revealed the presence of ᴅ-tryptophan in **1** (Table S1). Similarly, glutamine is unstable during hydrolysis with HCl, degrading into pyroglutamic acid and glutamic acid. ʟ-Glutamic acid was identified as the final reaction product of the advanced Marfey’s reaction (Table S1) in the hydrolysate mixture of **1**, indicating the ʟ-configuration of the glutamine in the peptide [15]. Both ᴅ-tert-leucine and ʟ-tert-leucine were detected in the hydrolysate of **1**. Comparison of the NMR chemical shifts of **1** with the co-occurring known peptides microspinosamide (**2**) and polydiscamide B (**3**) [16], as well as the structurally related discodermins [17,18], indicated the presence of ᴅ-*t*-leucine I and ʟ-*t*-leucine II in **1**. Therefore, the stereochemistry of the amino acid residues was defined as ᴅ-alanine, ʟ-threonine, ᴅ-valine, ᴅ-cysteic acid, ʟ-arginine, ʟ-*N*-methylglutamine, ʟ-proline, and ᴅ-aspartic acid in **1**. The relative configuration of the *β*-hydroxy-*p*-bromophenylalanine residue in **1** was determined to be (2S*,3S*) based on the high similarity of NMR chemical shifts and the retention time of the 1-fluoro-2,4-dinitrophenyl-5-ʟ-leucinamide (FDLA) derivative of this residue after hydrolysis of **2**. Compound **2** was hydrolyzed in 6 N HCl, and the hydrolysate was derivatized with 1-fluoro-2,4-dinitrobenzene (DNFB) to yield the 2,4-dinitrophenyl derivative of *β*-hydroxy-*p*-bromophenylalanine (**6**). Analysis of the coupling constants and ROESY correlations revealed the relative configuration of *β*-OH-*p*-BrPhe to be 2S*,3S* (Figure 4). The absolute configuration was assigned based on comparison of the computationally generated ECD spectrum of the 2*S*,3*S* isomer of **6** with experimental ECD data. The calculated ECD spectrum for (2*S*,3*S*)-**6** was simulated using time-dependent density functional theory (TDDFT) at the CAM-B3LYP/DGDZVP level in MeOH. The calculated ECD spectrum showed high similarity with the experimental one (positive Cotton effects at 234, 261, 346 nm, and a negative one at 410 nm), determining the absolute configuration of *β*-hydroxy-*p*-bromophenylalanine as 2*S*,3*S* (Figure 5). This assignment is in agreement with the structures of other related peptides of this family which are ʟ-configured (2*S*-configured) at the *β*-OH-*p*-BrPhe residue [16,19] and with the findings of a study preparing a synthetic microspinosamide analogue incorporating a (2*S*,3*R*)-*β*-hydroxy-*p*-bromophenylalanine residue that showed differences in the retention time and NMR data compared to the natural product, revealing that a non-natural diastereomer of the natural metabolite had been synthesized [20].

The molecular formula C_74_H_109_BrN_18_O_21_S was assigned to microspinosamide C (**4**) based on the ~1:1 ratio ions at *m*/*z* [M+H]^+^ 1697.7055 (calculated for C_74_H_110_^79^BrN_18_O_21_S^+^, 1697.6992) and [M+H]^+^ 1699.6985 (calculated for C_74_H_110_^81^BrN_18_O_21_S^+^, 1699.6994) observed in the (+)-HRESIMS spectrum. ^1^H and ^13^C NMR data of **4** had high similarity to those of **2** [6], with the only difference at the *N*-terminus being that the formamide moiety in **2** was replaced by a terminal amine (*δ*_H_ 8.43) in **4**. ROESY correlations of Ala-*N*H_2_/Ala-H*α* confirmed the placement of the free *N*H_2_ group, completing the structure determination of **4**. Not detected in the bioactive-enriched fraction, **4** was isolated as a minor compound during the following HPLC purification procedure for the isolation of the major compound **2** with the presence of 0.1% trifluoracetic acid (TFA) while the yield of **2** dropped (Figures S1 and S2); therefore, **4** was considered a degradation artifact of **2**.

Microspinosamide D (**5**) was isolated as a white amorphous solid. Its molecular formula was deduced to be C_75_H_111_BrN_18_O_23_S by HRESIMS measurements and NMR data (Table 2). Analysis of 2D NMR spectra of **5** identified 13 amino acid residues, identical to those of **2**. The mass difference of +18 Da indicated that **5** was the ring-opened derivative of the cyclic depsipeptide **2** (Figure 1), which was supported by differences in the chemical shifts in the threonine and aspartic acid residues and HMBC correlations from the two OH protons to the carbons of threonine and aspartic acid residues (Asp-OH at *δ*_H_ 4.90 to Asp-C*α* at *δ*_C_ 50.0 and Asp-CO at *δ*_C_ 173.1 and from Thr-OH at *δ*_H_ 4.91 to Thr-C*β* at *δ*_C_ 68.6 and Asp-C*γ* at *δ*_C_ 19.9, Figure 2). The structure of the linear peptide **5** was further supported by the fragment ions observed in the HR-ESI-MS^2^ mass spectrum (Figure 3). The absolute configurations of the individual amino acid constituents of compound **5** were determined by advanced Marfey’s method [21] and by comparing the retention time of the hydrolyzed compound **5** with those of amino acid standards, which revealed the presence of ʟ-*β*-methylisoleucine, ᴅ-tert-leucine, and amino acid residues with identical stereochemistry as in **1**.

Additionally, careful analysis of the ^13^C NMR data and the pattern of dipolar coupling in the ROESY spectrum revealed the configuration of the peptide bond preceding the proline units [22,23]. In all cases, the *trans* geometries of the two proline amide bonds were determined by ROESY correlations between the *δ*-protons of proline residues and the *α*-protons of the preceding amino acid residues, as well as the small carbon chemical shift differences between C*β* and C*γ,* which were smaller than 5 ppm (Δ*δ*_C_*_β_*_-C_*_γ_* < 5 ppm) [22,23].

The structures of the two known cyclic depsipeptides, microspinosamide (**2**) and polydiscamide B (**3**), were identified after comparison of their spectroscopic and spectrometric data (NMR and HRESIMS) with those reported in the literature [6,16].

**Table 2:** NMR spectroscopic data for microspinosamide D (**5**) in DMF-*d*_7_ (600 MHz for ^1^H, 150 MHz for ^13^C, and 60 MHz for ^15^N, *δ* in ppm).

Amino Acid	Position	*δ*_C_*/δ*_N_ ^a^, Type	*δ*_H_, Mult (*J* in Hz)	HMBC	ROESY
ᴅ-alanine (Ala)	HCO	162.3, C	8.12, ^b^	48.1	8.27
	*N*H	128.9, *N*H	8.27, d (7.2)	18.8, 48.1, 162.3	4.41, 8.12
	CO	172.5, C	-	-	-
	*α*	48.1, CH	4.41, m	18.8, 162.3, 172.5	8.20, 8.27
	*β*	18.8, CH_3_	0.99, ^b^	48.1	-
*β*-hydroxy-*p-*bromophenylalanine (*β*-OH-*p*-BrPhe)	*N*H	112.8, *N*H	8.20, d (9.8)	172.5	4.41, 4.77
	CO	172.2, C	-	-	-
	*α*	57.5, CH	4.85, m	75.6, 142.6	3.79, 4.03, 5.88, 7.53
	*β*	75.6, CH	4.77, m	57.5, 130.6, 142.6	5.88, 7.53
	C-1	142.6, C	-	-	-
	C-2/C-6	130.8, CH	7.52, d (8.4)	75.6, 122.0, 130.8	4.77, 4.85
	C-3/C-5	132.0, CH	7.58, d (8.4)	131.9, 142.6	-
	C-4	122.0, C	-	-	-
	OH	-	5.88, d (15.8)	57.5, 75.6, 142.6	4.77, 4.85
ʟ-proline I (Pro I)	CO	172.7, C	-	-	-
	*α*	61.3, CH	4.84, m	25.4, 30.3	1.98, 3.79, 4.03
	*β*	30.3, CH_2_	2.24, m 1.96, m	25.4, 48.4, 61.3, 172.7	-
	*γ*	25.4, CH_2_	1.98, m	30.3, 48.4, 61.3	4.84
	*δ*	48.8, CH_2_	4.03, m 3.79, m	25.4, 30.3, 61.3, 171.9	4.84
ᴅ-*t*-leucine (*t*-Leu)	*N*H	^b^	8.11, ^b^	60.9, 172.7	4.72
	CO	172.8, C	-	-	-
	*α*	60.9, CH	4.72, br s	36.4, 172.8	0.99, 8.11, 8.13
	*β*	36.4, C	-	-	-
	*γ*	27.7, CH_3_	0.99, s	27.7, 36.4, 60.9	4.72
ʟ-*β*-methylisoleucine (*β*-MeIle)	*N*H	^b^	8.13, ^b^	62.2, 172.8	4.72
	CO	172.2, C	-	-	-
	*α*	62.2, CH	4.08, ^b^	23.7, 23.8, 32.9, 36.2, 172.2	0.72, 8.45
	*β*	36.2, C	-	-	-
	*γ*	32.9, CH_2_	1.25, m 1.18, m	8.7, 23.7, 23.8, 62.2	0.72
	*δ*	8.7, CH_3_	0.72, m	36.2	1.18, 4.08
	*γ*′	23.7, CH_3_	0.73, s	23.8, 32.9, 62.2	0.84
	*γ*″	23.8, CH_3_	0.84, s	23.7, 32.9, 36.2, 62.2	0.73
ᴅ-tryptophan (Trp)	*N*H-indole	129.3, *N*H	10.86, br s	111.5, 125.2, 128.7, 137.9	7.25, 7.39
	*N*H	120.2, *N*H	8.45, d (8.2)	54.5, 172.2	4.08
	CO	173.6, C	-	-	-
	*α*	54.5, CH	4.80, m	28.4, 111.5	3.39
	*β*	28.4, CH_2_	3.39, m 3.07, m	111.5, 125.2, 128.7, 173.6	4.80
	C-2	125.2, CH	7.25, br s	28.4, 111.5, 128.7, 137.9	10.86
	C-3	111.5, C	-	-	-
	C-3*α*	128.7, C	-	-	-
	C-4	119.5, CH	7.57, d (7.8)	111.5, 122.0, 137.9	6.99
	C-5	119.5, CH	6.99, t (7.8)	112.4, 128.7	7.57
	C-6	122.0, CH	7.07, t (7.8)	119.5, 137.9	7.39
	C-7	112.4, CH	7.39, d (7.8)	119.5, 128.7	7.07
	C-7*α*	137.9, C	-	-	-
ʟ-arginine (Arg)	*N*H-guanidine	85.4, *N*H	7.83, br s	25.2, 42.0, 158.7	3.18
	C-guanidine	158.7, C	-	-	-
	*N*H	112.4, *N*H	8.37, br s	173.6	4.80
	CO	173.3, C	-	-	-
	*α*	55.5, CH	4.30, m	29.3, 173.3	-
	*β*	29.3, CH_2_	1.92, ^b^	25.2, 42.0, 55.5	-
	*γ*	25.2, CH_2_	1.66, m	29.3, 42.0, 55.5	-
	*δ*	42.0, CH_2_	3.18, m	25.2, 29.3, 158.7	7.83
ᴅ-cysteic acid (Cya)	*N*H	110.6, *N*H	8.34, br s	-	-
	CO	171.6, C	-	-	-
	*α*	53.0, CH	4.89, m	52.0, 171.6	3.29, 7.94
	*β*	52.0, CH_2_	3.29, m	53.0, 171.6	4.89
ʟ-threonine (Thr)	*N*H	110.6, *N*H	7.94, d (8.2)	55.3, 171.6	4.89
	CO	173.4, C	-	-	-
	*α*	55.3, CH	5.03, m	68.6, 173.4	1.18, 3.06, 4.32
	*β*	68.6, CH	4.32, m	19.9	1.18, 4.32
	*γ*	19.9, CH_3_	1.18, d (6.2)	53.5, 72.1	4.32, 5.03
	OH	-	4.91, br s	19.9, 68.6	3.06
ʟ-*N*-methylglutamine (*N*MeGln)	*N*Me	32.0, CH_3_	3.06, s	55.3, 68.6	5.03
	CO	171.9, C	-	-	-
	O=C-*N*H_2_	106.5, *N*H	7.49, ^b^	175.6	-
	O=C-*N*H_2_	175.6, C	-	-	-
	*α*	57.5, CH	5.27, br s	32.0, 171.9	2.20
	*β*	25.0, CH_2_	2.11, m	57.5	-
	*γ*	32.5, CH_2_	2.27, m 2.20, m	25.0, 57.5, 175.6	5.27
ᴅ-valine (Val)	*N*H	116.7, *N*H	7.71, br d (8.0)	30.9, 57.9, 171.9	-
	CO	171.2, C	-	-	-
	*α*	57.9, CH	4.36, br d (8.0)	19.2, 30.9, 171.2	0.90, 2.05, 3.66, 3.88
	*β*	30.9, CH	2.05, m	19.2, 57.9	0.90, 3.66, 3.88, 4.36
	*γ*′	20.3, CH_3_	0.90, d (6.4)	18.5, 31.0, 56.8	2.06, 4.55
	*γ*	18.5, CH_3_	0.90, d (6.4)	20.3, 31.0, 56.8	2.06, 4.55
ʟ-proline II (Pro II)	CO	172.4, C	-	-	-
	*α*	61.4, CH	4.48, br d (7.6)	25.6, 30.5, 48.3, 172.4	7.88
	*β*	30.5, CH_2_	2.12 m 1.96, m	25.6, 61.4	-
	*γ*	25.6, CH_2_	1.98, m	30.5, 48.7, 61.4	-
	*δ*	48.3, CH_2_	3.88, m 3.66, m	25.6, 30.5, 61.4	2.05, 4.36
ᴅ-aspartic acid (Asp)	*N*H	113.6, *N*H	7.88, d (8.0)	50.0, 172.4	4.45
	CO	173.1, C	-	-	-
	COOH	173.5, C	-	-	-
	*α*	50.0, CH	4.80, m	37.3, 173.1, 173.5	2.74, 2.84
	*β*	37.3, CH_2_	2.84, m 2.74, m	50.0, 173.1, 173.5	4.80
	OH	-	4.90, br s	50.0, 173.1	-

^a^ Assignments were extracted from ^1^H–^15^N HSQC and HMBC spectra. ^b^ Signal overlap prevents the determination of coupling constants or chemical shifts.

Following completion of bioassay-guided fractionation, compounds **1**–**5** were isolated and tested in the DPM assay [10] with the results shown in Table 3. The primary screening assay involved only the two DPM cell lines: MB24, representative of the sarcomatoid subtype, and MB52, representative of the epithelioid subtype. To further determine any potential cancer specificity, the bioassay-guided fractionation included a counter-screen against a cell line model of the normal pleura, NP1. Potency measures against all three cell lines are shown in Table 3 for all compounds as well as a positive control for general cytotoxicity, sanguinarine chloride. Complete dose–response curves for compounds **1**–**5** are included in the Supporting Information (Figures S3–S7).

## 3. Discussion

Our study reports on the chemical investigation of the sponge *G. microspinosa* following its identification as active in a high-throughput screen for natural products that reduce the viability of DPM cell lines. To date, there has been only one secondary metabolite reported from *G. microspinosa*, microspinosamide (**2**), a cyclic depsipeptide that exhibited antiviral properties with undesirable cell line cytotoxicity [6], which was among the bioactive analogues identified in our screen.

Among the five compounds identified from this sponge, the two known cyclic depsipeptides **2** and **3** were the most active with LD_50_ values below 1 μM. The new analogue **1** was also active with a similar LD_50_ value. These results suggested that the absence of a *β*-hydroxy group on the *p*-bromophenylalanine moiety (compound **3**) and/or the substitution of a *β*-methyl-isoleucine with tert-leucine (compound **1**) had limited effects on the observed biological activity. Compounds **4** and **5** showed lower potency, with LD_50_ values of ~8.0 and 1.5 μM, respectively, suggesting that a free *N*-terminus or ring opening has deleterious effects on potency and that these structural modifications on the microspinosamide skeletons should be avoided in order to retain cytotoxicity. All of the compounds demonstrated overlapping cytotoxicity with the normal pleura cell line model NP1, indicating a mechanism of cytotoxicity that did not significantly differentiate between normal pleura and DPM.

Linear peptides are biosynthetic precursors of cyclic depsipeptides [24,25,26], and other studies have also reported the reduction or loss of biological activities of linear analogues of the cyclic depsipeptides, suggesting that cyclic and three-dimensional structural features of these peptides are essential for biological activities [27,28]. Similarly, lower levels of activity have been reported from peptides with an identical amino acid sequence but without the *N*-formyl group in comparison to their *N*-formyl analogues [29], further highlighting the importance of the structure and chemical composition of these bioactive natural products.

## 4. Materials and Methods

### 4.1. General Experimental Procedures

Optical rotations were measured on a Rudolph Research Analytical AUTOPOL IV automatic polarimeter (Rudolph Research Analytical, Hackettstown, NJ, USA) using a cell of 0.25 dm path length and MeOH as the solvent at 25 °C. UV spectra were obtained with a Varian Cary 50 Bio spectrophotometer (Agilent, Santa Clara, CA, USA). IR spectra were recorded with a Bruker ALPHA II FT-IR spectrometer (Bruker, Billerica, MA, USA). NMR spectra were acquired with a Bruker Avance III NMR spectrometer (Bruker, Billerica, MA, USA) equipped with a 3 mm CP TCI probe operating at a frequency of 600 MHz for the ^1^H nucleus, 150 MHz for the ^13^C nucleus, and 60 MHz for the ^15^N nucleus with nonuniform sampling (NUS) set to 25% for ^1^H–^1^H, ^1^H–^13^C and ^1^H–^15^N detection 2D experiments, except for ROESY and HSQC-TOCSY experiments with NUS set to 40% using in all cases the standard Bruker pulse sequences. Spectra were calibrated to residual solvent signals at *δ*_H_ 2.50 and *δ*_C_ 39.5 for DMSO-*d*_6_, and *δ*_H_ 8.03 and *δ*_C_ 163.2 for DMF-*d*_7_. The *δ*_N_ values were not calibrated to an external standard but were referenced to neat NH_3_ (*δ* 0.00) using the standard Bruker parameters. HRESIMS data (MS1 data) were acquired on an Agilent 6530B accurate-mass Q-TOF Dual-ESI LC/MS (1260 Infinity) instrument, while HR-ESI-MS^2^ data were acquired on an Agilent 1260 Infinity II UHPLC system (Agilent Technologies, Santa Clara, CA, USA) coupled to an Agilent 6545 Q-TOF equipped with a dual AJS ESI source. HPLC separations were performed on a Gilson HPLC system (Middleton, WI, USA) equipped with a 322 pump, a 172-diode array detector, and a GX-281 liquid handler. HPLC fractions were dried on an SP Genevac system (Warminster, PA, USA). Widepore C4 silica (40 μm, 275 Å) material was purchased from JT Baker. All chemicals for Marfey’s reaction were purchased from Sigma-Aldrich (St. Louis, MO, USA), Ambeed (Buffalo Grove, IL, USA), Acrotein ChemBio Inc. (Hoover, Alabama, USA), and TCI Chemicals (Tokyo, Japan). The isolated compounds were dissolved in deuterated MeOD-*d_4_*, DMSO-*d_6_* or DMF-*d_7_* from Cambridge Isotope Laboratories (Tewksbury, MA, USA). All solvents required for isolation and analytical experiments were purchased from Sigma-Aldrich (St. Louis, MO, USA), Fisher Scientific (Waltham, MA, USA), and Honeywell (Charlotte, NC, USA). Ultrapure water was produced by a Hydro purification system (Hydro, Durham, NC, USA).

### 4.2. Collection, Extraction, and Isolation

The sponge *G. microspinosa* was collected (60 m depth) in Indonesia in May 1993 and was taxonomically identified by Dr. Michelle Kelly-Borges. A voucher specimen (#0CDN1395) for the collection was deposited at the Smithsonian Institution (Suitland, MD). The sponge (wet weight 145.7 g) was extracted in water, followed by a MeOH/DCM (1:1) overnight soak according to the National Cancer Institute’s standard marine extraction procedure [30] to give the aqueous extract C11262 (42.9 g) and the organic solvent extract C11263 (4.4 g). A portion of C11262 (5 g) was solubilized in 50 mL of distilled H_2_O, vortexed, sonicated, and tumbled for 2.5 h at 4 °C. Afterwards, 50 mL of 100% EtOH (4 °C) was added, the mixture was vortexed and sonicated, followed by stationary storage at 4 °C overnight. The next day, the sample was centrifuged at 10,000× *g* for 15 min at 10 °C. The sample was dried under N_2_ overnight.

Subsequently, the dried ethanol supernatant was reconstituted in water and prefractionated on Widepore C_4_ material (column dimensions: 160 × 70 cm) eluting with: water 100% to yield 3.3 g of fraction 1, water/MeOH (9:1) to yield 111.0 mg of fraction 2, water/MeOH (2:3) to yield 179.0 mg of fraction 3, and MeOH/ACN (1:1) to yield 15.2 mg of fraction 4. Bioactivity data showed that the bioactive metabolites were present in fraction 3, which was further purified on preparative HPLC using a Luna C_18_ column (150 × 21.2 mm) at a flow rate of 10 mL/min, eluting with a gradient from water/acetonitrile (98:2, 0.1% TFA) to water/acetonitrile (20:80, 0.1% TFA) over 40 min. Fractions were collected in 30 s increments. One of its fractions was subjected to preparative HPLC using the same column and flow rate with a gradient from water/acetonitrile (80:20, 0.1% TFA) to water/acetonitrile (35:65, 0.1% TFA) over 40 min, and subsequently to a new fractionation with the same column and flow rate with a gradient from water/acetonitrile (80:20, 0.1% TFA) to water/acetonitrile (45:55, 0.1% TFA) over 40 min, to give compound **2** (20.3 mg, 0.406% crude aqueous extract weight), compound **4** (4.2 mg, 0.084% crude aqueous extract weight) and a subfraction which was further purified on the same column and flow rate with a gradient from water/acetonitrile (80:20, 0.1% TFA) to water/acetonitrile (55:45, 0.1% TFA) over 40 min to give compound **3** (1.5 mg, 0.030% crude aqueous extract weight), and compound **5** (1.3 mg, 0.026% crude aqueous extract weight). Another fraction from the initial preparative HPLC was further purified on the same column and flow rate, eluting with a gradient from water/acetonitrile (80:20, 0.1% TFA) to water/acetonitrile (60:40, 0.1% TFA) over 90 min to yield compound **1** (1.1 mg, 0.022% crude aqueous extract weight).

**Microspinosamide B (1)**: white amorphous powder; ${\left[\alpha \right]}_{D}^{20}$ = −33.5, [*α*]_546_ = −33.5, [*α*]_633_ = −26.5 (*c* 0.086, MeOH); UV (MeOH) λ_max_ (log *ε*) 222 (5.00), 278 (4.08), nm; IR (film) 3297, 2942, 2832, 1638, 1522, 1445, 1382, 1187, 1024 cm^−1^; ^1^H, ^13^C and ^15^N NMR data in DMF-*d*_7_, see Table 1; (+)-HRESIMS *m*/*z* [M+H]^+^ 1711.6703 (calculated for C_74_H_108_^79^BrN_18_O_22_S^+^, 1711.6784) and [M+H]^+^ 1713.6743 (calculated for C_74_H_108_^81^BrN_18_O_22_S^+^, 1713.6786).

**Microspinosamide (2)**: white amorphous powder; ${\left[\alpha \right]}_{D}^{20}$ = +6.3, [*α*]_546_ = +8.8, [*α*]_633_ = +5.0 (*c* 0.32, MeOH); NMR data in agreement with previous studies [6]; (+)-HRESIMS *m*/*z* [M+H]^+^ 1725.6900 (calculated for C_75_H_110_^79^BrN_18_O_22_S^+^, 1725.6941) and [M+H]^+^ 1727.6916 (calculated for C_75_H_110_^81^BrN_18_O_22_S^+^, 1727.6943).

**Polydiscamide B (3)**: white amorphous powder; ${\left[\alpha \right]}_{D}^{20}$ = −8.4, [*α*]_546_ = −5. 9, [*α*]_633_ = −7.4 (*c* 0.13, MeOH); NMR data in agreement with previous studies [16]; (+)-HRESIMS *m*/*z* [M+H]^+^ 1709.6997 (calculated for C_75_H_110_^79^BrN_18_O_21_S^+^, 1709.6992) and [M+H]^+^ 1711.7005 (calculated for C_75_H_110_^81^BrN_18_O_21_S ^+^, 1711.6994).

**Microspinosamide C (4)**: white amorphous powder; ${\left[\alpha \right]}_{D}^{20}$ = −0.3, [*α*]_546_ = −14.1, [*α*]_633_ = −13.7 (*c* 0.12, MeOH); UV (MeOH) λ_max_ (log *ε*) 220 (4.90), 272 (3.93) nm; IR (film) 3310, 2966, 1643, 1601, 1533, 1449, 1200 cm^−1^; ^1^H, ^13^C and ^15^N NMR data in DMF-*d*_7_, see Table S1; (+)-HRESIMS *m*/*z* [M+H]^+^ 1697.7055 (calculated for C_74_H_110_^79^BrN_18_O_21_S^+^, 1697.6992) and [M+H]^+^ 1699.6985 (calculated for C_74_H_110_^81^BrN_18_O_21_S^+^, 1699.6994).

**Microspinosamide D (5)**: white amorphous powder; ${\left[\alpha \right]}_{D}^{20}$ = −28.1, [*α*]_546_ = −25.3, [*α*]_633_ = −28.1 (*c* 0.057, MeOH); UV (MeOH) λ_max_ (log *ε*) 223 (4.86), 276 (3.94) nm; IR (film) 3310, 2964, 2930,1638, 1529, 1449, 1402, 1231, 1198, 1041 cm^−1^; ^1^H, ^13^C and ^15^N NMR data in DMF-*d*_7_, see Table 2; (+)-HRESIMS *m*/*z* [M+H]^+^ 1743.7031 (calculated for C_75_H_112_^79^BrN_18_O_23_S^+^, 1743.7046) and [M+H]^+^ 1745.7091 (calculated for C_75_H_112_^81^BrN_18_O_23_S^+^, 1745.7049).

**Preparation of (2*****R*****,3*****R*****)-3-(4-bromophenyl)-2-((2,4-dinitrophenyl)amino)-3-hydroxypropanoic acid (6)**: compound **2** (13 mg) was heated at 100 °C for 24 h in 6 N HCl (4.3 mL). The solvent in the hydrolysate was evaporated under N_2_ and treated with 1-fluoro-2,4-dinitrobenzene (DNFB, 130 μL) and NaHCO_3_ (130 mg) in 50% EtOH (3.5 mL). The reactants were stirred at 70 °C for 1 h before being neutralized with 6 N HCl (250 μL), and the solvent was removed under nitrogen, compound **6** was isolated using a Luna C_18_ column (150 × 21.2 mm) at 10 mL/min eluting with a gradient from water/acetonitrile (80:20, 0.1% TFA) to water/acetonitrile (50:50, 0.1% TFA) over 80 min followed by Sephadex LH-20 (20 cm × 1.0 cm) with MeOH [0.1% TFA], yellow amorphous powder; UV (MeOH) λ_max_ (log ε) 224 (3.54), 267 (3.16), 349 (3.40) nm; ECD (235 μM, MeOH) λ (Δε) 234 (+1.2), 261 (+0.2), 296 (0), 346 (+0.3), 373 (0), 410 (−0.3), 472 (0) nm; IR (film) 3332, 2919, 1691, 1618, 1589, 1523, 1426, 1337, 1205, 1139, 1072, 1010 cm^−1^; ^1^H NMR (DMSO-*d*_6_, 600 MHz) *δ* 8.89 (1H, d, *J* = 8.0 Hz, 2-*N*H), 8.88 (1H, d, *J* = 2.8 Hz, H-14), 8.28 (1H, dd, *J* = 9.6, 2.8 Hz, H-12), 7.54 (2H, d, *J* = 8.2 Hz, H-6 and H-8), 7.37 (1H, d, *J* = 9.6 Hz, H-11), 7.33 (2H, d, *J* = 8.2 Hz, H-5 and H-9), 6.29 (1H, br s, 3-OH), 5.22 (1H, d, *J* = 3.8 Hz, H-3), 5.03 (1H, dd, *J* = 8.0, 3.8 Hz, H-2); ^13^C NMR (CDCl_3_, 151 MHz) *δ* = 61.3 (C-2), 72.3 (C-3), 116.6 (C-11), 120.8 (C-7), 123.5 (C-14), 128.7 (C-5 and C-9), 129.9 (C-12), 130.1 (C-15), 130.8 (C-6 and C-8), 135.6 (C-13), 139.5 (C-4), 147.6 (C-10), 169.7 (C-1); (-)-HRESIMS *m*/*z* [M-H]^−^ 423.9778 (calculated for C_15_H_11_^79^BrN_3_O_7_^−^, 423.9786) and [M-H]^−^ 425.9756 (calculated for C_15_H_11_^81^BrN_3_O_7_^−^, 425.9767).

### 4.3. Determination of the Stereochemistry of Amino Acid Residues

Approximately 0.1 mg of each of compounds **1**–**5** was stirred with 6 N HCl (600 μL) at 110 °C for 17 h according to a previously published protocol [31]. The hydrolysate was dried and resuspended in H_2_O (100 μL). Standard amino acids (200 μg) and the hydrolysate (ca. 100 µg) were dissolved in saturated NaHCO_3_ (40 μL), and 1% ᴅ- or ʟ-FDLA in acetone (100 μL) was added. The reaction vial was incubated and stirred for 50 min at 40 °C. The reaction was then quenched with 1 N HCl (20 μL). After the addition of acetonitrile (810 μL), the reaction products were analyzed by LC-MS, using a Kinetex 5 µm C_18_ column (50 × 2.1 mm, Phenomenex, Torrance, CA, USA). Water with 0.1% FA (A) and acetonitrile with 0.1% FA (B) were used as solvents applying the gradient: 0 min: 5% B, 5 min: 5% B, 45 min: 35% B, 46 min: 100% B, 51 min: 100% B, 52–55 min: 5% B for all the other amino acids. The diode array detector was set to wavelengths of 210, 254, 280, and 340 nm; the flow rate, injection volume, and column temperature were adjusted to 1 mL/min, 10 μL, and 40 °C, respectively. MS spectra were recorded in positive-ESI mode (capillary voltage 3 kV), with a drying gas temperature of 350 °C, the nebulizer gas (nitrogen) set to 50 psi, and a nebulizer flow (nitrogen) of 12.0 L/min.

### 4.4. Cell-Based Discovery and Characterization Campaign

Active extracts from *G. microspinosa* were identified in the context of a high-throughput screening campaign previously described [10]. Briefly, fractionated extracts from the NCI Programfor Natural Products Discovery screening library were assessed for cytotoxicity against the DPM cell lines, MB24 and MB52 (MesoBank, Cambridge, UK) in an XTT cell viability assay [32]. Following active fraction identification, bioassay-guided fractionation was undertaken against both mesothelioma cell lines as well as the normal pleural cell line model (NP1) developed by Pruett et al. to evaluate cancer versus normal cytotoxicity [33]. A positive control for general cytotoxicity, sanguinarine chloride (AK Scientific, Inc., Union City, CA, USA), was tested in parallel throughout bioassay-guided fractionation as an internal control for compound delivery. Following structure elucidation and chemical identification, active molecules were assessed in duplicate 10-point dose–response format against all three cell lines, and the background-normalized data were fit to the following equation using GraphPad Prism (Version 11.0) software to determine the LD_50_ of each compound against each cell line using nonlinear regression with a least squares fit:

% Viability=100(1+LD50CompoundHillSlope)

### 4.5. Computational Methods

Following the conformational analysis using MMFF94 as a force field and the GMMX methodology, geometrical optimization and energy calculation of conformers occurring in an energy window (Δ*ε*) of 0–5 Kcal/mol was done by implementation of CAM-B3LYP/DGDZVP in MeOH in Gaussian 16 software. The optimized structures (Table S3) were used to calculate the thermochemical parameters estimated at 298 K and 1 atm. Starting from DFT-optimized structures, calculations in MeOH were performed. Optimized conformers were then subjected to DFT calculations on Gaussian 16 using CAM-B3LYP/DGDZVP in MeOH to obtain the ECD spectra. Obtained values were Boltzmann-averaged and compared with the experimental values. All quantum-mechanical calculations were carried out using the Gaussian 16 software on a Linux operating system in the Biowulf cluster [34]. Obtained ECD spectra with a half-band of 0.3 eV and UV shift of 38 nm were Boltzmann averaged and scaled using the SpecDis program and compared with experimental spectra obtained in MeOH.

## Figures and Tables

**Table 1:** NMR spectroscopic data for microspinosamide B (**1**) in DMF-*d*_7_ (600 MHz for ^1^H, 150 MHz for ^13^C, and 60 MHz for ^15^N, *δ* in ppm).

Amino Acid	Position	*δ*_C_*/δ*_N_ ^a^, Type	*δ*_H_, Mult (*J* in Hz)	HMBC	ROESY
ᴅ-alanine (Ala)	HCO	162.0, C	8.05, s	47.9	8.18
	*N*H	^b^	8.18, d (6.6)	47.9, 162.0	0.93, 4.40, 8.05
	CO	172.6, C	-	-	-
	*α*	47.9, CH	4.40, m	19.2, 162.0, 172.6	0.93, 8.18, 8.26
	*β*	19.2, CH_3_	0.93 s	47.9, 172.6	4.40, 8.18
*β*-hydroxy-*p-*bromophenylalanine (*β*-OH-*p*-BrPhe)	*N*H	112.8, *N*H	8.26, s	57.6, 172.6	4.40, 4.87
	CO	172.0, C	-	-	-
	*α*	57.6, CH	4.87, ^b^	75.5, 142.5, 172.0	3.82, 4.02, 8.26
	*β*	75.5, CH	4.87, ^b^	57.6	7.53
	C-1	142.5, C	-	-	-
	C-2/C-6	130.9, CH	7.53, d (8.2)	75.5, 122.0, 132.0	4.87
	C-3/C-5	132.0, CH	7.56, d (8.2)	130.9, 142.5	4.87
	C-4	122.0, C	-	-	-
ʟ-proline I (Pro I)	CO	173.2, C	-	-	-
	*α*	61.4, CH	4.91, m	25.0, 30.8	1.95, 1.97, 2.29, 8.11
	*β*	30.8, CH_2_	2.29, m 1.97, m	25.0, 48.2, 61.4, 173.2	4.03, 4.91
	*γ*	25.0, CH_2_	1.95, m	30.8, 48.2, 61.4	3.82, 4.03, 4.91
	*δ*	48.2, CH_2_	4.03, m 3.82, m	25.0, 30.8, 61.4	1.95, 1.97, 4.87
ᴅ-*t*-Leucine I (*t*-Leu I)	*N*H	114.4, *N*H	8.11, d (9.4)	173.2	1.00, 4.70, 4.91
	CO	172.8, C	-	-	-
	*α*	61.1, CH	4.70, d (9.4)	27.7, 36.1, 172.8	1.00, 8.11, 8.19
	*β*	36.1, C	-	-	-
	*γ*	27.7, CH_3_	1.00, s	27.7, 36.1, 61.1	4.70, 8.11
ʟ-*t*-leucine II (*t*-Leu II)	*N*H	^b^	8.19, d (6.6)	172.8	4.70
	CO	171.7, C	-	-	-
	*α*	63.5, CH	4.18, d (6.6)	27.6, 34.0, 171.7	0.86, 8.45
	*β*	34.0, C	-	-	-
	*γ*	27.6, CH_3_	0.86, s	27.6, 34.0, 63.5	4.18
ᴅ-tryptophan (Trp)	*N*H-indole	129.3, *N*H	10.85, s	112.4, 125.2, 128.8, 137.9	7.30, 7.39
	*N*H	125.3, *N*H	8.45, d (4.5)	54.8, 171.7	4.18
	CO	172.7, C	-	-	-
	*α*	54.8, CH	4.84, m	28.6, 111.6, 172.7	3.14, 3.37, 7.30
	*β*	28.6, CH_2_	3.37, dd (14.6, 5.8) 3.14, m	111.6, 128.8, 172.7	4.84, 7.30, 7.60
	C-2	125.2, CH	7.30, s	128.8, 137.9	3.14, 3.37, 4.84, 10.85
	C-3	111.6, C	-	-	-
	C-3*α*	128.8, C	-	-	-
	C-4	119.4, CH	7.60, d (8.0)	111.6, 122.0, 137.9	3.14, 3.37
	C-5	119.5, CH	6.99, t (8.0)	112.4, 128.8	7.39
	C-6	122.0, CH	7.08, t (8.0)	119.4, 137.9	7.39
	C-7	112.4, CH	7.39, d (8.0)	119.5, 128.8	6.99, 7.08, 10.85
	C-7*α*	137.9, C	-	-	-
ʟ-arginine (Arg)	*N*H-guanidine	85.4, *N*H	7.82, t (4.8)	41.9, 158.7	3.17
	C-guanidine	158.7, C	-	-	-
	*N*H	118.3, *N*H	8.33, br s	172.7	-
	CO	173.0, C	-	-	-
	*α*	55.2, CH	4.34, m	29.8, 173.0	-
	*β*	29.8, CH_2_	1.88, m 1.83, m	25.5, 41.9, 55.2	-
	*γ*	25.5, CH_2_	1.56, m	29.8, 41.9, 55.2	-
	*δ*	41.9, CH_2_	3.17, m	25.5, 29.8	7.82
ᴅ-cysteic acid (Cya)	*N*H	112.4, *N*H	8.20, ^b^	173.0	4.87
	CO	171.8, C	-	-	-
	*α*	52.8, CH	4.87, m	52.1, 171.8	3.23, 8.00, 8.20
	*β*	52.1, CH_2_	3.23, m 3.14, m	52.8, 171.8	4.87
ʟ-threonine (Thr)	*N*H	108.9, *N*H	8.00, ^b^	171.8	4.87, 5.04
	CO	170.6, C	-	-	-
	*α*	53.5, CH	5.04, dd (7.8, 2.0)	72.1, 170.6, 171.8	1.35, 3.11, 8.00
	*β*	72.1, CH	5.13, m	16.4, 53.5, 170.6, 171.5	1.35
	*γ*	16.4, CH_3_	1.35, d (6.6)	53.5, 72.1	5.04, 5.13
ʟ-*N*-methylglutamine (*N*MeGln)	*N*Me	31.5, CH_3_	3.11, s	57.1, 170.6	2.13, 5.04
	CO	171.1, C	-	-	-
	O=C-*N*H_2_	106.5, *N*H	7.43, br s	175.1	2.24
	O=C-*N*H_2_	175.1, C	-	-	-
	*α*	57.1, CH	5.33, t (7.8)	24.6, 32.7, 170.6, 171.1	2.13, 2.33
	*β*	24.6, CH_2_	2.13, m	32.7, 57.1, 171.1	5.33
	*γ*	32.7, CH_2_	2.33, m 2.24, m	24.6, 175.1	5.33
ᴅ-valine (Val)	*N*H	114.2, *N*H	7.38, br s	-	-
	CO	171.3, C	-	-	-
	*α*	56.7, CH	4.58, br t (7.2)	20.5, 31.5	0.94, 2.08, 3.65, 3.94
	*β*	31.5, CH	2.08, m	20.5, 56.7, 171.3	0.94, 4.58
	*γ*	18.4, CH_3_	0.94 d (6.6)	20.5, 31.5, 56.7	2.08, 4.58
	*γ*′	20.5, CH_3_	0.94, d (6.6)	18.4, 31.5, 56.7	2.08, 4.58
ʟ-proline II (Pro II)	CO	172.5, C	-	-	-
	*α*	62.0, CH	4.34, m	25.0, 30.8, 172.5	1.95, 2.15, 3.65
	*β*	30.8, CH_2_	2.15, m 1.95, m	25.0, 48.9, 172.5	3.65, 3.94, 4.34
	*γ*	25.0, CH_2_	1.95, m	30.8, 48.9	3.65, 3.94, 4.34
	*δ*	48.9, CH_2_	3.94, br t (8.2) 3.65, m	25.0, 30.8, 62.0	1.95, 2.15, 4.34
ᴅ-aspartic acid (Asp)	*N*H	112.0, *N*H	7.64, d (8.4)	172.5	4.81
	CO	171.5, C	-	-	-
	COOH	173.4, C	-	-	-
	*α*	50.3, CH	4.81, m	37.1, 171.5	2.77, 2.86, 7.64
	*β*	37.1, CH_2_	2.86, m 2.77, m	50.1, 50.3	4.81

^a^ Assignments were extracted from ^1^H–^15^N HSQC and HMBC spectra. ^b^ Signal overlap prevents the determination of coupling constants or chemical shifts.

**Table 3:** LD_50_ values of **1**–**5** against DPM cell lines. Data shown are the mean of three replicates of one experiment (two experiments per compound were run).

Compound	MB24 (μM)	MB52 (μM)	NP1 (μM)
**1**	**0.392** (0.309–0.493)	**0.950** (0.606–1.369)	**0.670** (0.540–0.820)
**2**	**0.088** (0.079–0.097)	**0.250** (0.206–0.301)	**0.269** (0.226–0.318)
**3**	**0.031** (0.024–0.038)	**0.066** (0.048–0.091)	**0.078** (0.058–0.105)
**4**	**3.757** (2.814–4.884)	**5.857** (4.104–8.132)	**6.077** (4.596–7.916)
**5**	**1.911** (1.609–2.271)	**7.118** (6.069–8.304)	**4.460** (3.666–5.521)
**Positive Control** **Sanguinarine Chloride**	**0.992** (0.851–1.132)	**1.058** (0.940–1.175)	**1.005** (0.817–1.192)

## Data Availability

The NMR data for the following compounds have been deposited in the Natural Products Magnetic Resonance Database accessed on 29 July 2026 (NP-MRD; www.np-mrd.org) and can be found at NP 0354334 (microspinosamide B), NP 0354335 (microspinosamide C), NP0354336 (microspinosamide D), and NP 0354337 ((2*R*,3*R*)-3-(4-bromophenyl)-2-((2,4-dinitrophenyl)amino)-3-hydroxypropanoic acid)).

## Supplementary Materials

The following Supporting Information can be downloaded at https://www.mdpi.com/article/10.3390/md24090308/s1, The Supporting Information is available free of charge at PubMed Central: HRESIMS, NMR, and IR spectra of **1**, **4**, **5**, **6**; physical and spectroscopic data of the co-occurring known depsipeptides **2** and **3**, and LD_50_ viability curves for compounds **1**–**5**.

## Abbreviations

The following abbreviations are used in this manuscript:

DPM	diffuse pleural mesothelioma
Ala	alanine
Asp	aspartic acid
Trp	tryptophan
Val	valine
Arg	arginine
Pro	proline
Thr	threonine
*β*-OH-*p*-BrPhe	*β*-hydroxy-*p*-bromophenylalanine
Cya	cysteic acid
*N*MeGln	*N*-methylglutamine
Leu	leucine
FDLA	1-fluoro-2,4-dinitrophenyl-5-ʟ-leucinamide
DNFB	1-fluoro-2,4-dinitrobenzene
TDDFT	time-dependent density functional theory
ECD	electronic circular dichroism
