# Supplementary material for: Cytotoxic Microspinosamides from the Marine Sponge Geodia microspinosa That Decrease Diffuse Pleural Mesothelioma Viability
Source: Mar Drugs. 2026 Sep 4;24(9):308. doi: 10.3390/md24090308 (PMC13608206; doi:10.3390/md24090308)
Supplement: Supplementary file 1 [file marinedrugs-24-00308-s001.zip › marinedrugs-4527446-supplementary.pdf]

Supporting Information for

Cytotoxic Microspinosamides from the Marine Sponge *Geodia microspinosa* that Decrease Diffuse Pleural Mesothelioma Viability

Maria Orfanoudaki[1], Dongdong Wang[1], Lin Du[1], Vivek Singh[2], Ekaterina I. Goncharova[3], Nathanael Pruett[2], Chuong D. Hoang[2], Brice A. P. Wilson[1,*] and Barry R. O'Keefe[1,4]

[1]Molecular Targets Program, Center for Cancer Research, National Cancer Institute, Frederick, Maryland, 21702-1201, United States; orfmaria@gmail.com (M.O.); dongdong.wang@nih.gov (D.W.); lin.du@nih.gov (L.D.); brice.wilson@nih.gov (B.W.), okeefeba@nih.gov (B.O.K.)

[2]Thoracic Surgery Branch, National Cancer Institute, National Institutes of Health, Bethesda, Maryland 20892, United States; vivek.singh@nih.gov (V.S.); nathanael.pruett@nih.gov (N.P.); chuong.hoang@nih.gov (C.H.)

[3]Advanced Biomedical Computational Science, Frederick National Laboratory for Cancer Research, Frederick, Maryland 21702-1201, United States; Katya.Goncharova@nih.gov (E.G.)

[4]Natural Products Branch, Division of Cancer Treatment and Diagnosis, National Cancer Institute, Frederick, Maryland 21702-1201, United States

* Correspondence: brice.wilson@nih.gov

Table of Contents

Table S1. Summary of Marfey's analysis for compounds 1-5. 5

Table S2. NMR Spectroscopic data for microspinosamide C (4) in DMF-d_7 (600 MHz for ^1H, 150 MHz for ^{13}C, and 60 MHz for ^{15}N, δ in ppm). 6

Table S3. Population of Boltzmann averaged conformers of compound (2*S*,3*S*)-6. 8

Figure S1. HR-MS analysis of the bioactive fraction 3 used for the isolation of the peptides. 9

Figure S2. Conversion of compound 1 (0.1 mg/mL) to 4 after 5 days of dissolution in various solvents at room temperature. 10

Figure S3. LD_{50} curves for compound 1 in DPM and normal pleura (NP1) cell lines. 11

Figure S4. LD_{50} curves for compound 2 in DPM and normal pleura (NP1) cell lines. 12

Figure S5. LD_{50} curves for compound 3 in DPM and normal pleura (NP1) cell lines. 13

Figure S6. LD_{50} curves for compound 4 in DPM and normal pleura (NP1) cell lines. 14

Figure S7. LD_{50} curves for compound 5 in DPM and normal pleura (NP1) cell lines. 15

Figure S8. Crucial 2D NMR correlations used to establish the structure of the new natural product 4 in DMF-d_7. 16

Figure S9. ^1H NMR spectrum (600 MHz, DMF-d_7) of 1. The spectrum was acquired over 4 scans 17

Figure S10. ^{13}C NMR spectrum (150 MHz, DMF-d_7) of 1. The spectrum was acquired over 25000 scans. 18

Figure S11. ^1H-^1H COSY spectrum of 1 in DMF-d_7. The spectrum was acquired over 32 scans. 19

Figure S12. ^1H-^{13}C HSQC spectrum of 1 in DMF-d_7. The spectrum was acquired over 64 scans. 20

Figure S13. ^1H-^{13}C HMBC spectrum of 1 in DMF-d_7. The spectrum was acquired over 1760 scans. 21

Figure S14. ^1H-^1H ROESY spectrum of 1 in DMF-d_7. The spectrum was acquired over 560 scans. 22

Figure S15. ^1H-^{13}C HSQC-TOCSY spectrum of 1 in DMF-d_7. The spectrum was acquired over 96 scans. 23

Figure S16. ^1H-^{15}N HSQC spectrum of 1 in DMF-d_7. The spectrum was acquired over 96 scans. 24

Figure S17. HRESIMS spectrum of 1. 25

Figure S18. Positive HR-ESI-MS-MS b and y ion fragments of 1 26

Figure S19. IR spectrum of 1 27

Figure S20. ^{1}H NMR spectrum (600 MHz, DMF-d_7) of 2. The spectrum was acquired over 4 scans. 28

Figure S21. ^{1}H NMR spectrum (600 MHz, MeOD-d_4) of 2. The spectrum was acquired over 4 scans. 29

Figure S22. ^{13}C NMR spectrum (150 MHz, DMF-d_7) of 2. The spectrum was acquired over 4000 scans. 30

Figure S23. ^{13}C NMR spectrum (150 MHz, MeOD-d_4) of 2. The spectrum was acquired over 4000 scans. 31

Figure S24. ^{1}H NMR spectrum (600 MHz, DMSO-d_6) of 3. The spectrum was acquired over 4 scans. 32

Figure S25. ^{13}C NMR spectrum (150 MHz, DMSO-d_6) of 3. The spectrum was acquired over 30000 scans. 33

Figure S26. ^{1}H NMR spectrum (600 MHz, DMF-d_7) of 4. The spectrum was acquired over 4 scans. 34

Figure S27. ^{13}C NMR spectrum (150 MHz, DMF-d_7) of 4. The spectrum was acquired over 12000 scans. 35

Figure S28. ^{1}H-^{1}H COSY spectrum of 4 in DMF-d_7. The spectrum was acquired over 16 scans. 36

Figure S29. ^{1}H-^{13}C HSQC spectrum of 4 in DMF-d_7. The spectrum was acquired over 32 scans. 37

Figure S30. ^{1}H-^{13}C HMBC spectrum of 4 in DMF-d_7. The spectrum was acquired over 560 scans. 38

Figure S31. ^{1}H-^{1}H ROESY spectrum of 4 in DMF-d_7. The spectrum was acquired over 128 scans. 39

Figure S32. ^{1}H-^{13}C HSQC-TOCSY spectrum of 4 in DMF-d_7. The spectrum was acquired over 48 scans. 40

Figure S33. ^{1}H-^{15}N HSQC spectrum of 4 in DMF-d_7. The spectrum was acquired over 48 scans. 41

Figure S34. HRESIMS spectrum of 4. 42

Figure S35. IR spectrum of 4. 43

Figure S36. ^{1}H NMR spectrum (600 MHz, DMF-d_7) of 5. The spectrum was acquired over 4 scans. 44

Figure S37. ^{13}C NMR spectrum (150 MHz, DMF-d_7) of 5. The spectrum was acquired over 35000 scans. 45

Figure S38. ^{1}H-^{1}H COSY spectrum of 5 in DMF-d_7. The spectrum was acquired over 200 scans. 46

Figure S39. ^{1}H-^{13}C HSQC spectrum of 5 in DMF-d_7. The spectrum was acquired over 64 scans. 47

Figure S40. ^{1}H-^{13}C HMBC spectrum of 5 in DMF-d_7. The spectrum was acquired over 1360 scans. 48

Figure S41. ^{1}H-^{1}H ROESY spectrum of 5 in DMF-d_7. The spectrum was acquired over 528 scans. 49

Figure S42. ^{1}H-^{13}C HSQC-TOCSY spectrum of 5 in DMF-d_7. The spectrum was acquired over 80 scans. 50

Figure S43. ^{1}H-^{15}N **HSQC spectrum of 5 in DMF-d_7. The spectrum was acquired over 128 scans.** 51
Figure S44. HRESIMS spectrum of 5. 52
Figure S45. Positive HR-ESI-MS-MS b and y ion fragments of 5. 53
Figure S46. IR spectrum of 5. 54
Figure S47. ^{1}H **NMR spectrum (600 MHz, DMSO-d_6) of 6. The spectrum was acquired over 4 scans.** 55
Figure S48. ^{13}C **NMR spectrum (150 MHz, DMSO-d_6) of 6. The spectrum was acquired over 15000 scans.** 56
Figure S49. ^{1}H-^{1}H **COSY spectrum of 6 in DMSO-d_6. The spectrum was acquired over 16 scans.** 57
Figure S50. ^{1}H-^{13}C **HSQC spectrum of 6 in DMSO-d_6. The spectrum was acquired over 32 scans** 58
Figure S51. ^{1}H-^{13}C **HMBC spectrum of 6 in DMSO-d_6. The spectrum was acquired over 420 scans.** 59
Figure S52. ^{1}H-^{1}H **ROESY spectrum of 6 in DMSO-d_6. The spectrum was acquired over 160 scans.** 60
Figure S53. HRESIMS spectrum of 6. 61
Figure S54. HRESIMS spectrum of 6 (zoomed). 62
Figure S55. IR spectrum of 6. 63

Table S1. Summary of Marfey's analysis for compounds 1-5.

Amino acid	Retention time	compound **1**	compound **2**	compound **3**	compound **4**	compound **5**
m/*z* 384						
L-alanine	25.4					
D-alanine	30.5	30.5	30.5	30.5	30.6	30.6
m/*z* 426						
L-tert-leucine	32.2	32.3				
D-tert-leucine	41.2	41.3	41.3	41.3	41.3	41.4
m/*z* 440						
L-β methyl-isoleucine	35.5		35.4	35.4	35.4	35.4
D-β methyl-isoleucine	44.6					
m/*z* 410						
L-proline	26.3	26.3	26.3	26.2	26.2	26.4
D-proline	29.4					
m/*z* 503						
L-kynurenine (L-tryptophan)	33.2					
D-kynurenine (D-tryptophan)	39.3	39.4	39.4	39.4	39.4	39.4
m/*z* 469						
L-arginine	19.9	19.9	19.9	19.9	19.9	20.0
D-arginine	19.7					
m/*z* 464						
L-cysteic acid	17.7					
D-cysteic acid	17.3	17.3	17.3	17.4	17.2	17.3
m/*z* 412						
L-valine	29.9					
D-valine	38.0	38.1	38.2	38.1	38.2	38.2
m/*z* 414						
L-threonine	21.4	21.4	21.4	21.3	21.4	21.4
D-threonine	27.6					
L-allo-threonine	22.8					
D-allo-threonine	25.0					
m/*z* 456						
L-N-methylglutamic acid	24.3	24.3	24.2	24.2	24.2	24.3
D-N-methylglutamic acid	24.9					
m/*z* 428						
L-aspartic acid	22.8					
D-aspartic acid	25.0	24.9	24.9	24.9	24.9	24.9
m/*z* 554/556						
β-hydroxy-p-bromophenylalanine		35.2	35.2	35.2	35.2	35.2

Table S2. **NMR Spectroscopic data for microspinosamide C (4) in DMF-*d*$_7$ (600 MHz for ^{1}H, 150 MHz for ^{13}C, and 60 MHz for ^{15}N, *δ* in ppm).**

amino acid	position	δ_C/δ_N^{a}, type	δ_H, mult (*J* in Hz)	HMBC	ROESY
D-alanine (Ala)	*N*H$_2$	125.3, *N*H	8.43 br s	-	4.15
	CO	170.5, C	-	-	-
	α	50.1, CH	4.15, m	18.0, 170.5	1.19, 8.43, 8.82
	β	18.0, CH$_3$	1.19, d (6.4)	50.1, 170.5	4.15
β-hydroxy-*p*-bromophenylalanine (*β*-OH-BrPhe)	*N*H	113.9, *N*H	8.82, d (9.2)	170.5	4.15, 4.84, 7.54
	CO	170.5, C	-	-	-
	α	57.5, CH	4.96, t (9.2)	75.5, 142.3, 170.5	3.77, 4.05, 7.54
	β	75.5, CH	4.84 (9.2)	57.5, 130.9, 142.3	7.54, 7.57, 8.82
	C-1	142.3, C	-	-	-
	C-2/C-6	130.9, CH	7.54, d (8.2)	75.5, 122.1, 130.9	4.84, 4.96, 8.82
	C-3/C-5	132.0, CH	7.57, d (8.2)	122.1, 132.0, 142.3	4.84
	C-4	122.1, C	-	-	-
L-proline I (Pro I)	CO	172.8, C	-	-	-
	α	61.4, CH	4.79, m	25.4, 172.8	1.98, 2.28, 8.10
	β	30.4, CH$_2$	2.28, m<br>2.15, m	25.4, 172.8	4.79
	γ	25.4, CH$_2$	1.98, m	30.4, 48.8, 61.4	4.05, 4.79
	δ	48.8, CH$_2$	4.05, m<br>3.77, t (7.2)	25.4, 30.4, 61.4	1.98, 4.96
L-*t*-leucine (*t*-Leu)	*N*H	114.4, *N*H	8.10, d (7.8)	172.8	4.79
	CO	172.9, C	-	-	-
	α	61.5, CH	4.69, d (7.8)	27.7, 36.3	1.00, 8.07
	β	36.3, C	-	-	-
	γ	27.7, CH$_3$	1.00, s	27.7, 36.3, 61.5	4.69
L-*β*-methylisoleucine (*β*-MeIle)	*N*H	121.0, *N*H	8.07, ^{b}	172.2	4.69
	CO	172.2, C	-	-	-
	α	62.0, CH	4.27, br s	23.7, 32.6, 36.9, 172.2	0.72, 0.76, 0.86, 8.07
	β	36.9, C	-	-	-
	γ	32.6, CH$_2$	1.23, m	8.8, 23.9, 36.9	0.72
	δ	8.8, CH$_3$	0.72, t (8.0)	32.6, 36.9	1.23, 4.27
	γ′	23.7, CH$_3$	0.76, s	23.9, 36.9, 62.0	4.27
	γ″	23.9, CH$_3$	0.86, s	23.7, 32.6, 36.9, 62.0	4.27
D-tryptophan (Trp)	*N*H-indole	129.6, *N*H	10.91, s	111.5, 125.3, 128.7, 137.9	7.30, 7.41
	*N*H	125.3, *N*H	8.52, d (7.0)	172.2	4.27
	CO	172.6, C	-	-	-
	α	55.5, CH	4.74, m	28.6, 111.5	3.16, 3.18, 7.30, 8.15
	β	28.6, CH$_2$	3.38, m<br>3.16, m	55.5, 111.5, 125.3, 128.7, 172.6	4.74
	C-2	125.3, CH	7.30, s	28.6, 111.5, 128.7, 137.9	10.91
	C-3	111.5, C	-	-	-
	C-3*α*	128.7, C	-	-	-
	C-4	119.5, CH	7.63, d (7.8)	111.5, 122.0, 128.7, 137.9	4.74, 7.00
	C-5	119.5, CH	7.00, t (7.8)	112.5, 128.7	7.63
	C-6	122.0, CH	7.08, t (7.8)	119.5, 137.9	7.41
	C-7	112.5, CH	7.41, d (7.8)	119.5, 128.7	7.08

	C-7α	137.9, C	-	-	-
L-arginine (Arg)	*N*H-guanidine	85.0, *N*H	7.93, m	41.9	-
	C-guanidine	158.9, C	-	-	-
	*N*H	117.5, *N*H	8.15, [b]	172.6	4.74
	CO	172.8, C	-	-	-
	α	54.5, CH	4.48, q (7.0)	25.4, 29.9, 172.8	8.34
	β	29.9, CH$_2$	1.80, m	25.4, 41.9, 54.5, 172.8	-
	γ	25.4, CH$_2$	1.51, m	29.9, 41.9, 54.5	-
	δ	41.9, CH$_2$	3.18, m	25.4, 29.9, 158.9	-
D-cysteic acid (Cys)	*N*H	113.9, *N*H	8.34, d (6.6)	172.8	4.48
	CO	172.1, C	-	-	-
	α	52.5, CH	4.83, m	52.9,	3.16, 8.13
	β	52.9, CH$_2$	3.16, m	52.5, 172.1	4.83
L-threonine (Thr)	*N*H	109.3, *N*H	8.13, [b]	172.1	4.83
	CO	170.8, C	-	-	-
	α	53.5, CH	5.08, d (7.0)	71.7, 170.8, 172.1	1.33, 3.13
	β	71.7, CH	5.21, m	16.7, 53.5, 170.8, 172.1	1.33, 3.13
	γ	16.7, CH$_3$	1.33, d (6.2)	53.5, 71.7	5.08, 5.21
L-*N*-methylglutamine (NMeGln)	*N*Me	31.5, CH$_3$	3.13, s	56.8, 170.8	5.08, 5.21
	CO	171.5, C	-	-	-
	O=C-<u>*N*H$_2$</u>	106.5, *N*H	7.43, br s<br>6.74, s	175.3	-
	O=<u>C</u>-*N*H$_2$	175.3, C	-	-	-
	α	56.8, CH	5.25, m	25.1	2.19
	β	25.1, CH$_2$	2.08, m<br>2.19, m	32.8, 56.8, 171.3, 175.3	-
	γ	32.8, CH$_2$	2.20, m	25.1, 56.8, 175.3	5.25
D-valine (Val)	*N*H	115.5, *N*H	7.57, [b]	-	-
	CO	171.8, C	-	-	-
	α	56.8, CH	4.55, t (7.0)	18.5, 20.3, 31.0, 171.8	0.90, 2.06, 3.66, 3.97
	β	31.0, CH	2.06, m	18.5, 20.3	0.90, 0.94, 3.66, 4.55
	γ	18.5, CH$_3$	0.90, d (6.4)	20.3, 31.0, 56.8	2.06, 4.55
	γ′	20.3, CH$_3$	0.94, d (6.4)	18.5, 31.0, 56.8	2.06
L-proline II (Pro II)	CO	172.3, C	-	-	-
	α	61.7, CH	4.34, d (7.6)	24.9, 30.5, 48.2, 172.3	1.98, 2.15
	β	30.5, CH$_2$	2.15, m	24.9, 172.3	3.97, 4.34
	γ	24.9, CH$_2$	1.98, m	61.7	3.66
	δ	48.2, CH$_2$	3.97, m<br>3.66, t (8.2)	24.9, 30.5	1.98, 2.15
	*N*H	111.6, *N*H	7.54, [b]	172.3	-
	CO	171.7, C	-	-	-
D-aspartic acid (Asp)	COOH	173.3, C	-	-	-
	α	50.1, CH	4.76, m	171.7, 173.3	2.72, 2.92
	β	36.8, CH$_2$	2.92, m<br>2.70, m	50.1, 171.7	4.76

[a]Assignments were based on ^{1}H–^{15}N HSQC correlations,

[b]Signal overlap prevents determination of couplings or chemical shift.

Table S3. Population of Boltzmann averaged conformers of compound (2*S*,3*S*)-**6**.

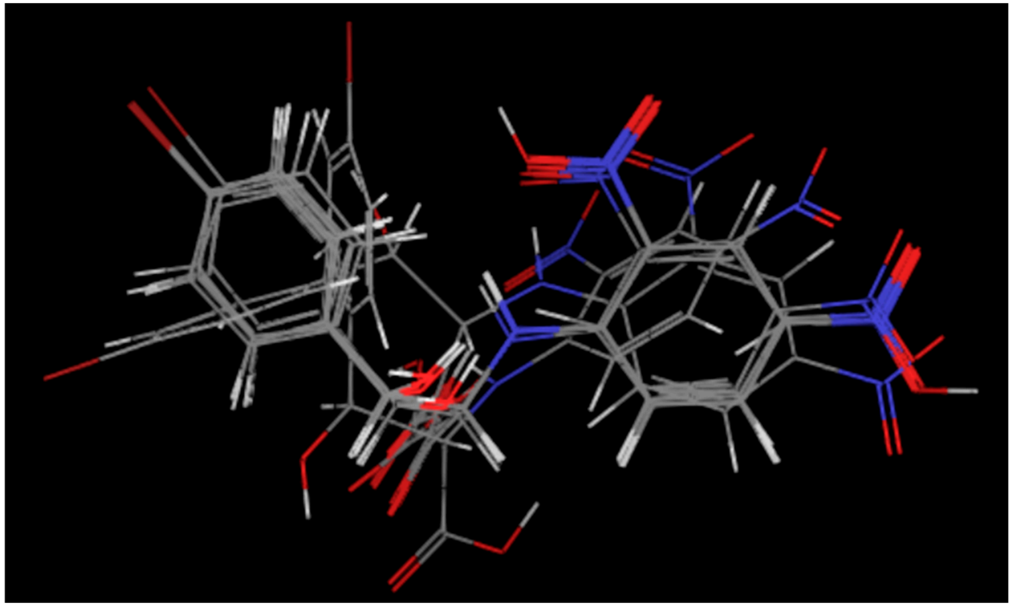

Index	E(au)	E(kcal/mol)	DeltaE(kcal/mol)	Qi(Relat)	Population
conformer 1	-3843.353673	-2411740.941	1.589607066	0.068240844	2.43%
conformer 2	-3843.353532	-2411740.853	1.678026919	0.05877459	2.09%
conformer 3	-3843.355053	-2411741.808	0.723330201	0.29474533	10.49%
conformer 4	-3843.356206	-2411742.531	0	1	35.58%
conformer 5	-3843.355105	-2411741.84	0.690699707	0.311444715	11.08%
conformer 6	-3843.354765	-2411741.627	0.904052936	0.2172147	7.73%
conformer 7	-3843.355645	-2411742.179	0.351970078	0.551867268	19.63%
conformer 8	-3843.355096	-2411741.835	0.696410043	0.308455494	10.97%

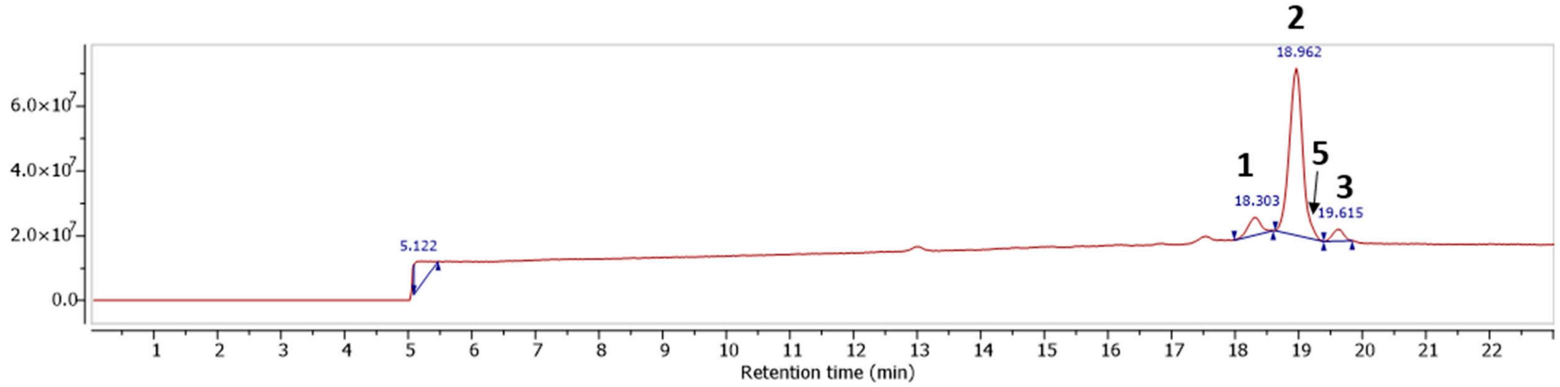

Figure S1. HR-MS analysis of the bioactive fraction 3 used for the isolation of the peptides.

Solvents	conversion of 1 to 4
DMSO	-
DMF-*d7*	-
MeOH	Traces (EIC)
ACN	-
H_2O	-
H_2O/ACN, 1/1, v/v	Traces (EIC)
H_2O/MeOH, 1/1, v/v	Traces (EIC)
H_2O/ACN, 1/1, v/v + 0.1% FA	Traces (EIC)
H_2O/MeOH, 1/1, v/v + 0.1% FA	Traces (EIC)
H_2O/ACN, 1/1, v/v + 0.1% TFA	10% conversion
H_2O/MeOH, 1/1, v/v + 0.1% TFA	40% conversion

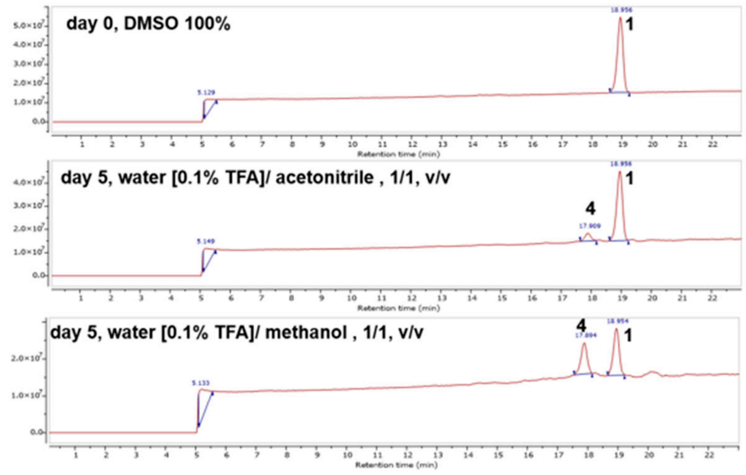

Figure S2. Conversion of compound **1** (0.1 mg/mL) to **4** after 5 days of dissolution in various solvents at room temperature.

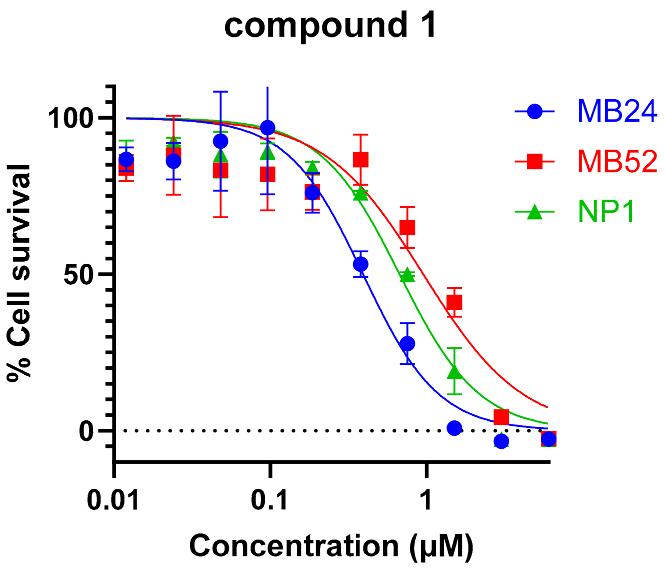

Figure S3. LD_{50} curves for compound **1** in DPM and normal pleura (NP1) cell lines.

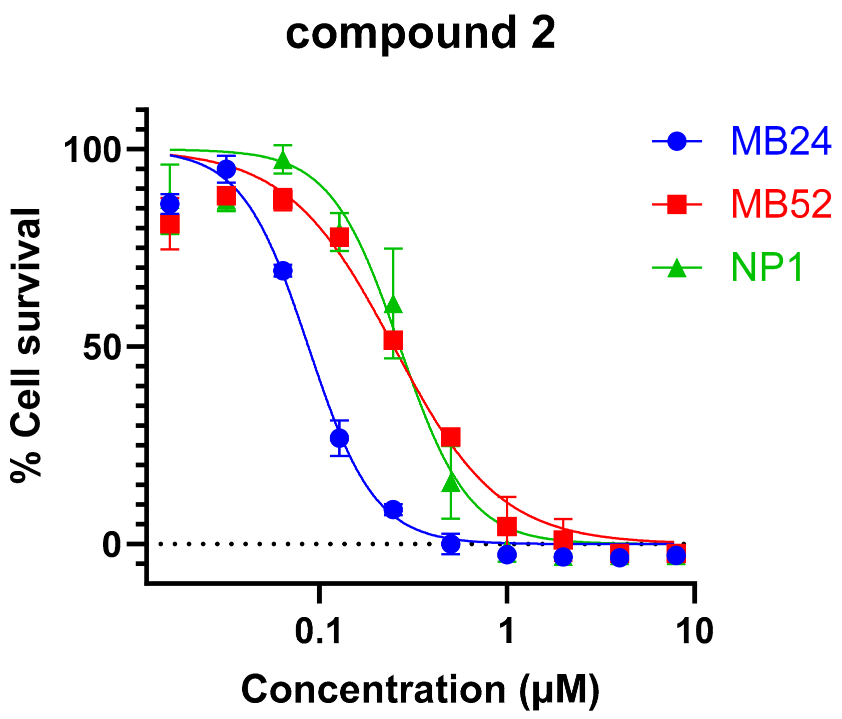

Figure S4. LD_{50} curves for compound **2** in DPM and normal pleura (NP1) cell lines.

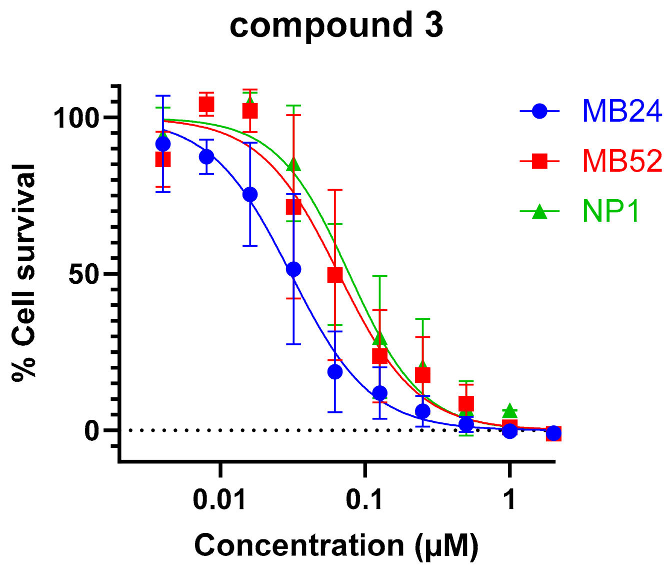

Figure S5. LD_{50} curves for compound **3** in DPM and normal pleura (NP1) cell lines.

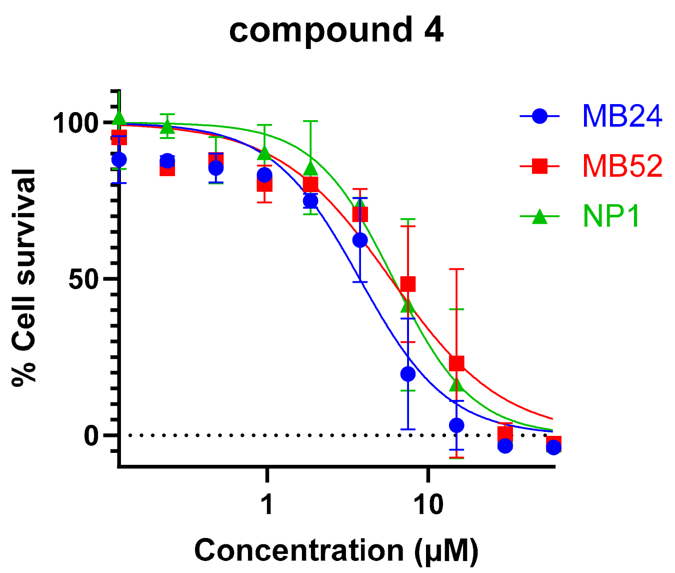

Figure S6. LD_{50} curves for compound **4** in DPM and normal pleura (NP1) cell lines.

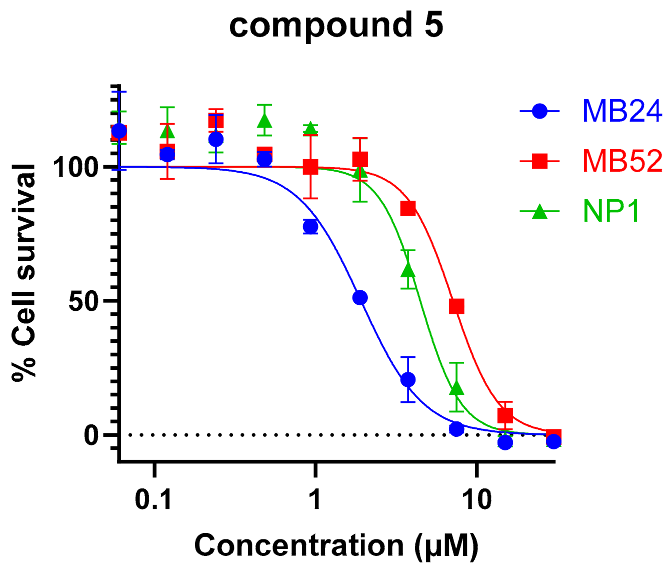

Figure S7. LD_{50} curves for compound **5** in DPM and normal pleura (NP1) cell lines.

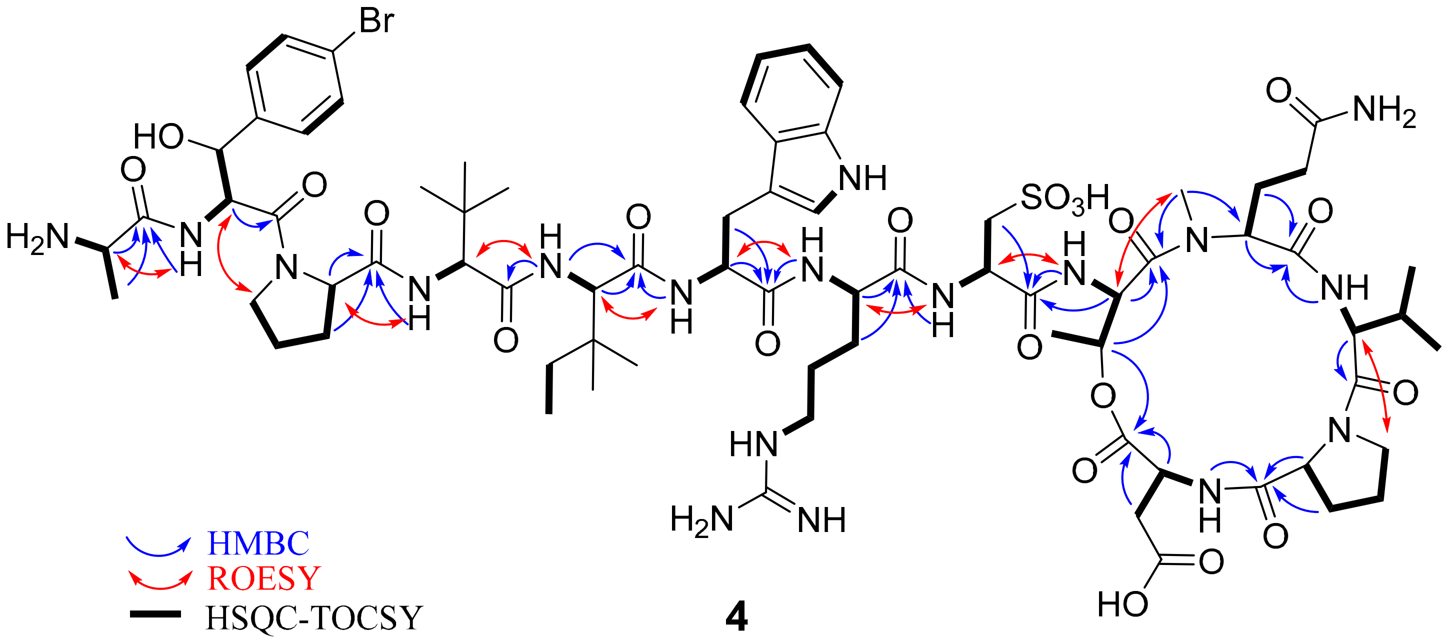

4

Figure S8. Crucial 2D NMR correlations used to establish the structure of the new natural product **4** in DMF-d_7.

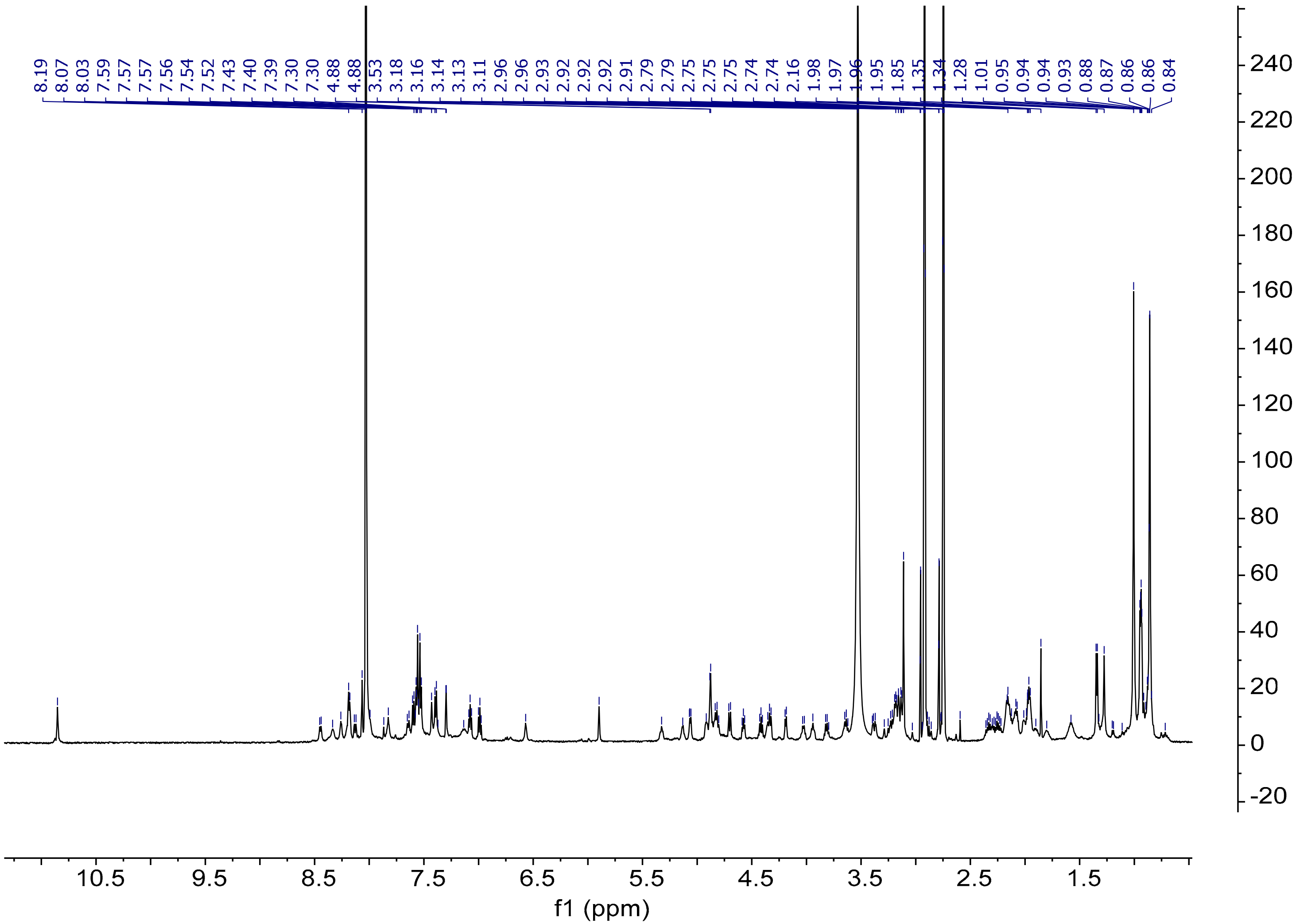

Figure S9. ^{1}H NMR spectrum (600 MHz, DMF-d_7) of **1**. The spectrum was acquired over 4 scans.

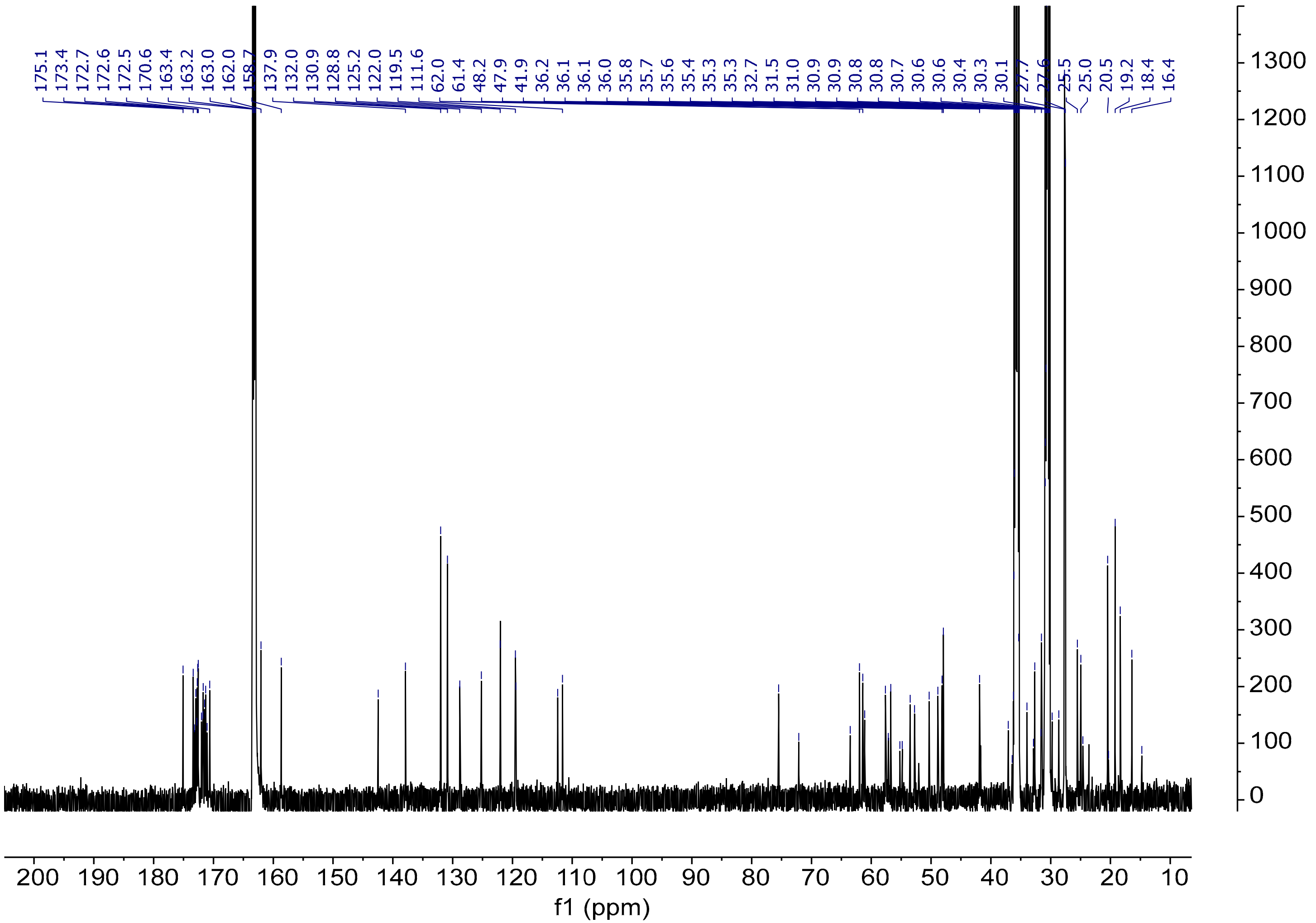

Figure S10. ^{13}C NMR spectrum (150 MHz, DMF-d_7) of **1**. The spectrum was acquired over 25000 scans.

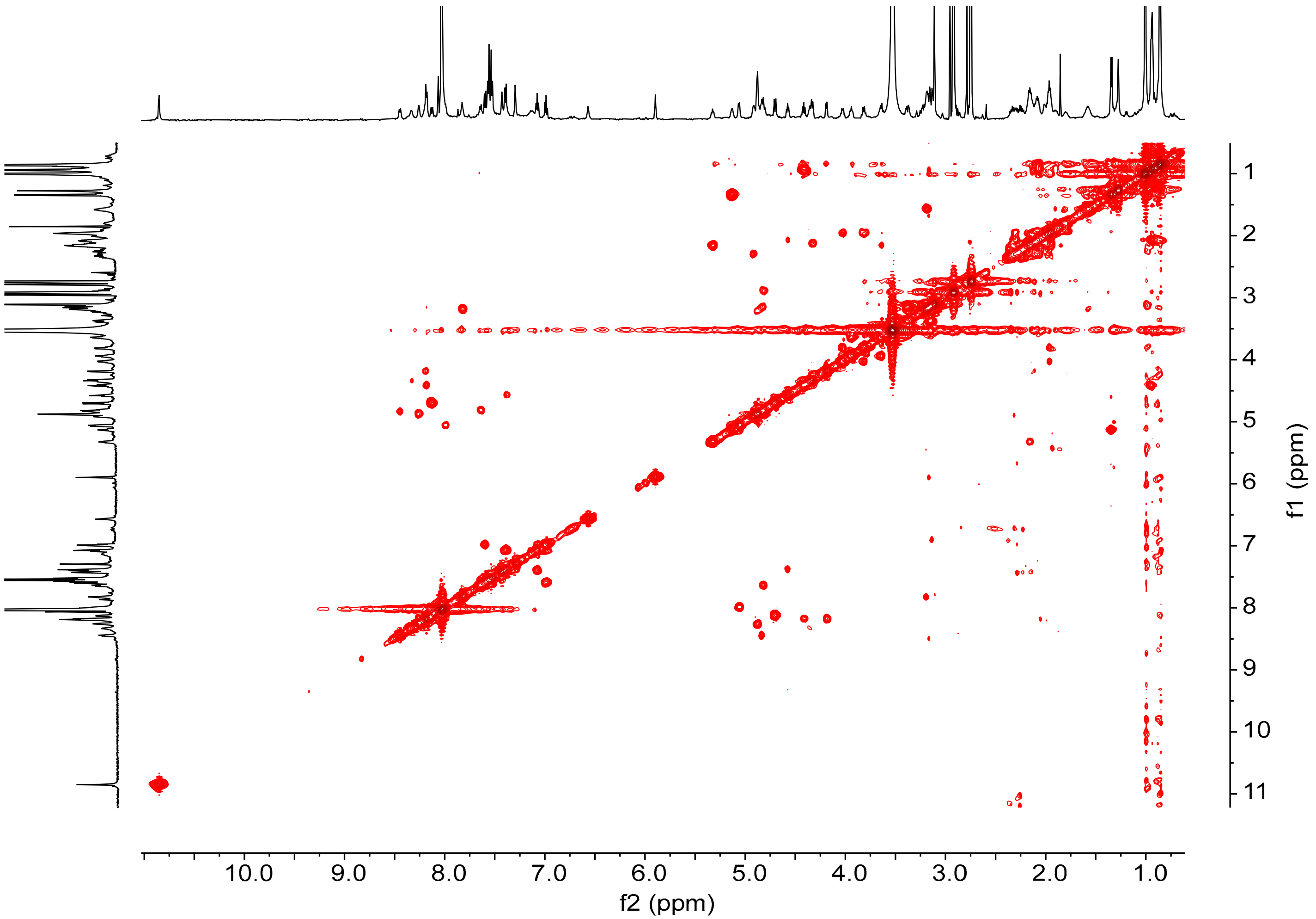

Figure S11. ^{1}H-^{1}H COSY spectrum of **1** in DMF-d_7. The spectrum was acquired over 32 scans.

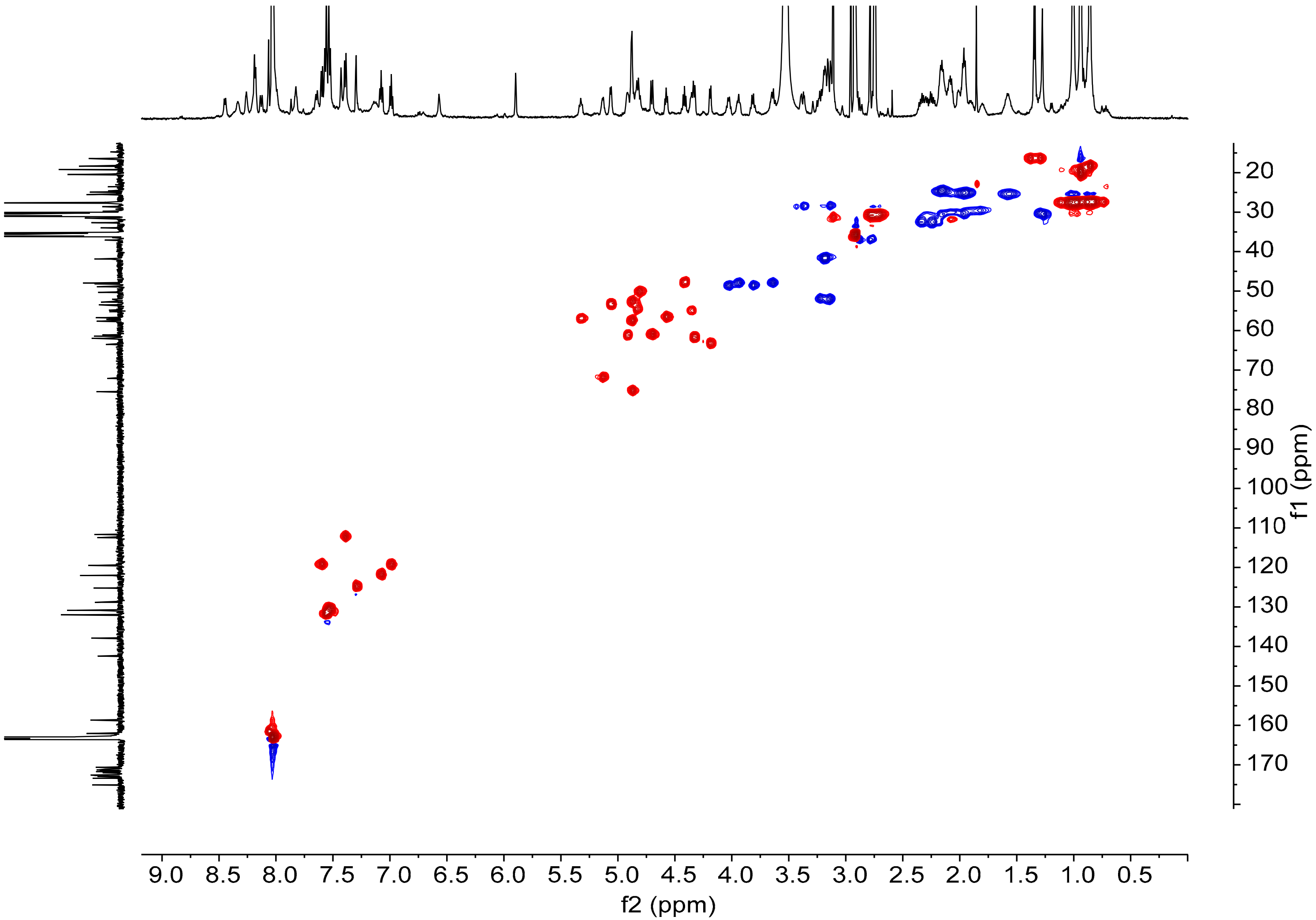

Figure S12. ^{1}H-^{13}C HSQC spectrum of **1** in DMF-d_7. The spectrum was acquired over 64 scans.

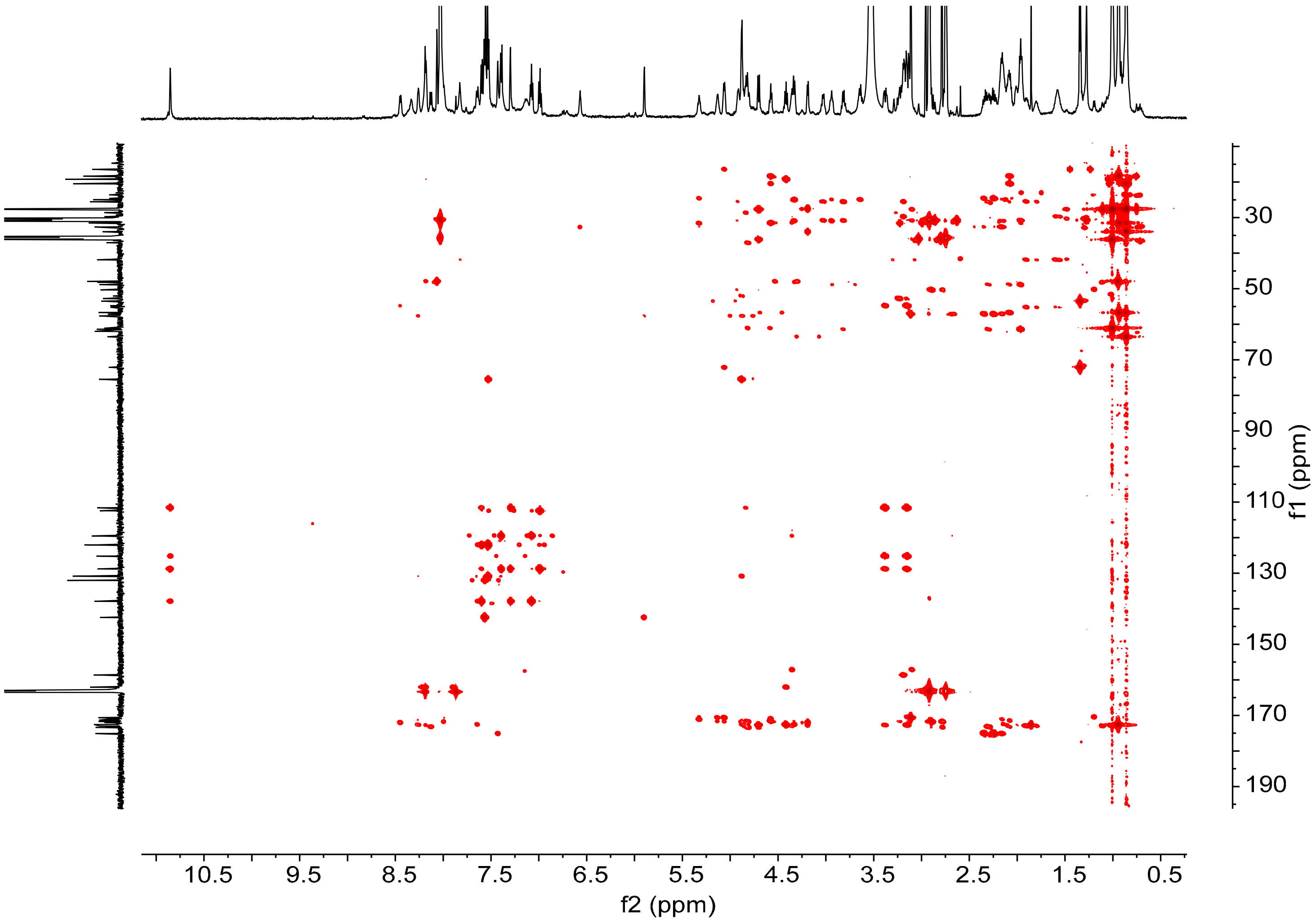

Figure S13. ^{1}H-^{13}C HMBC spectrum of **1** in DMF-d_7. The spectrum was acquired over 1760 scans.

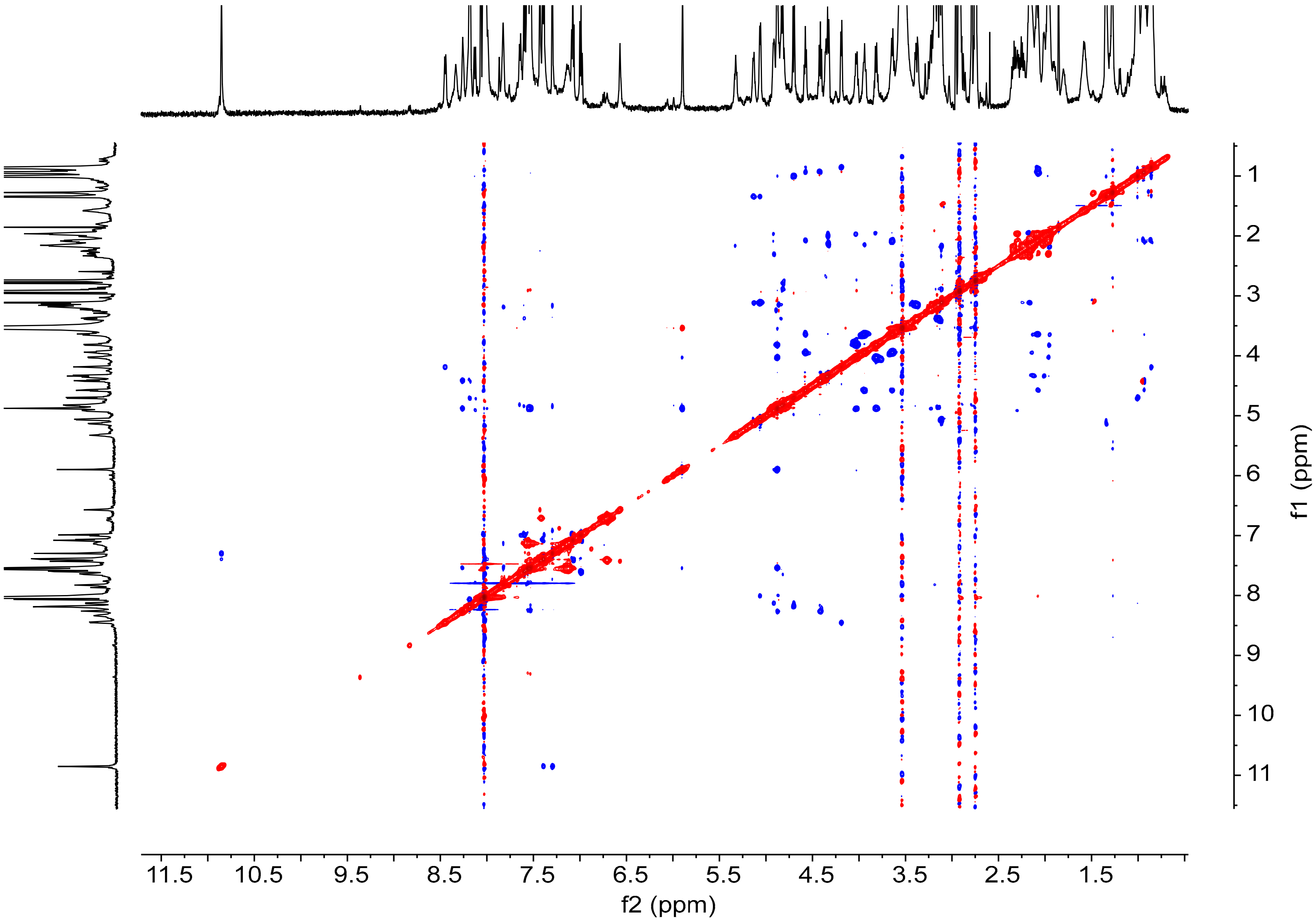

Figure S14. [1]H-^{1}H ROESY spectrum of **1** in DMF-d_7. The spectrum was acquired over 560 scans.

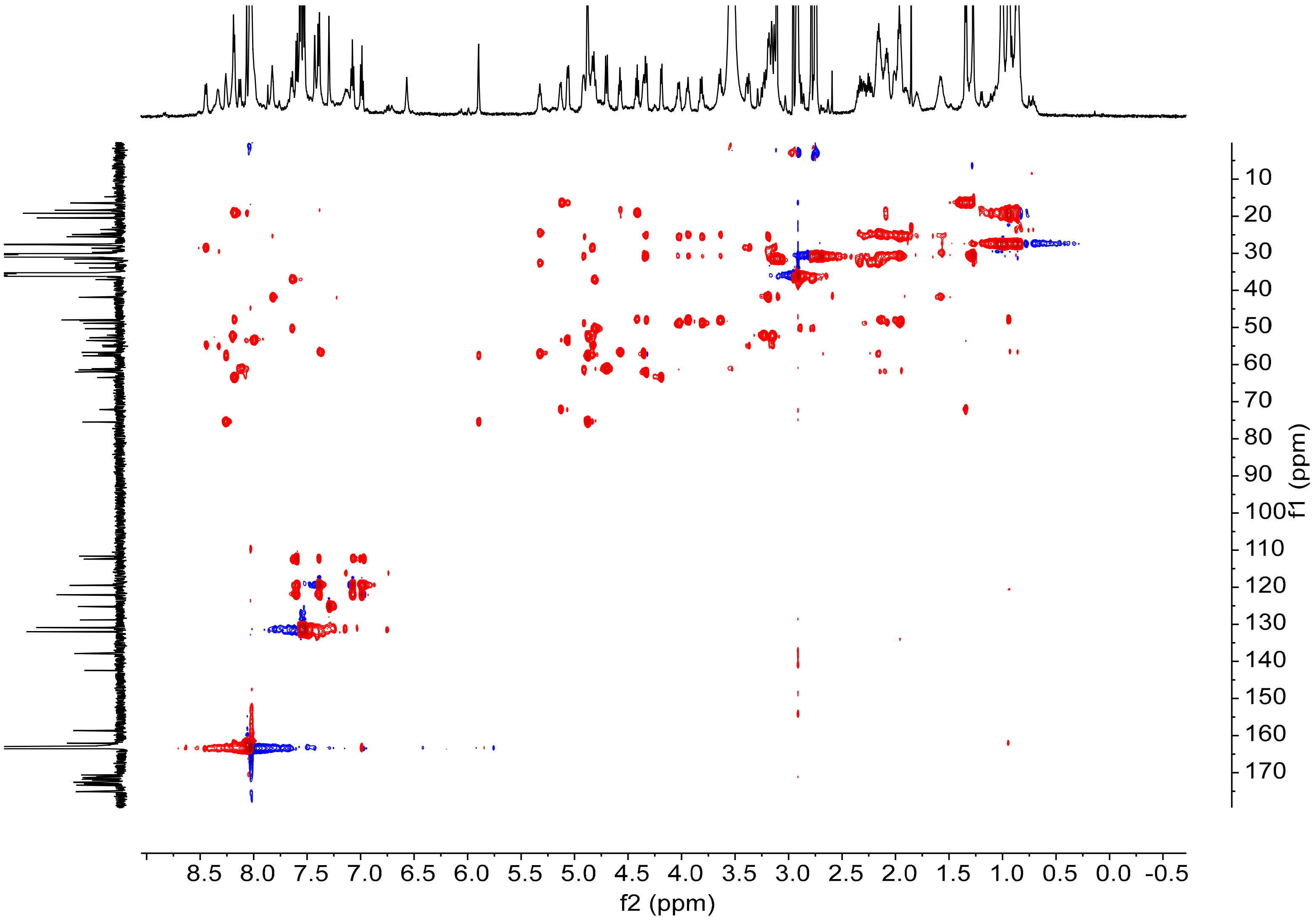

Figure S15. ^{1}H-^{13}C HSQC-TOCSY spectrum of **1** in DMF-d_7. The spectrum was acquired over 96 scans.

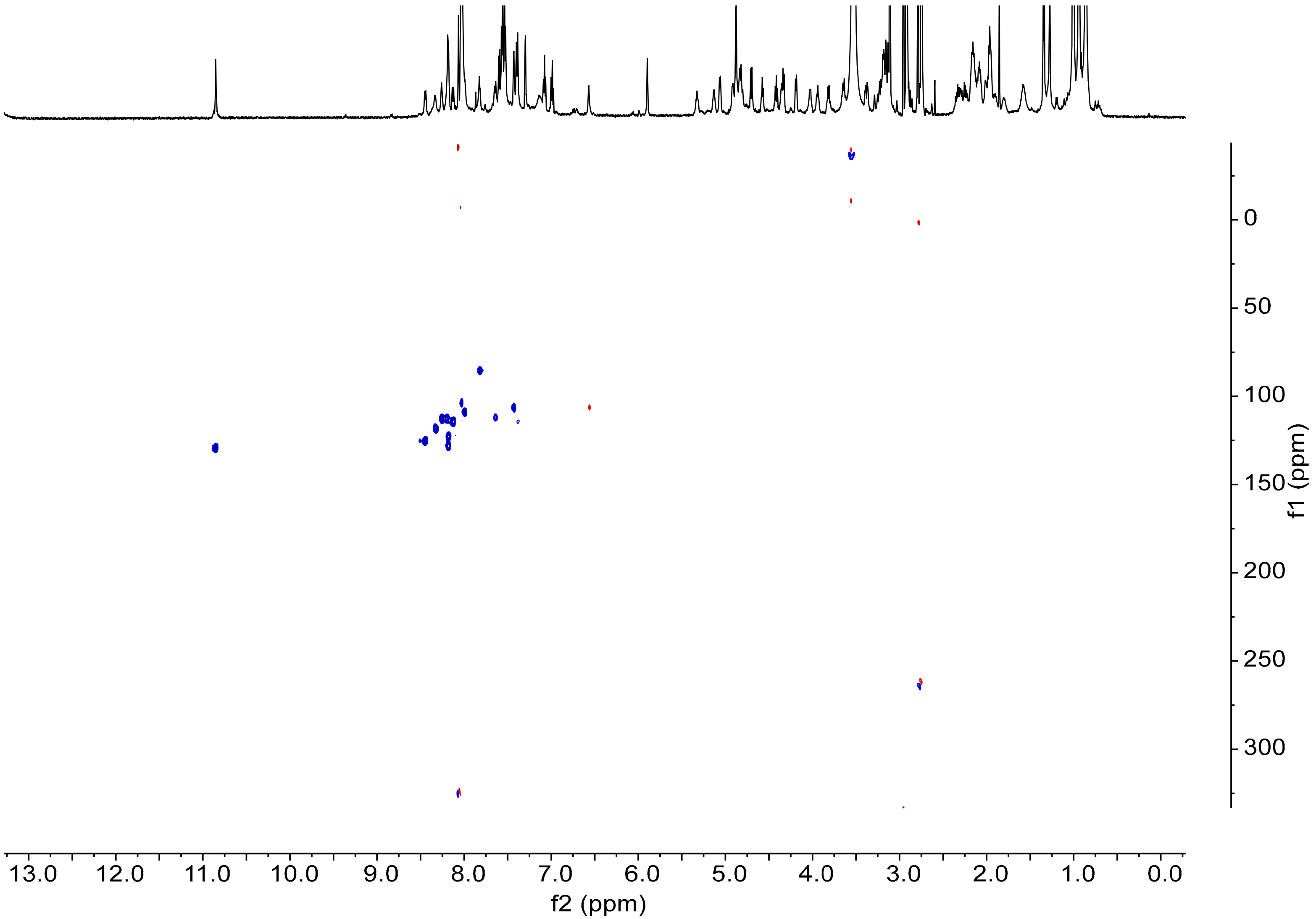

Figure S16. ^{1}H-^{15}N HSQC spectrum of **1** in DMF-d_7. The spectrum was acquired over 96 scans.

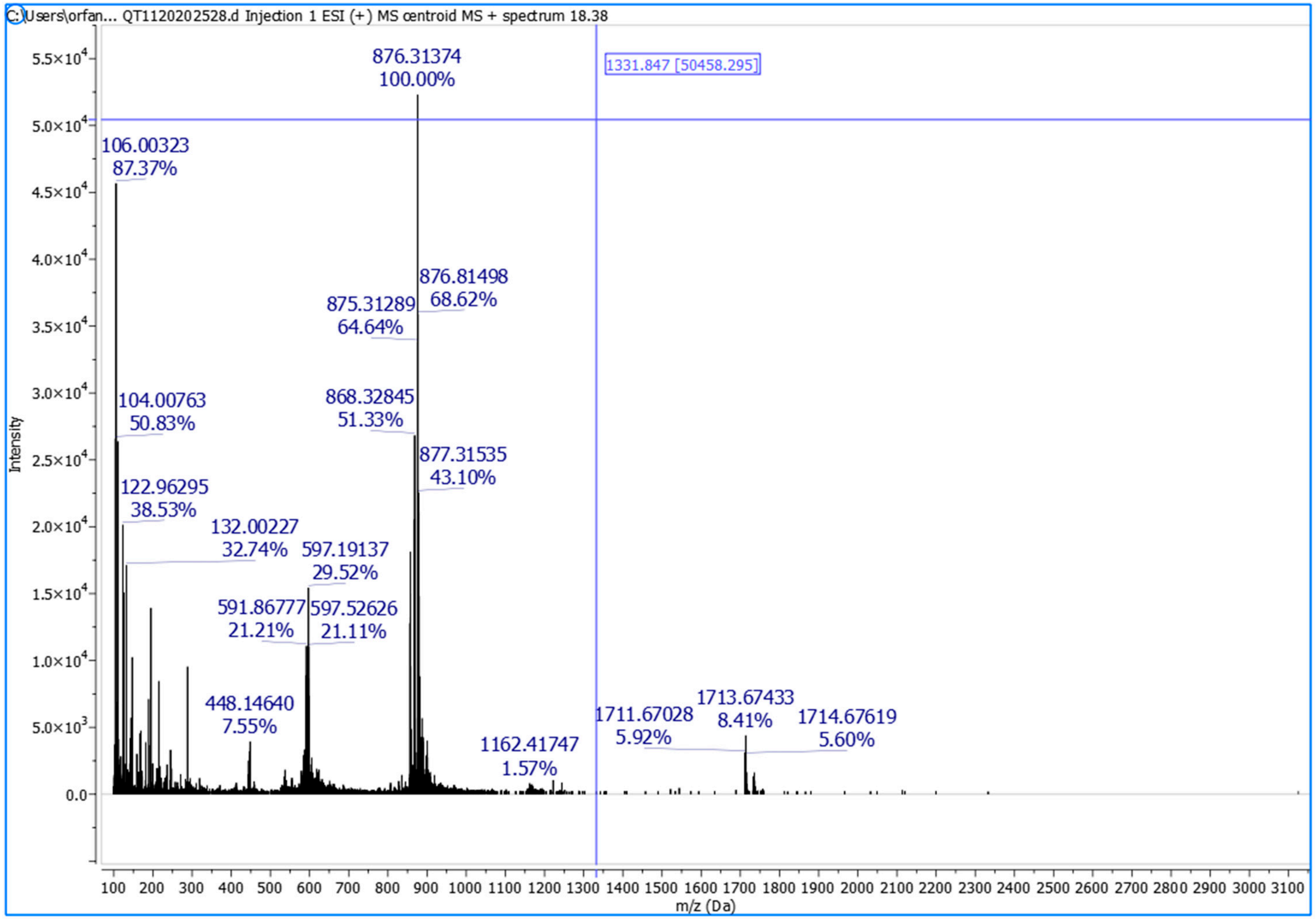

Figure S17. HRESIMS spectrum of **1**.

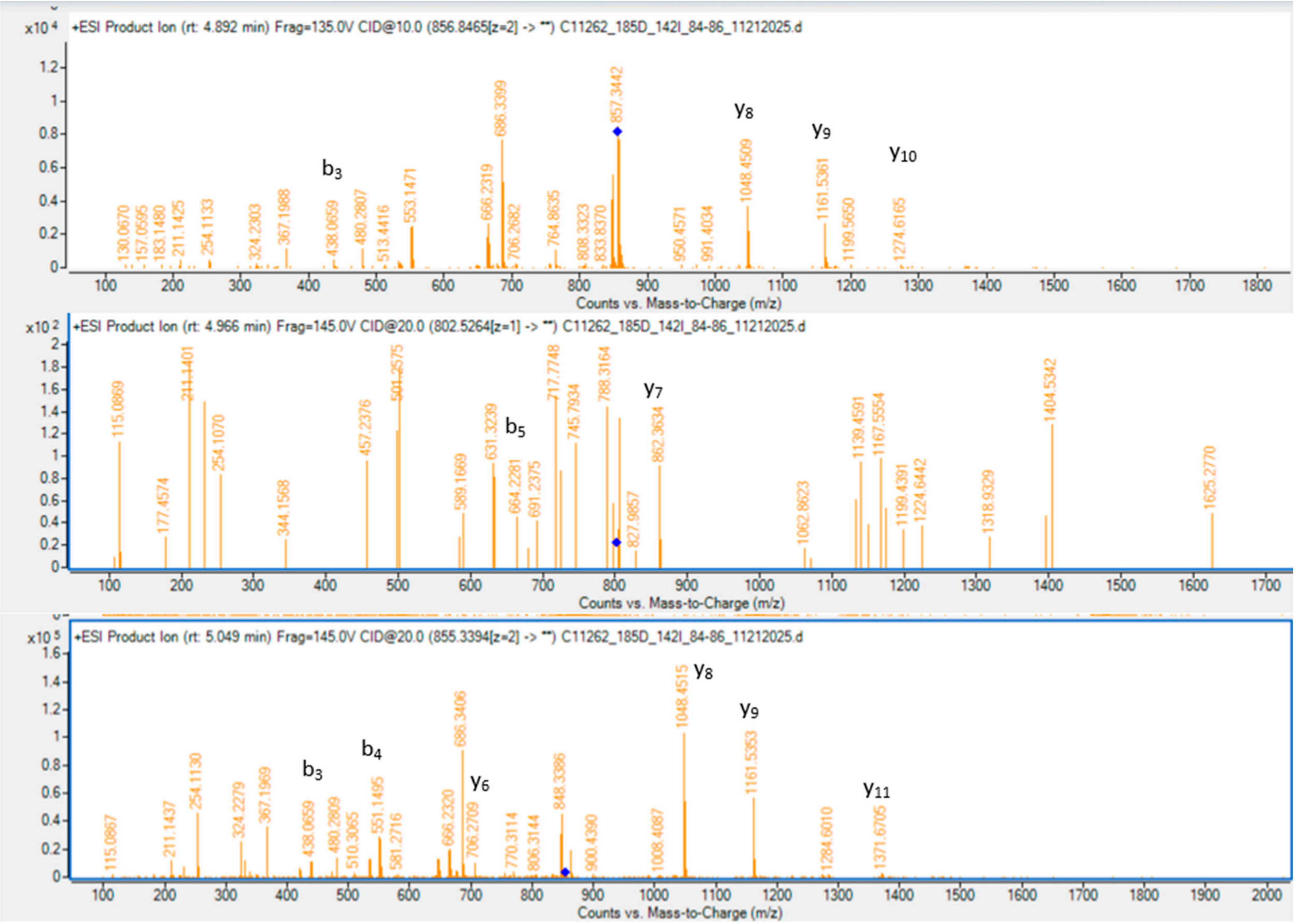

Figure S18. Positive HR-ESI-MS-MS b and y ion fragments of **1**.

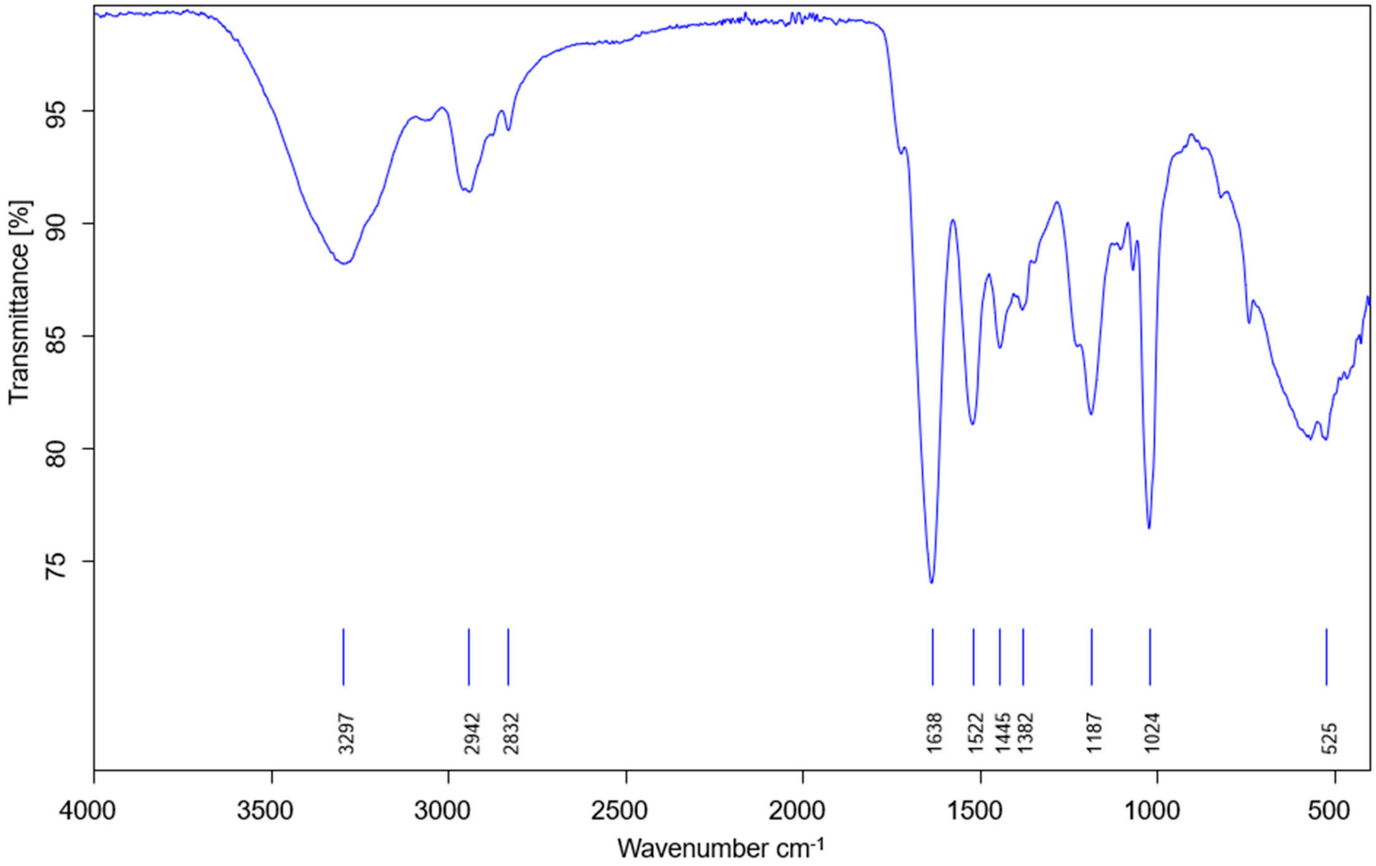

Figure S19. IR spectrum of **1**.

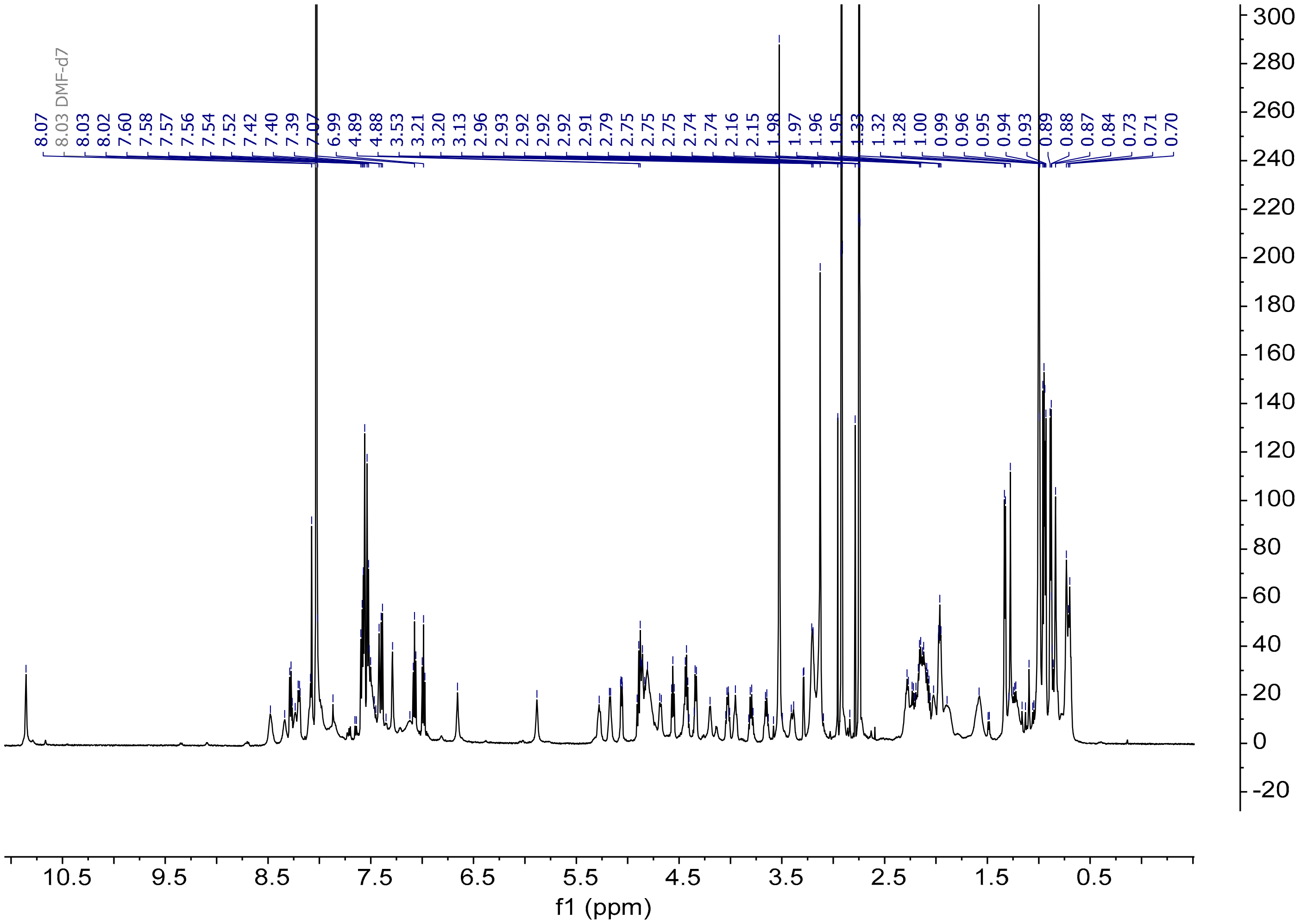

Figure S20. ^{1}H NMR spectrum (600 MHz, DMF-d_7) of **2**. The spectrum was acquired over 4 scans.

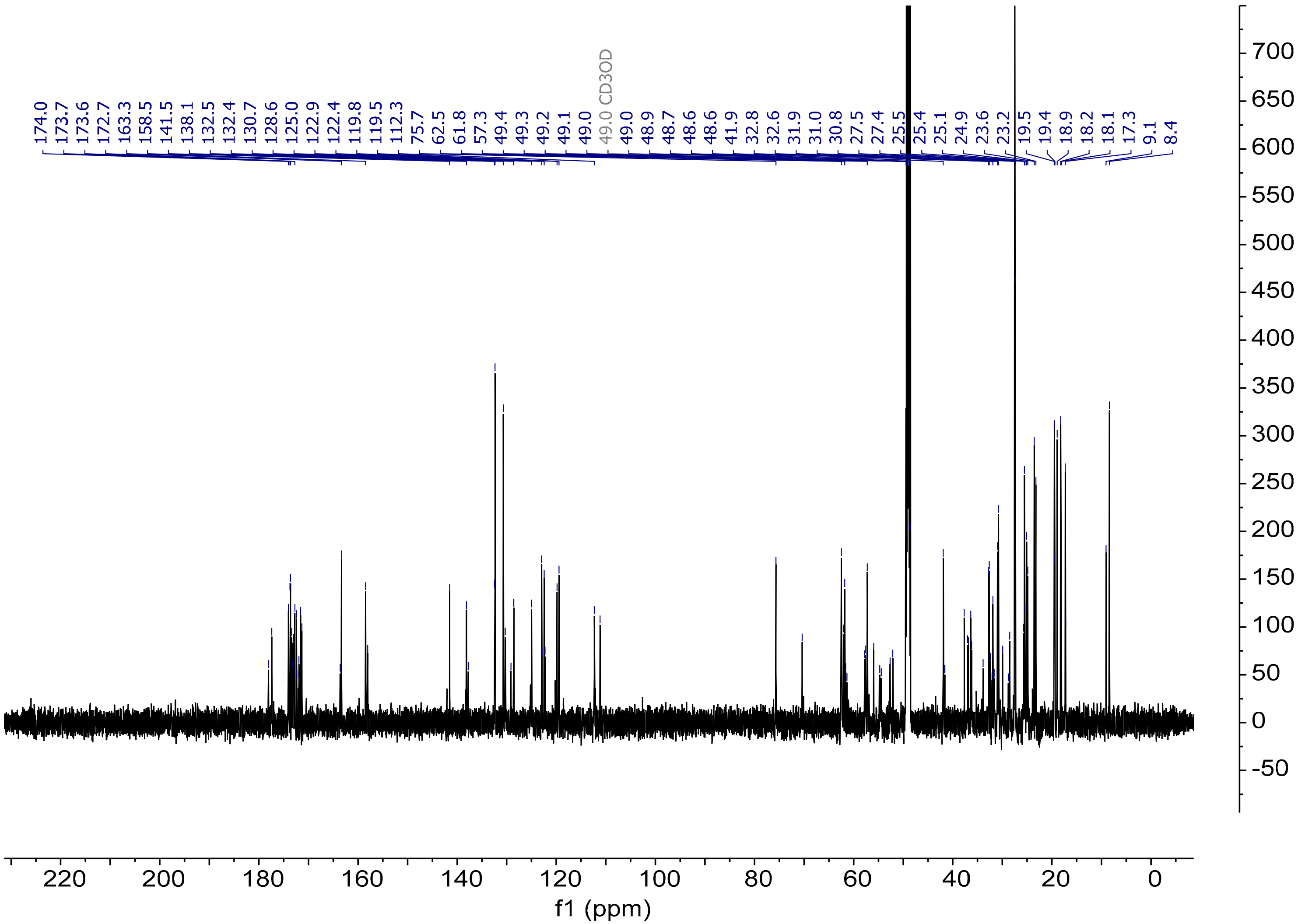

Figure S21. ^{1}H NMR spectrum (600 MHz, MeOD-d_4) of **2**. The spectrum was acquired over 4 scans.

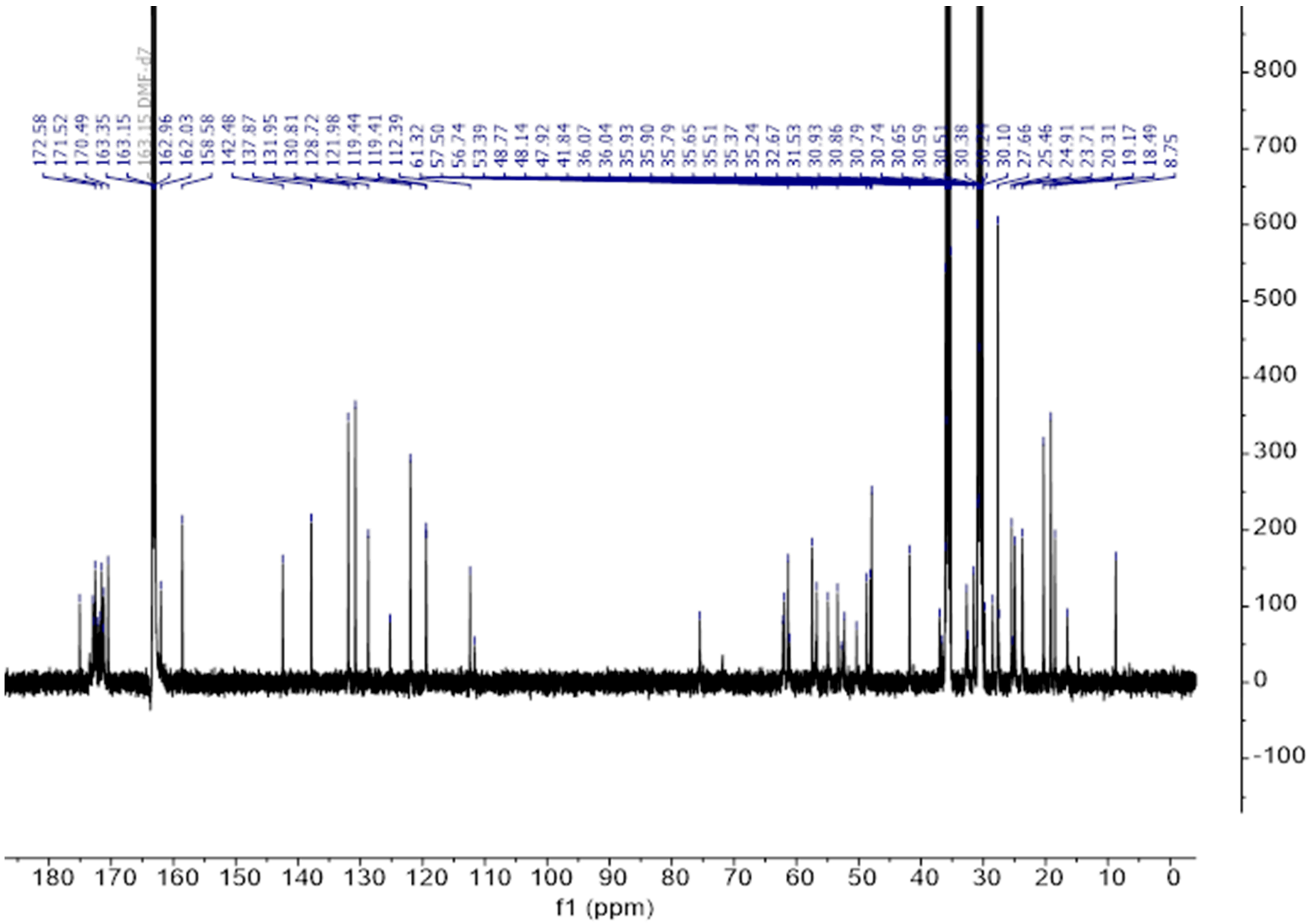

Figure S22. ^{13}C NMR spectrum (150 MHz, DMF-d_7) of **2**. The spectrum was acquired over 4000 scans.

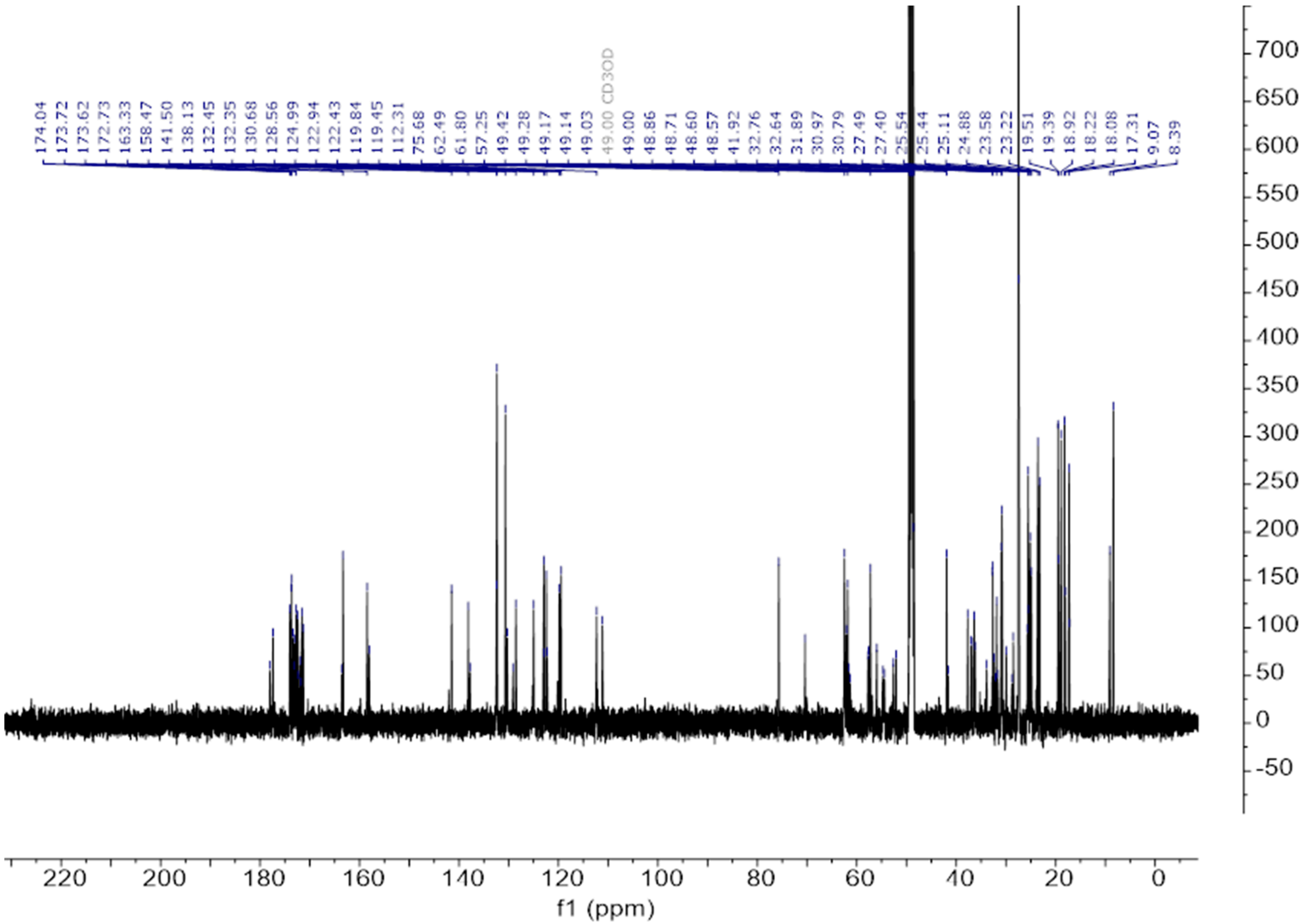

Figure S23. ^{13}C NMR spectrum (150 MHz, MeOD-d_4) of **2**. The spectrum was acquired over 4000 scans.

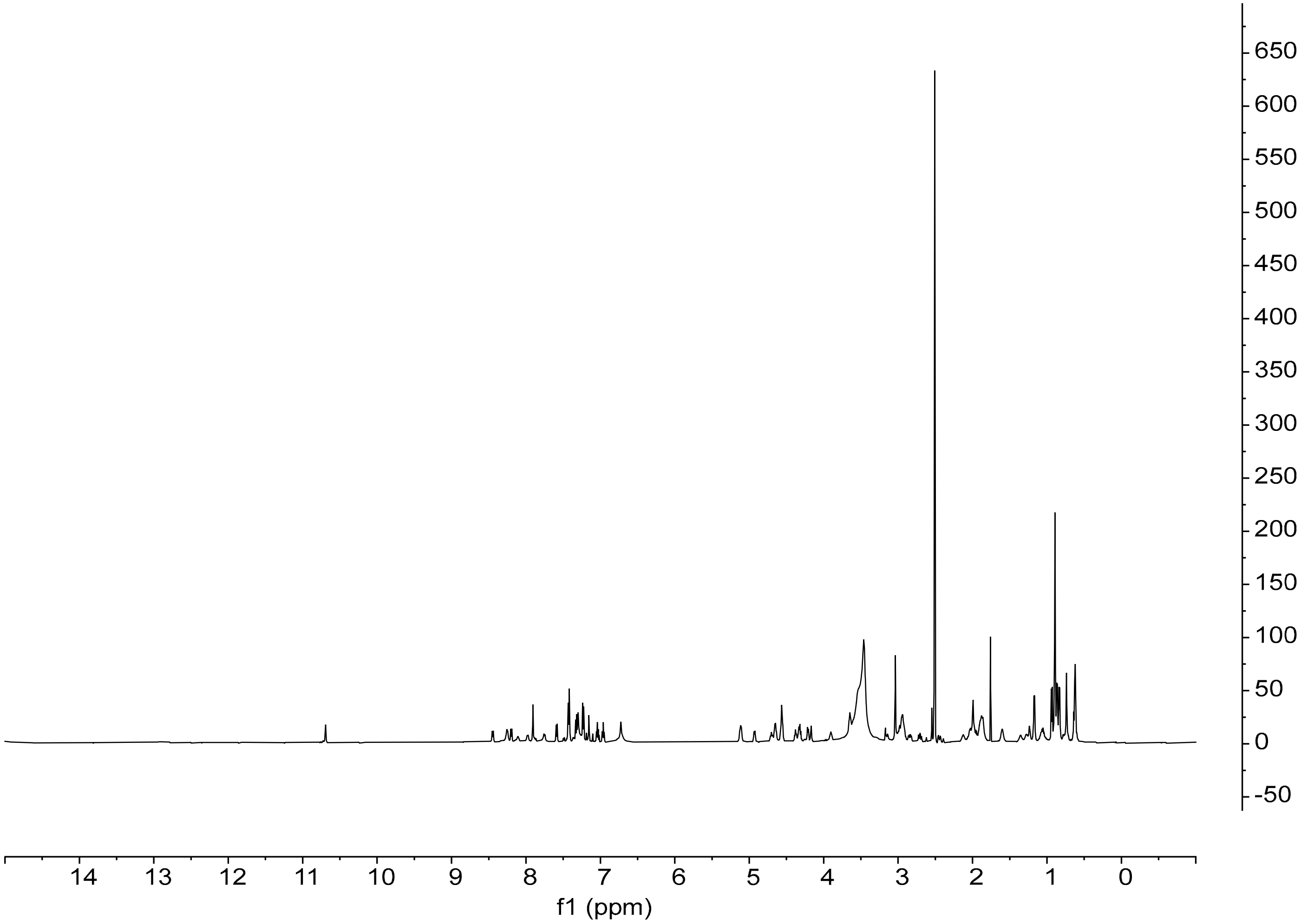

Figure S24. ^{1}H NMR spectrum (600 MHz, DMSO-d_6) of **3**. The spectrum was acquired over 4 scans.

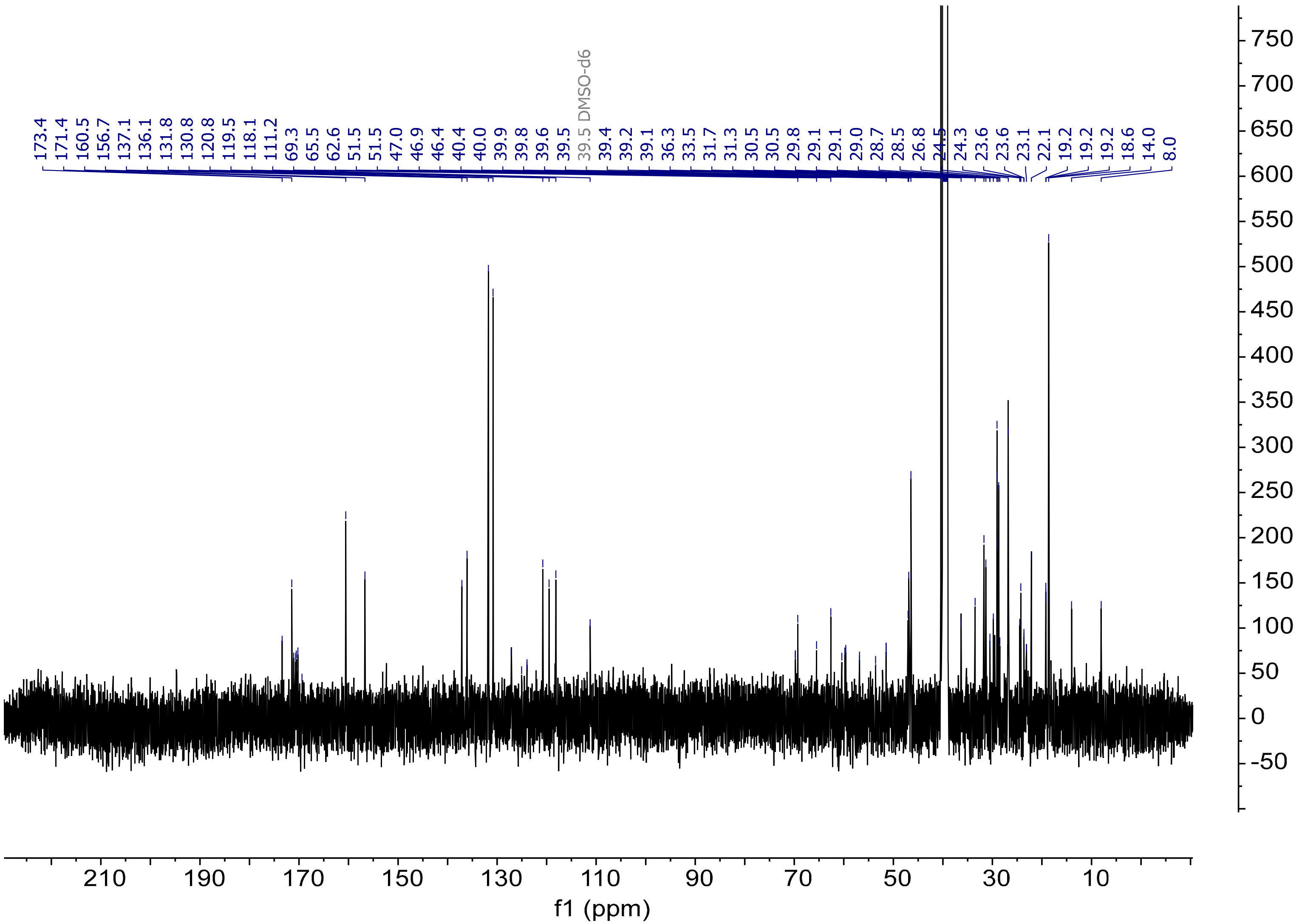

Figure S25. ^{13}C NMR spectrum (150 MHz, DMSO-d_6) of **3**. The spectrum was acquired over 30000 scans.

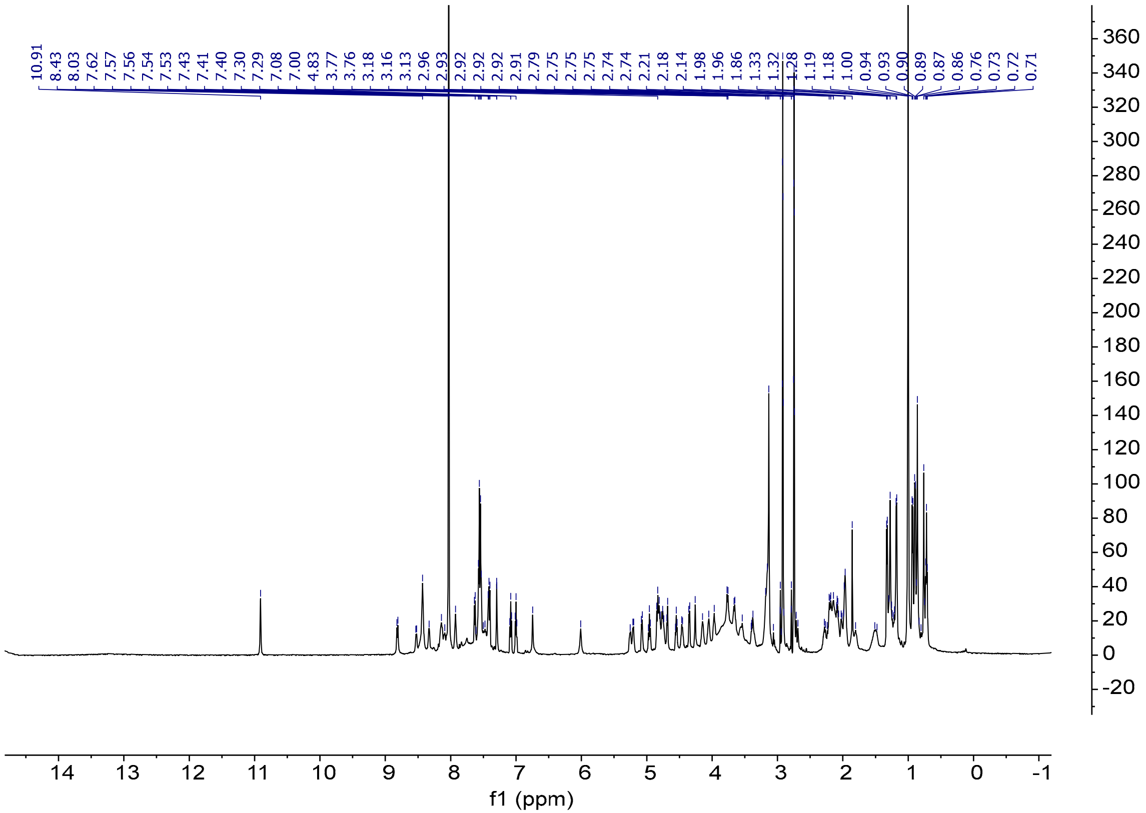

Figure S26. ^{1}H NMR spectrum (600 MHz, DMF-d_7) of **4**. The spectrum was acquired over 4 scans.

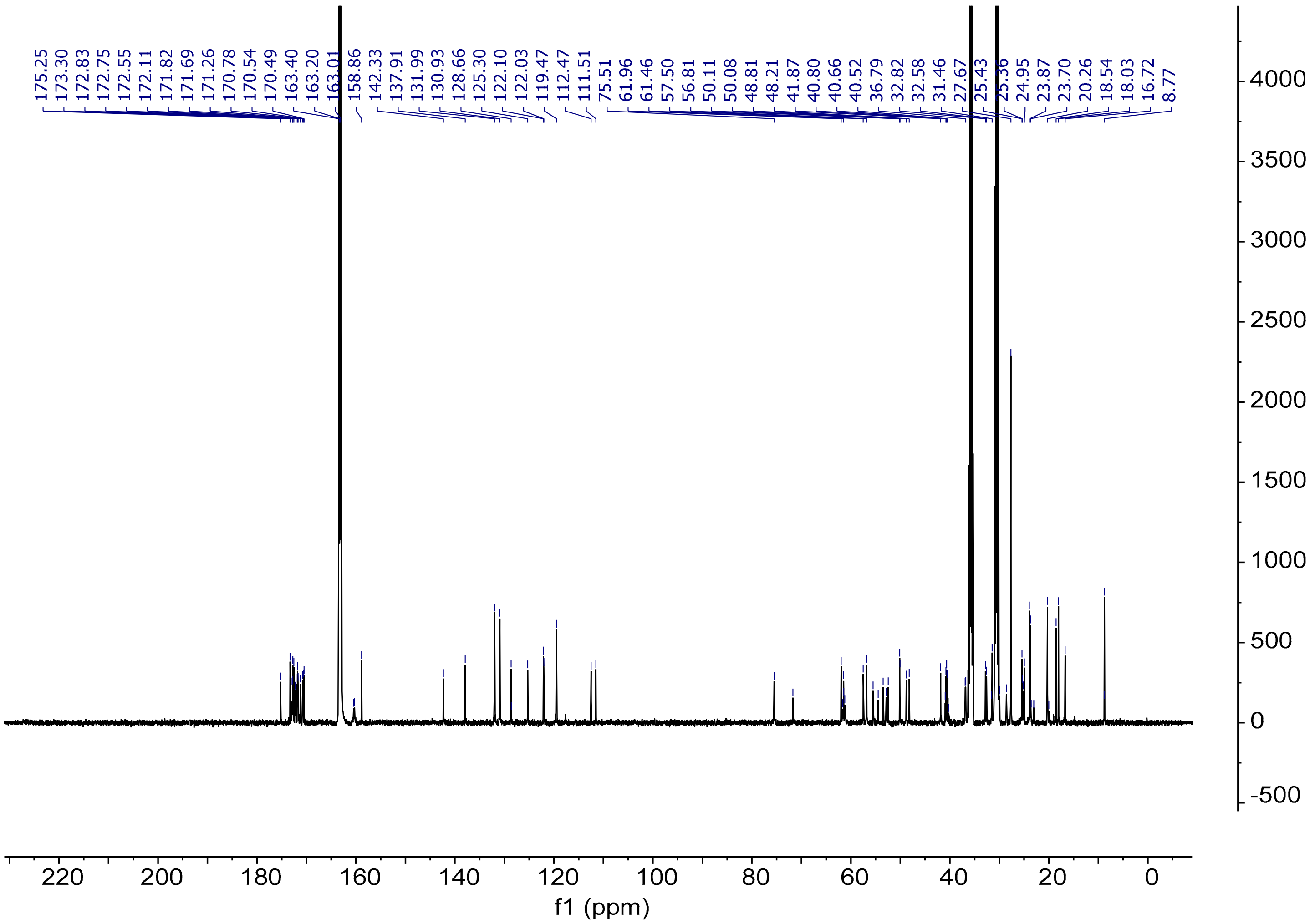

Figure S27. ^{13}C NMR spectrum (150 MHz, DMF-d_7) of **4**. The spectrum was acquired over 12000 scans.

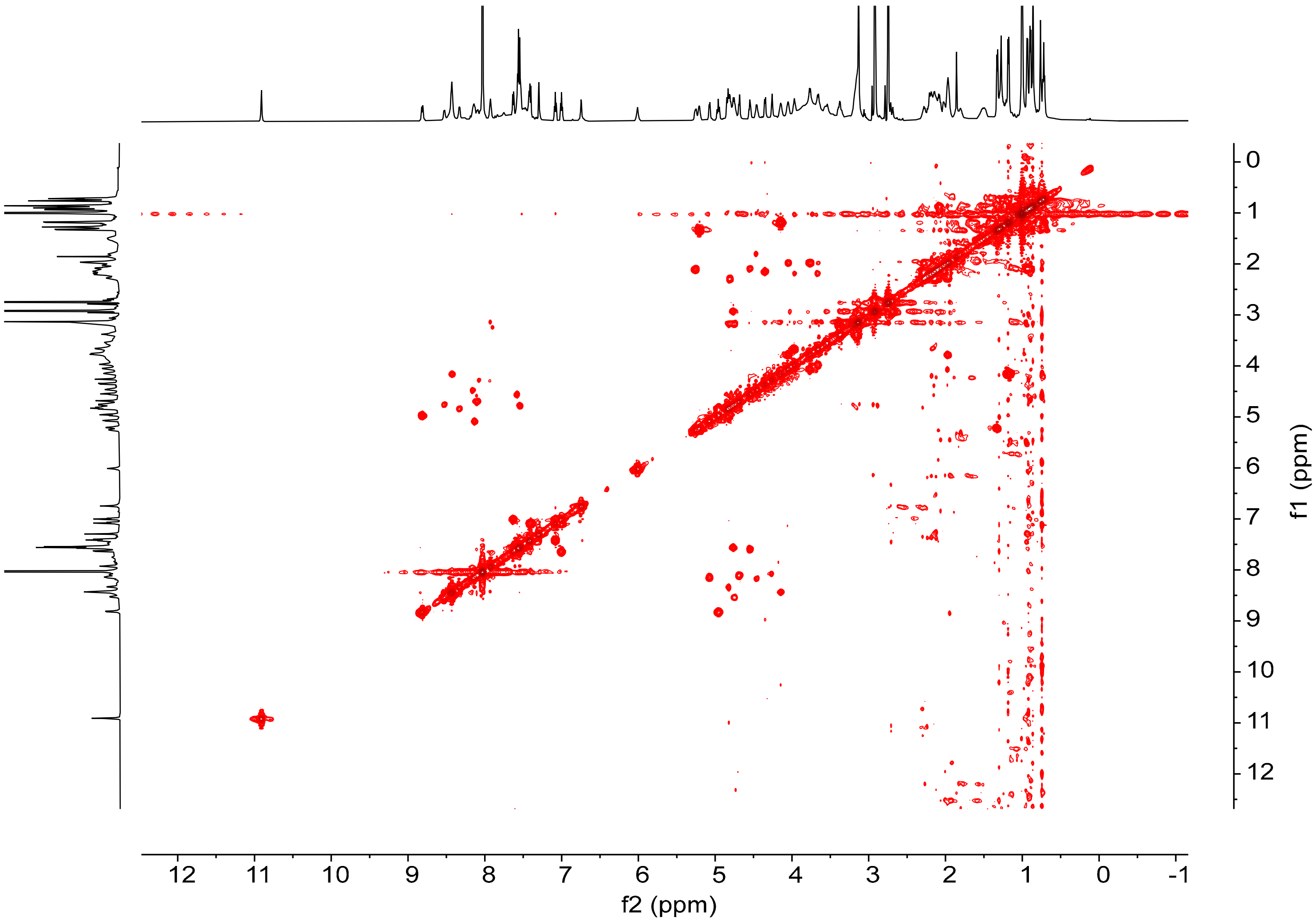

Figure S28. ^1H-^1H COSY spectrum of **4** in DMF-d_7. The spectrum was acquired over 16 scans.

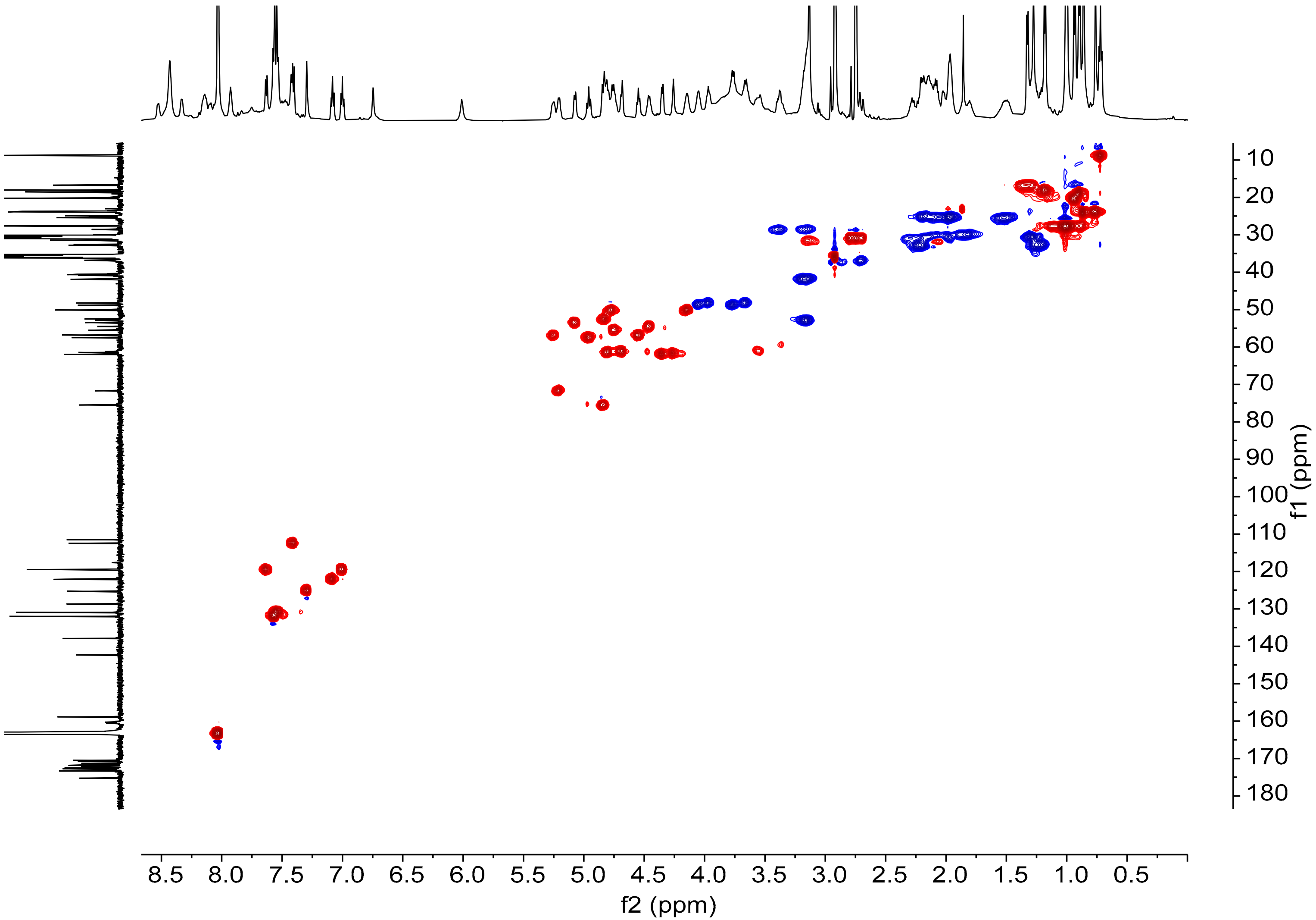

Figure S29. ^{1}H-^{13}C HSQC spectrum of **4** in DMF-d_7. The spectrum was acquired over 32 scans.

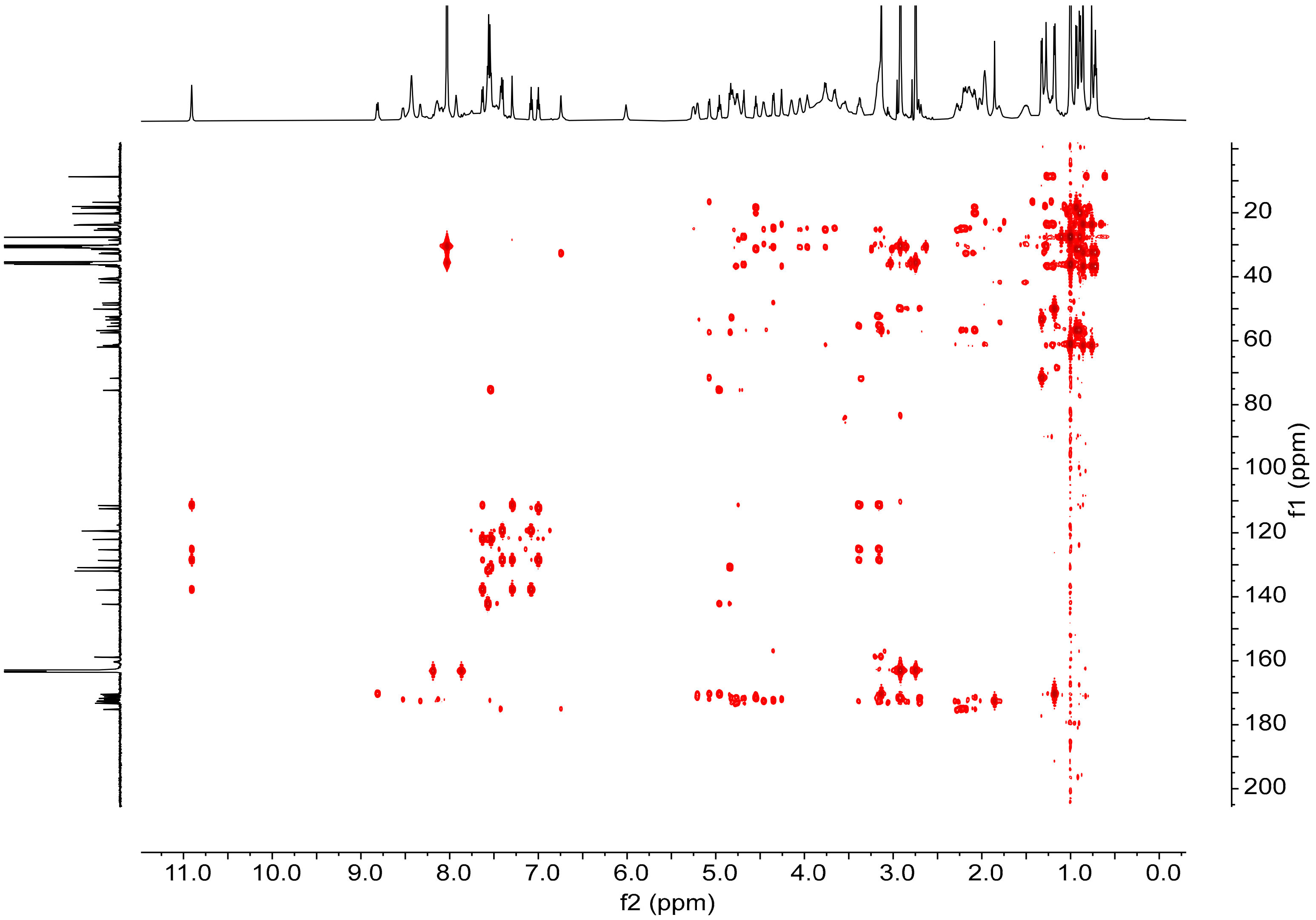

Figure S30. ^{1}H-^{13}C HMBC spectrum of **4** in DMF-d_7. The spectrum was acquired over 560 scans.

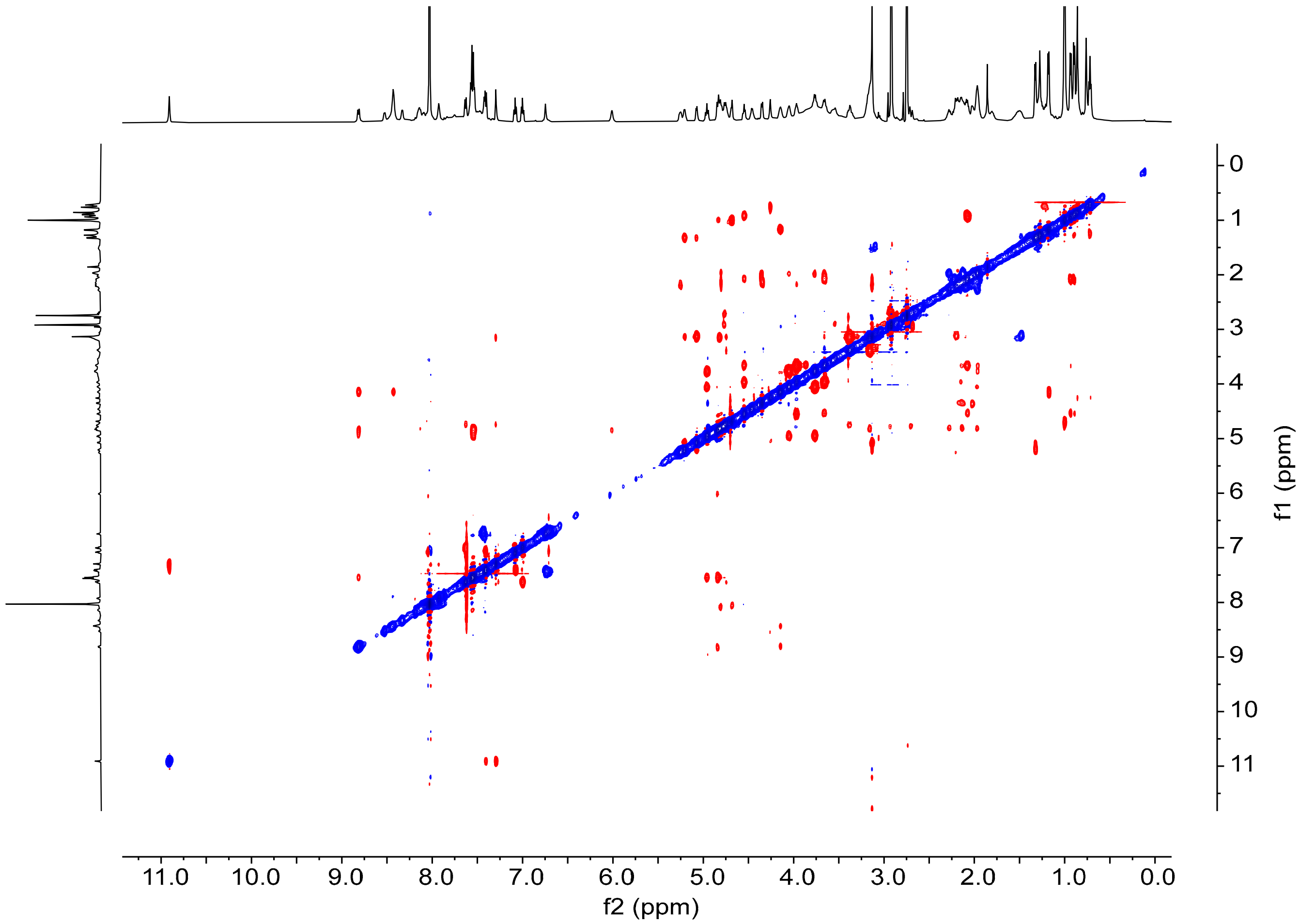

Figure S31. ¹H-¹H ROESY spectrum of **4** in DMF-d_7. The spectrum was acquired over 128 scans.

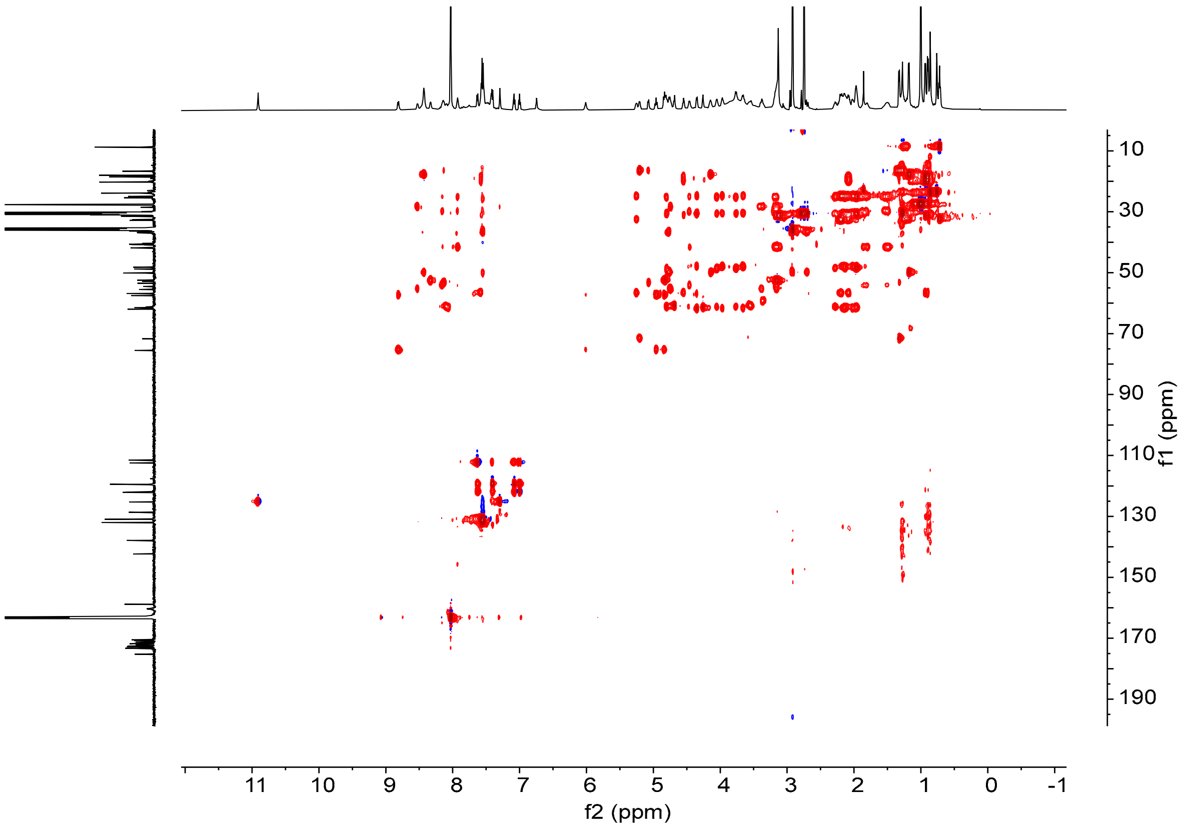

Figure S32. ^{1}H-^{13}C HSQC-TOCSY spectrum of **4** in DMF-d_7. The spectrum was acquired over 48 scans.

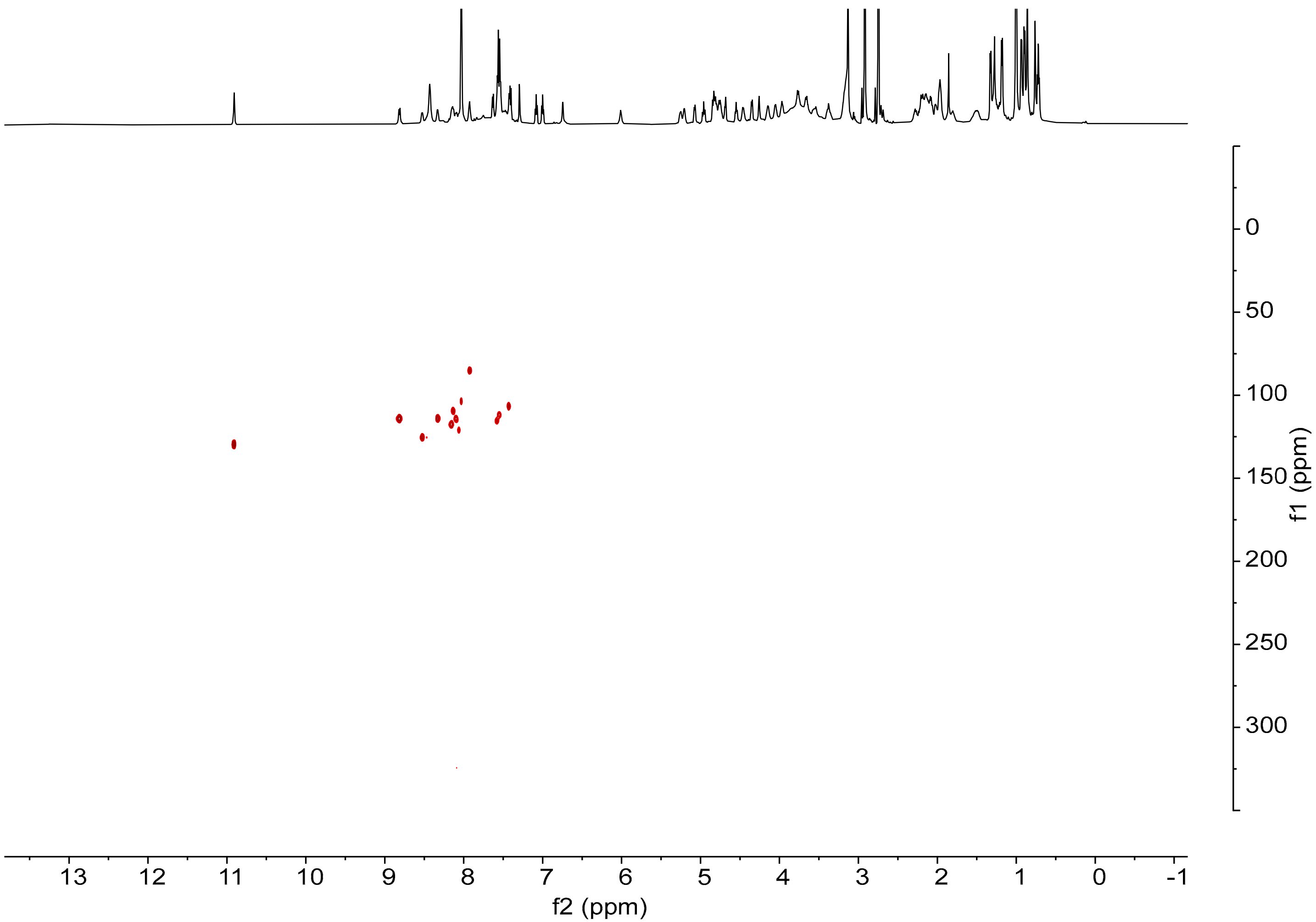

Figure S33. ^1H-^{15}N HSQC spectrum of **4** in DMF-d_7. The spectrum was acquired over 48 scans.

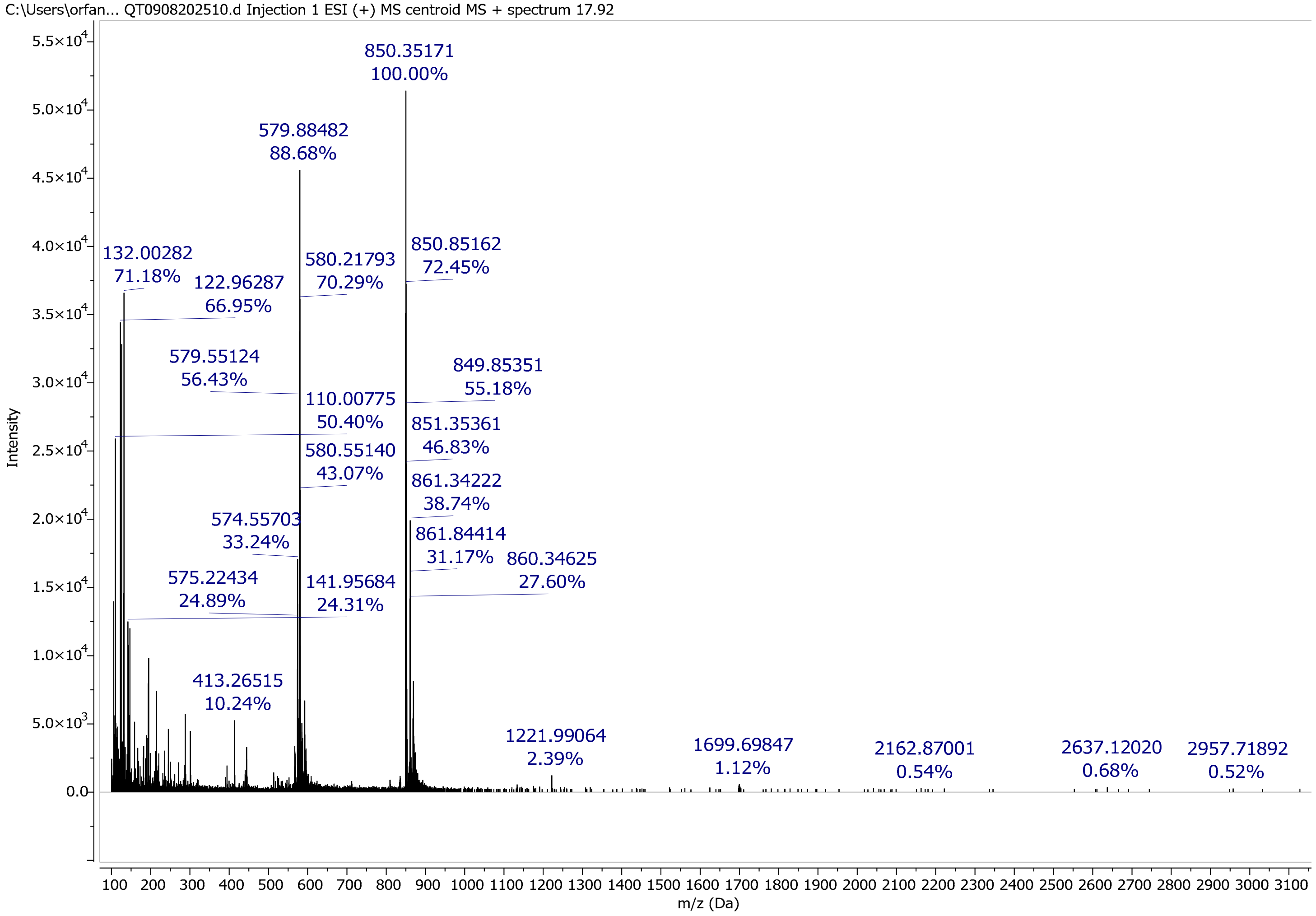

Figure S34. HRESIMS spectrum of **4**.

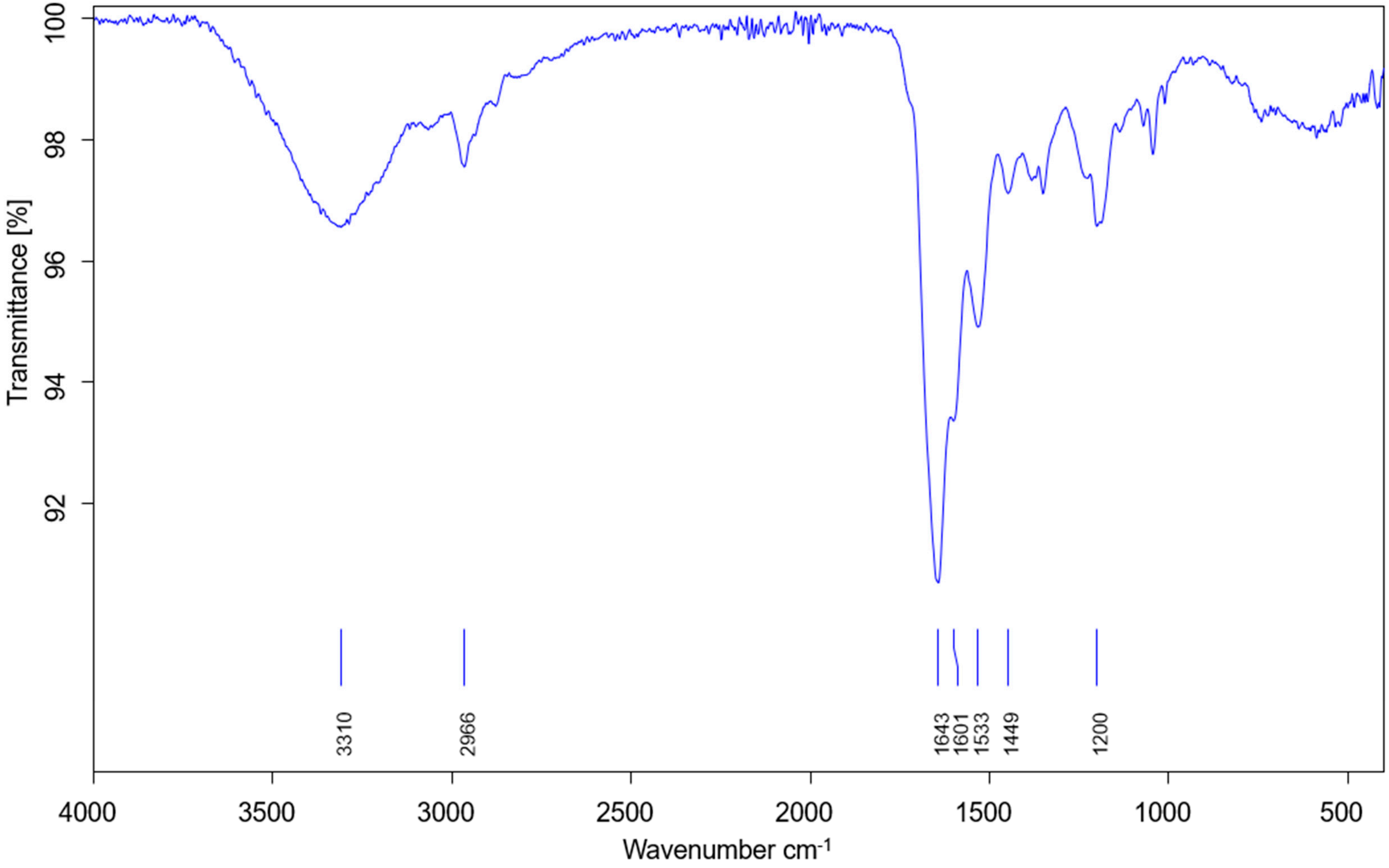

Figure S35. IR spectrum of **4**.

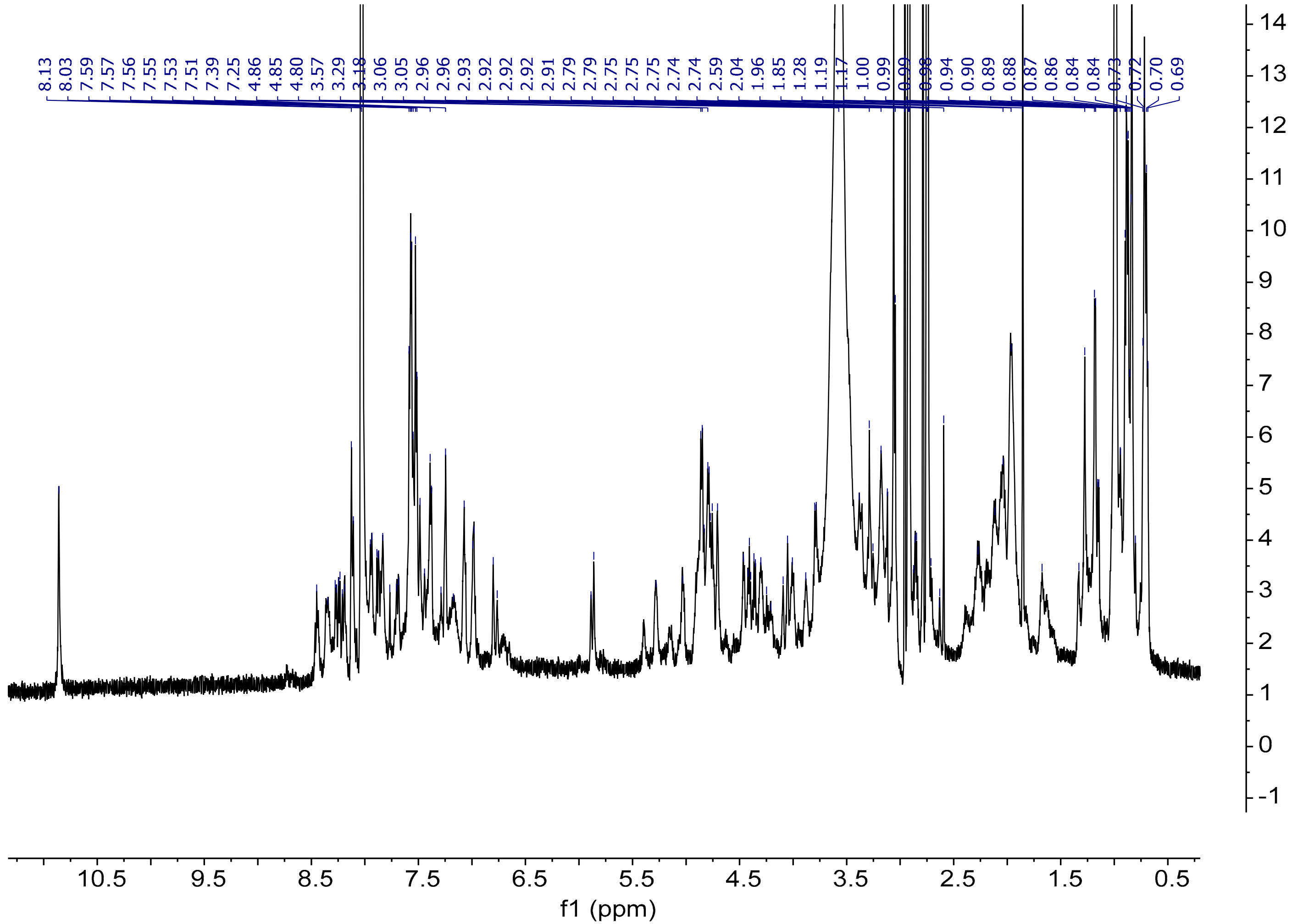

Figure S36. ^{1}H NMR spectrum (600 MHz, DMF-d_7) of **5**. The spectrum was acquired over 4 scans.

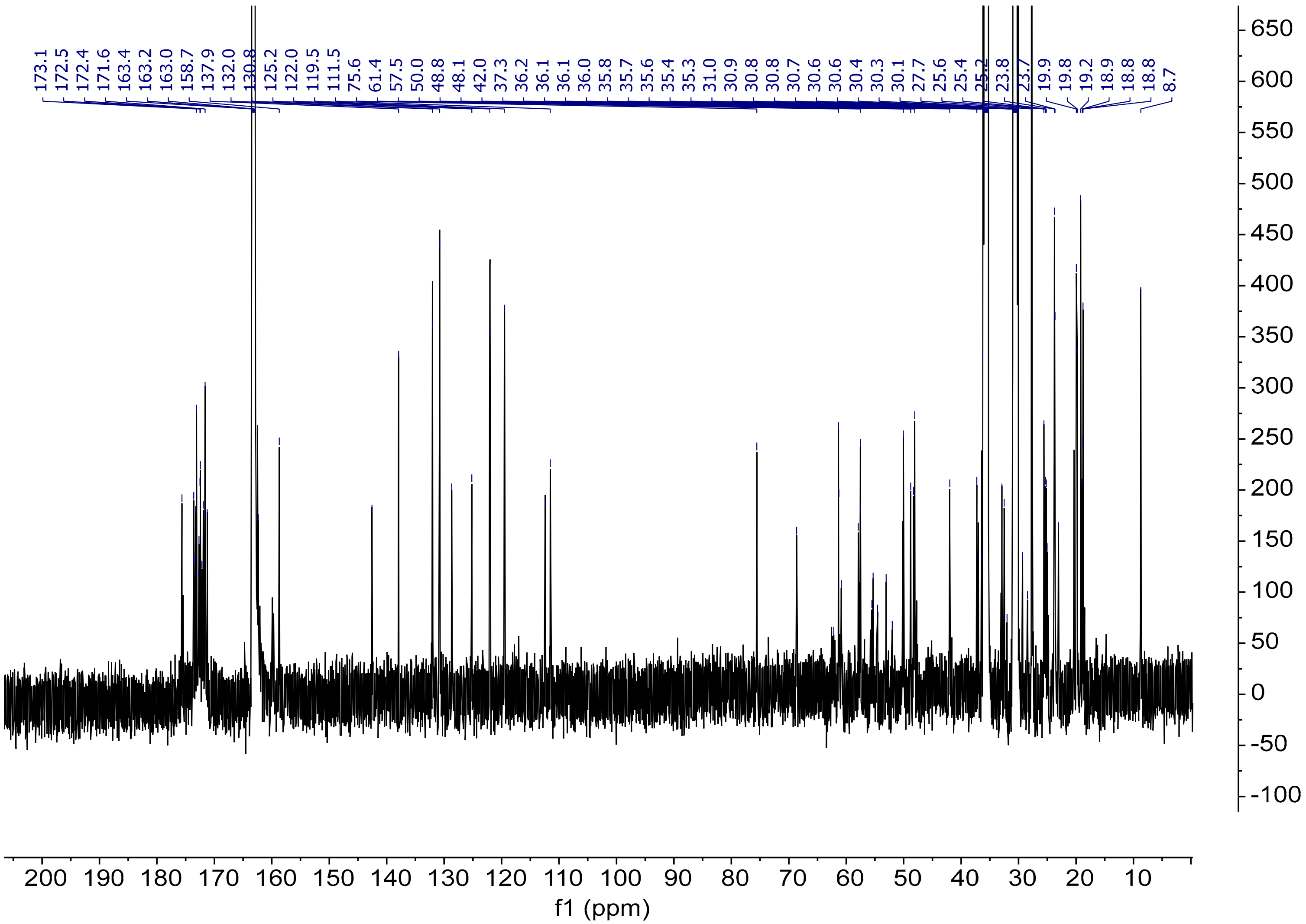

Figure S37. ^{13}C NMR spectrum (150 MHz, DMF-d_7) of **5**. The spectrum was acquired over 35000 scans.

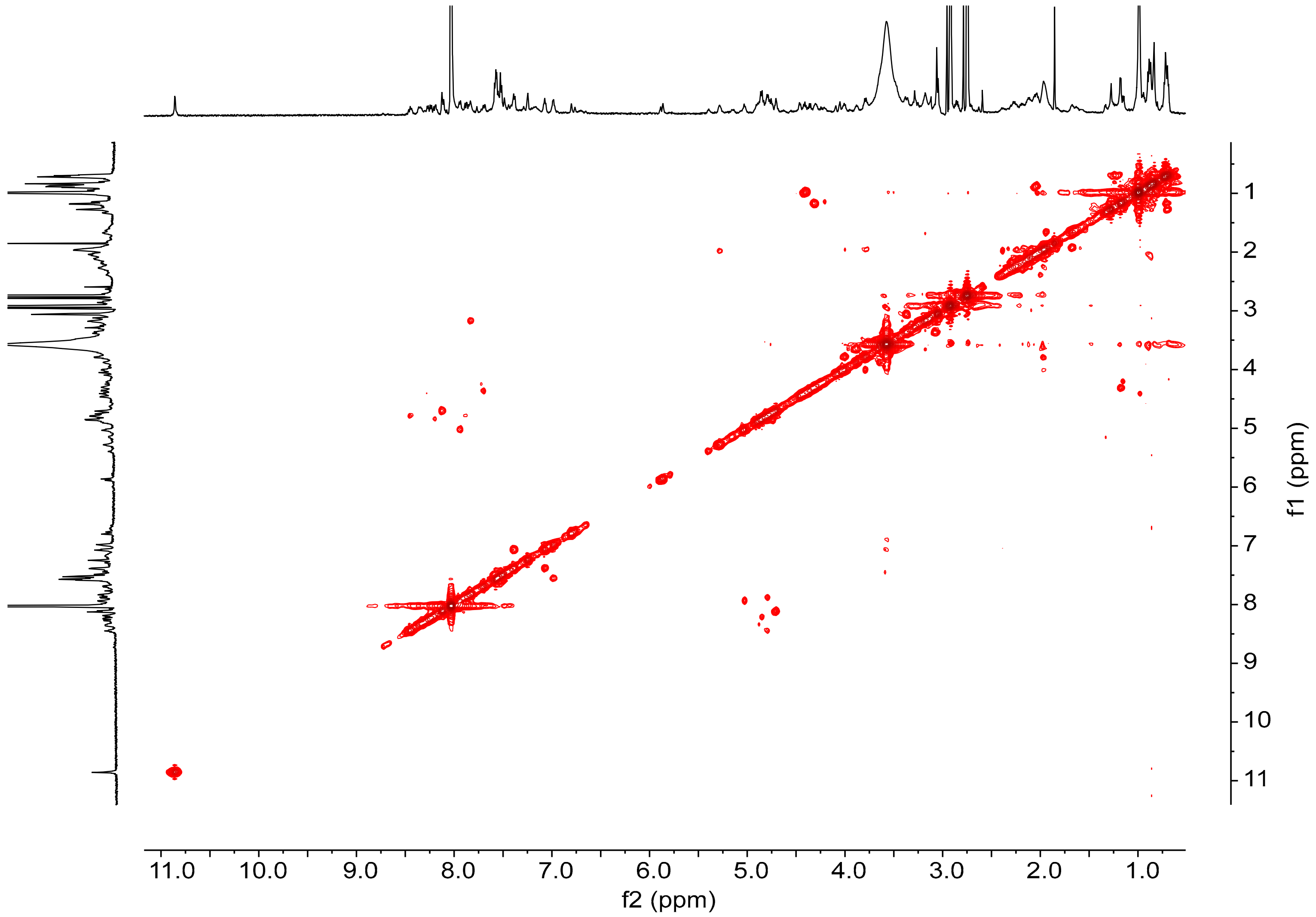

Figure S38. ^{1}H-^{1}H COSY spectrum of **5** in DMF-d_7. The spectrum was acquired over 200 scans.

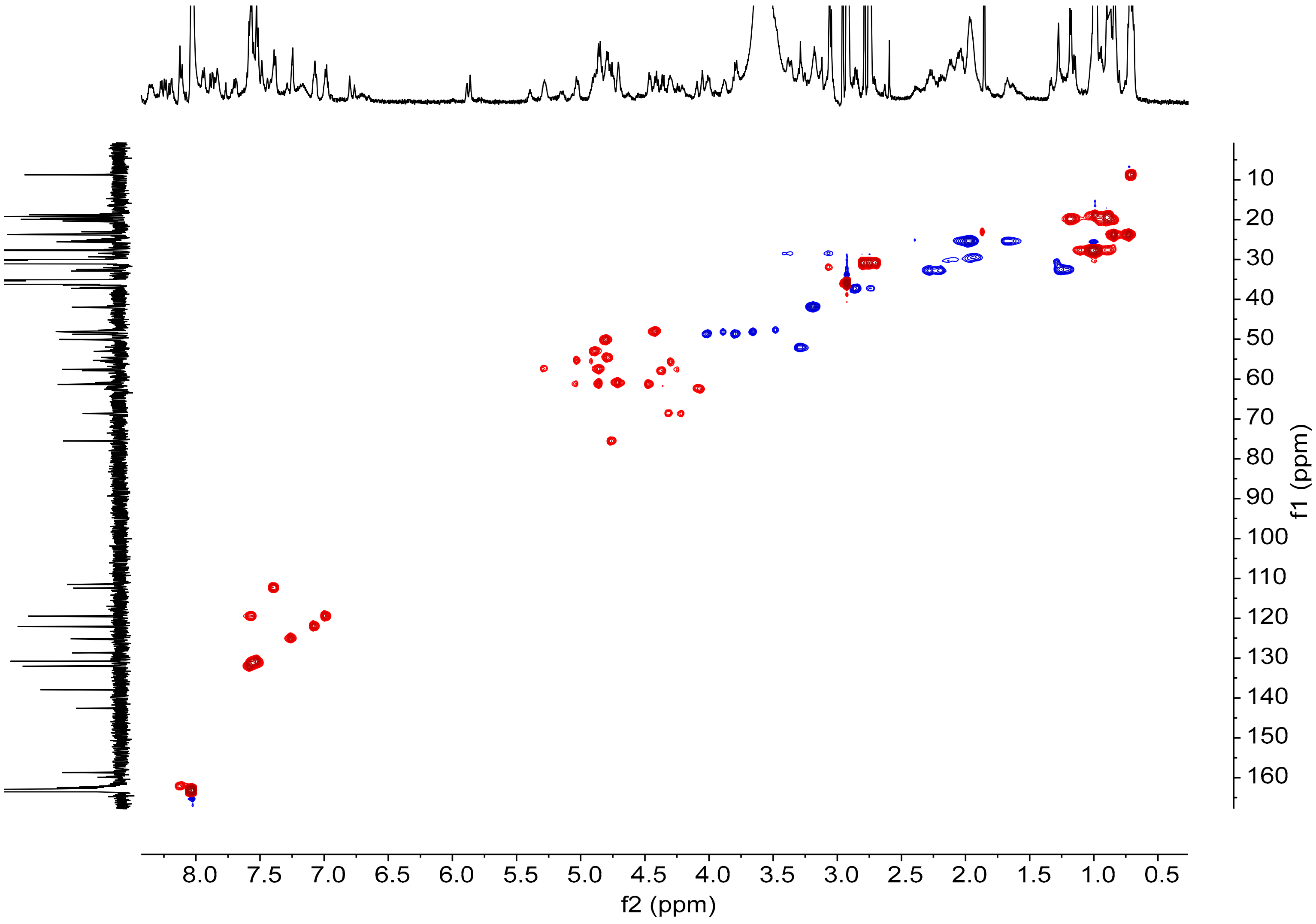

Figure S39. ^{1}H-^{13}C HSQC spectrum of **5** in DMF-d_7. The spectrum was acquired over 64 scans.

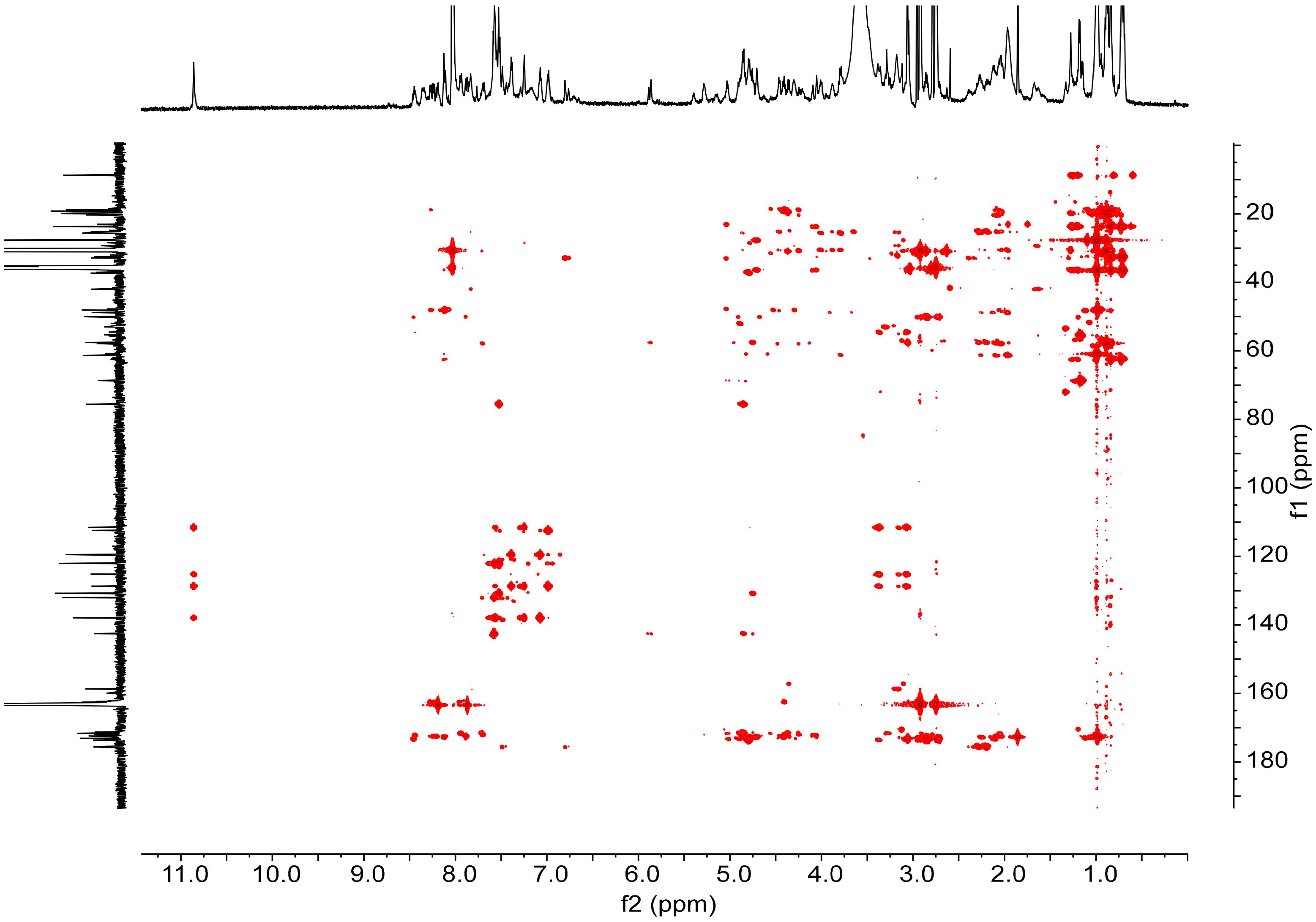

Figure S40. ^{1}H-^{13}C HMBC spectrum of **5** in DMF-d_7. The spectrum was acquired over 1360 scans.

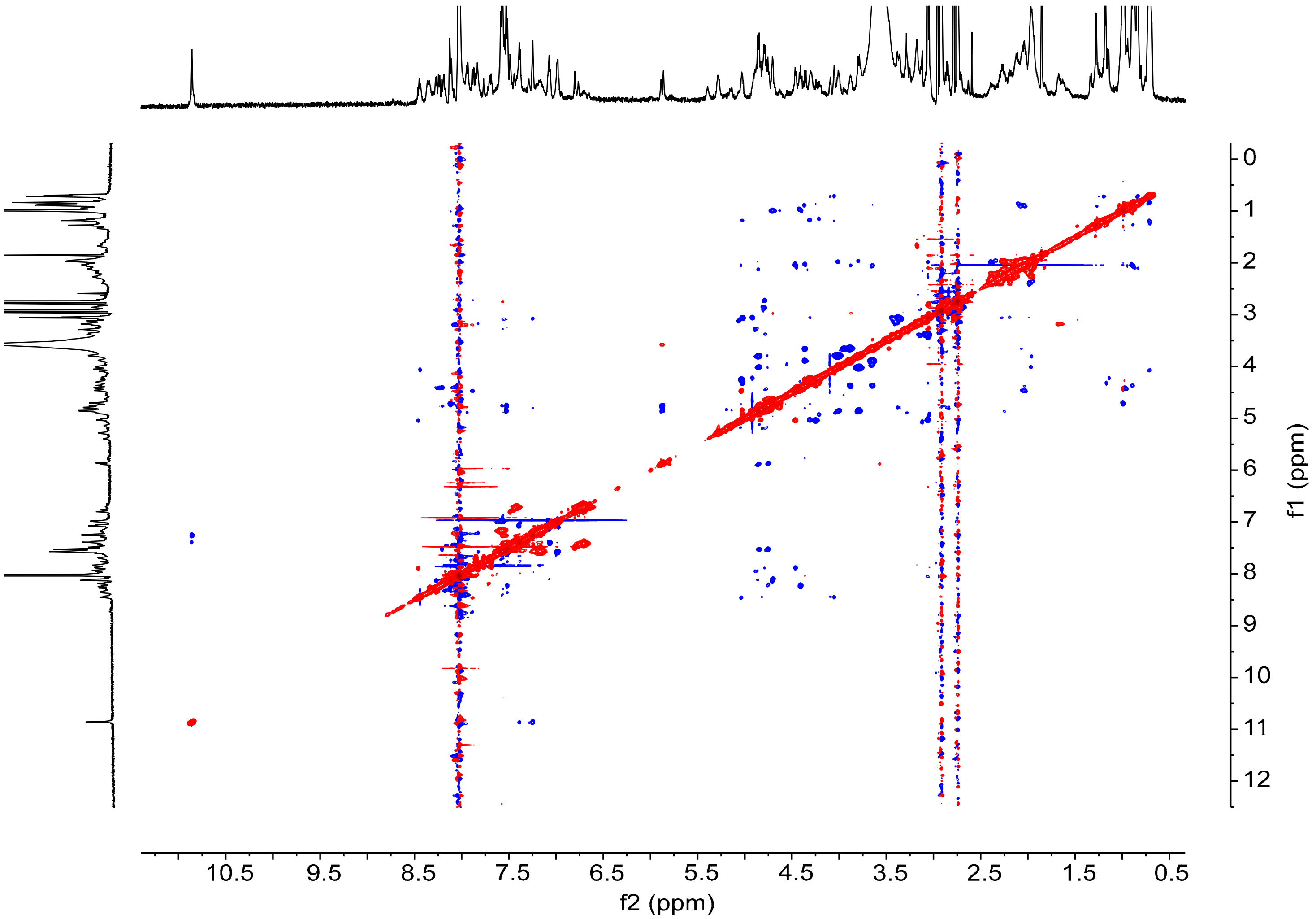

Figure S41. ^{1}H-^{1}H ROESY spectrum of **5** in DMF-d_7. The spectrum was acquired over 528 scans.

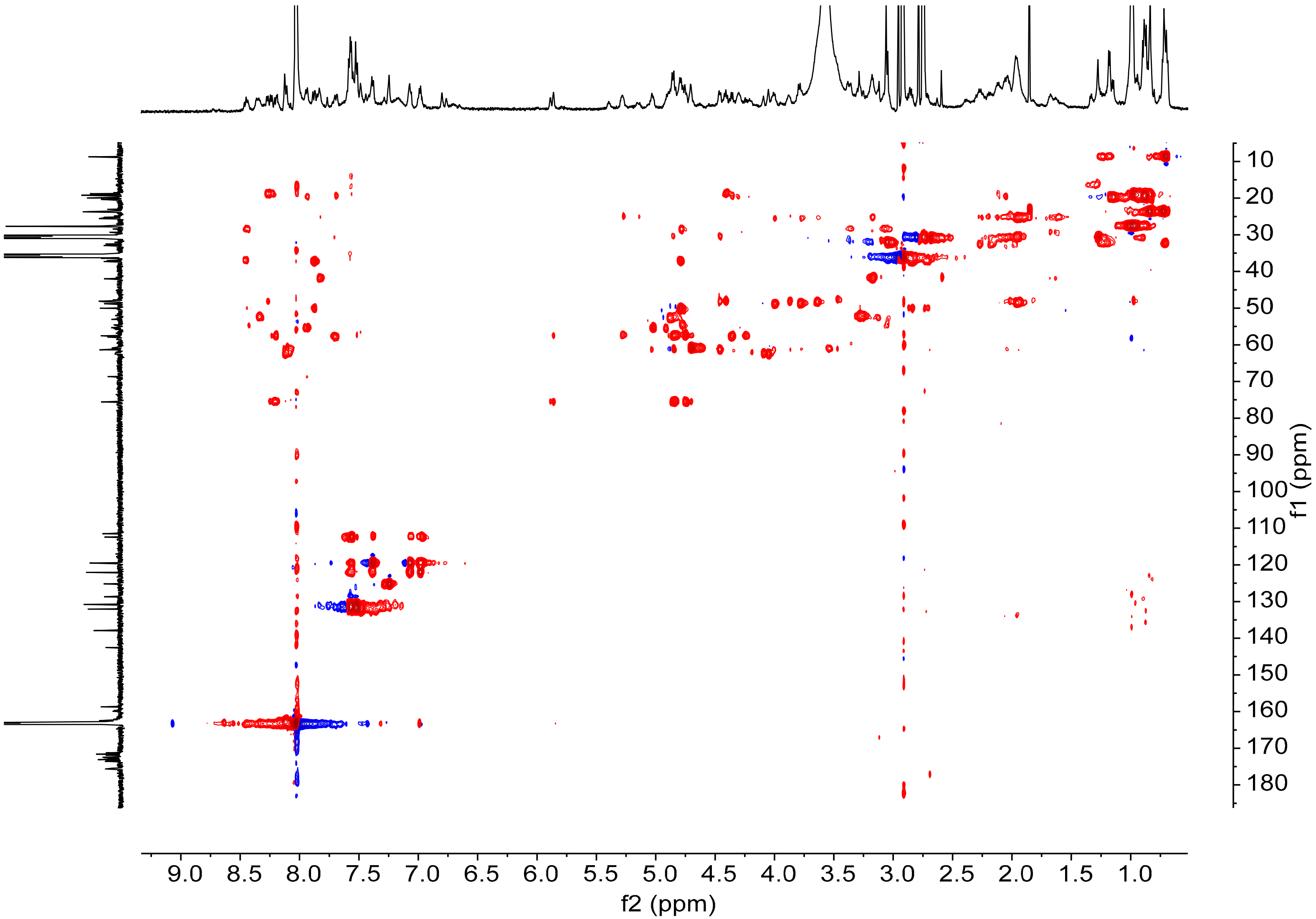

Figure S42. ^{1}H-^{13}C HSQC-TOCSY spectrum of **5** in DMF-d_7. The spectrum was acquired over 80 scans.

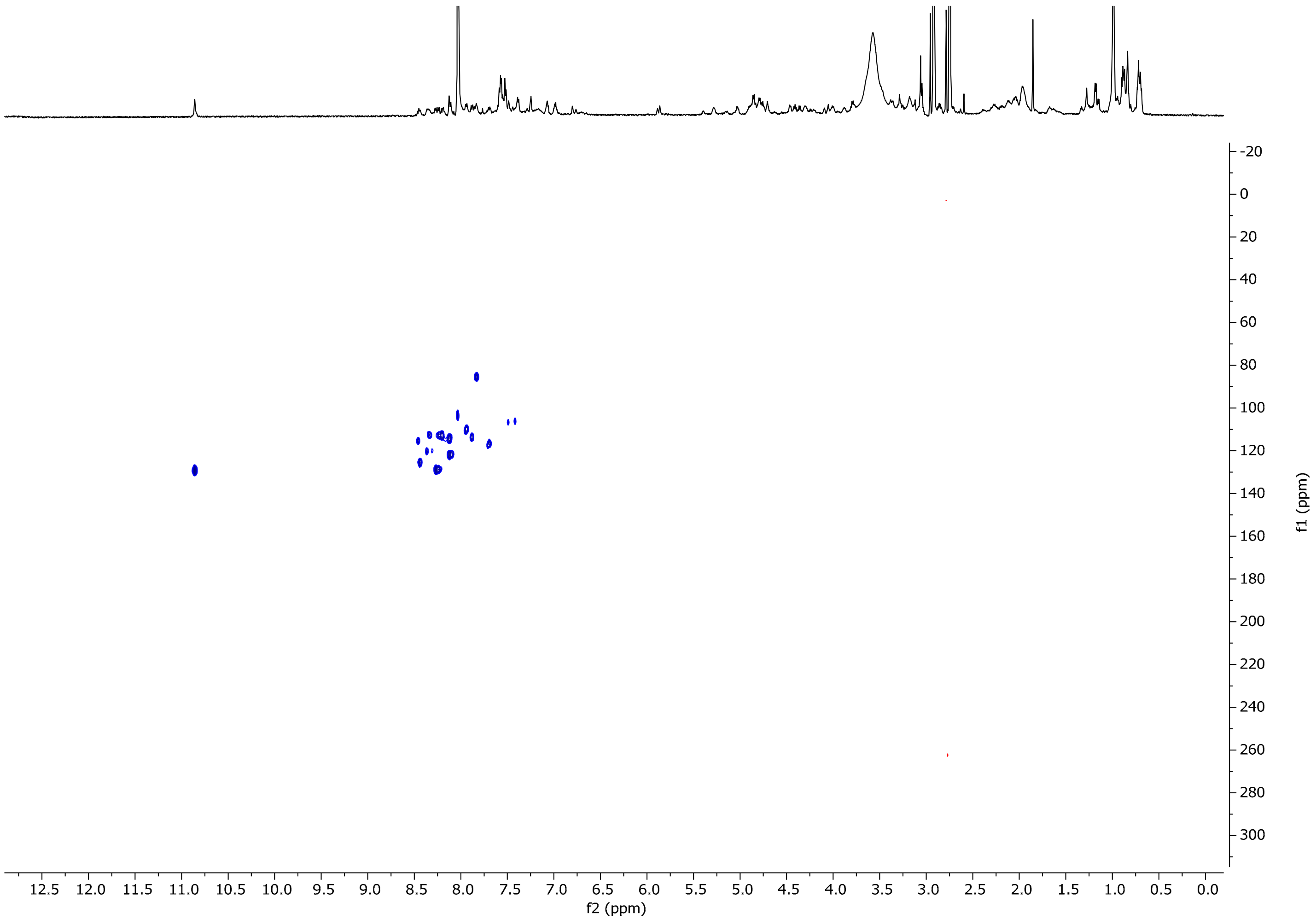

Figure S43. ^{1}H-^{15}N HSQC spectrum of **5** in DMF-d_7. The spectrum was acquired over 128 scans.

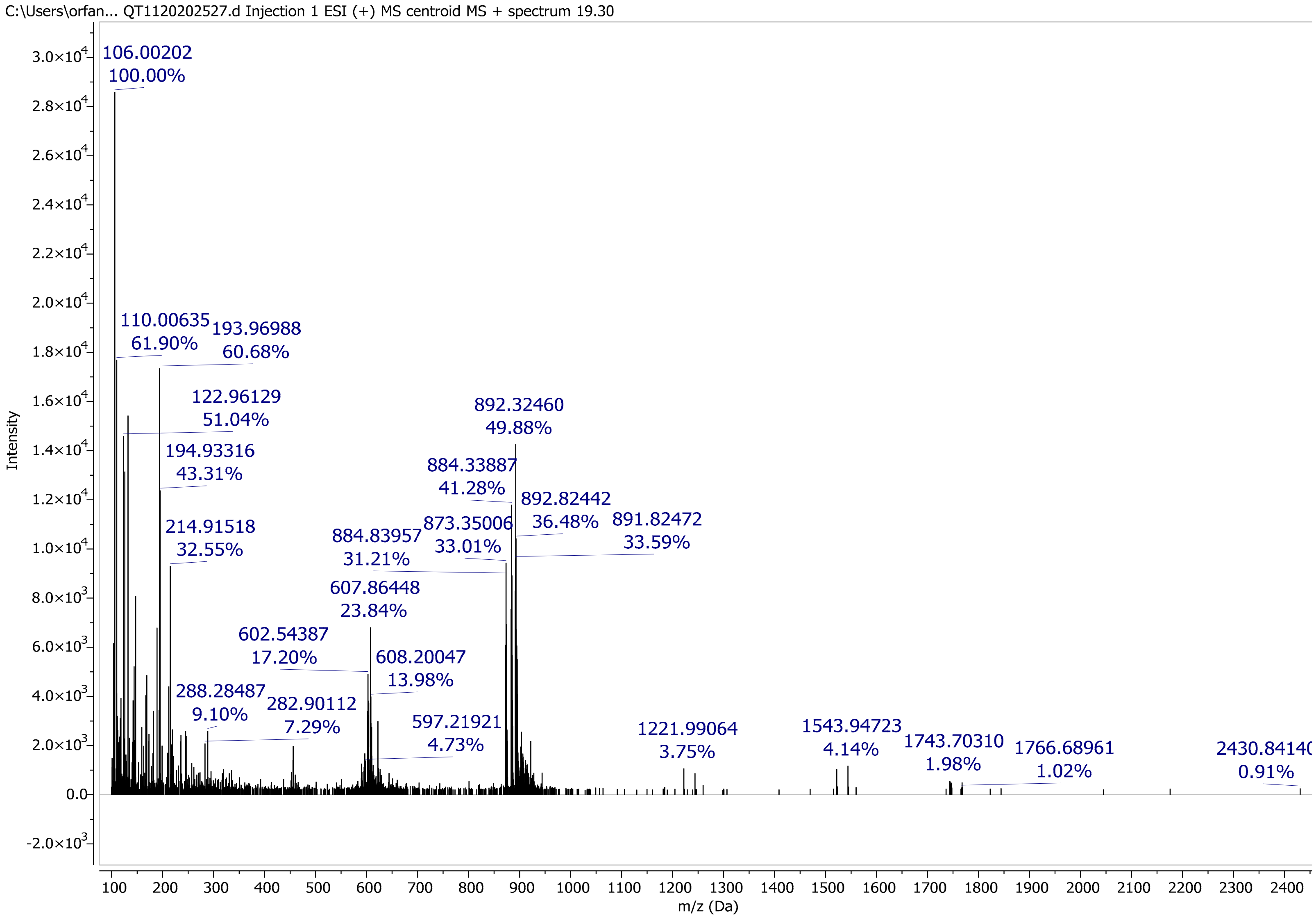

Figure S44. HRESIMS spectrum of **5**.

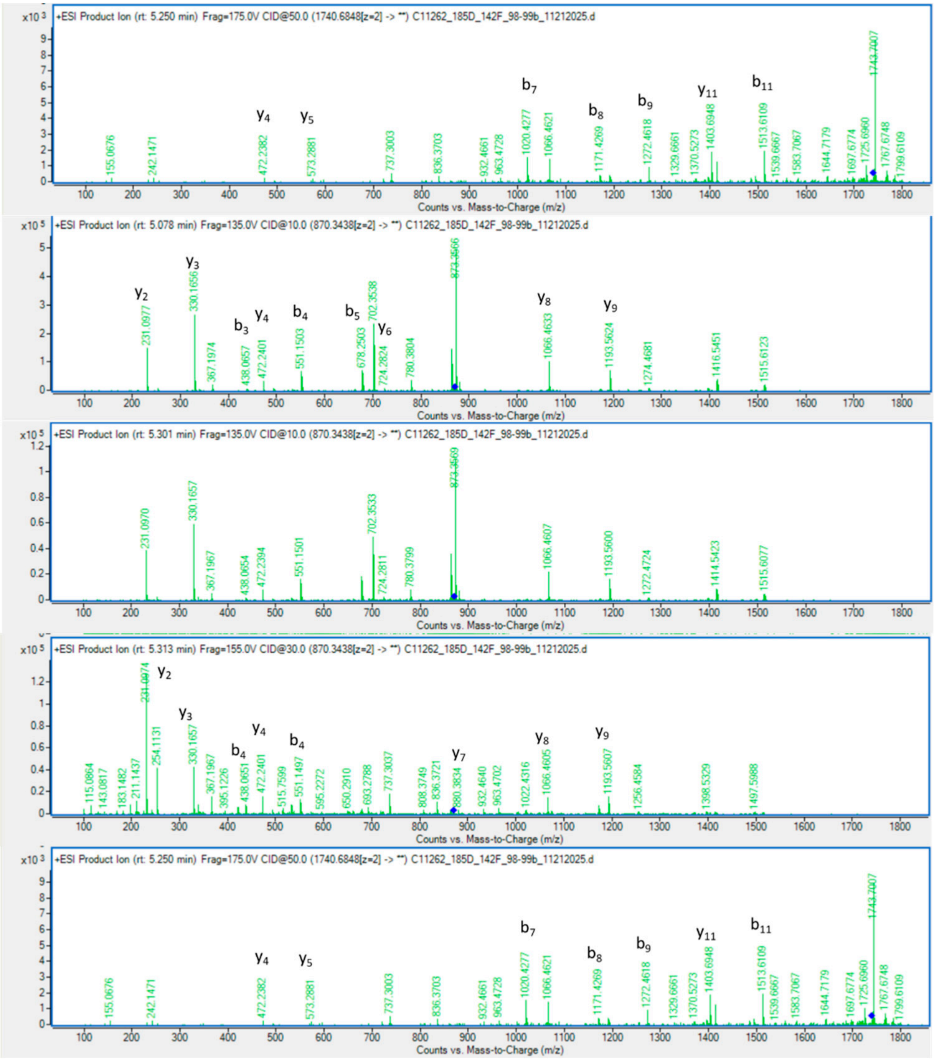

Figure S45. Positive HR-ESI-MS-MS b and y ion fragments of **5**.

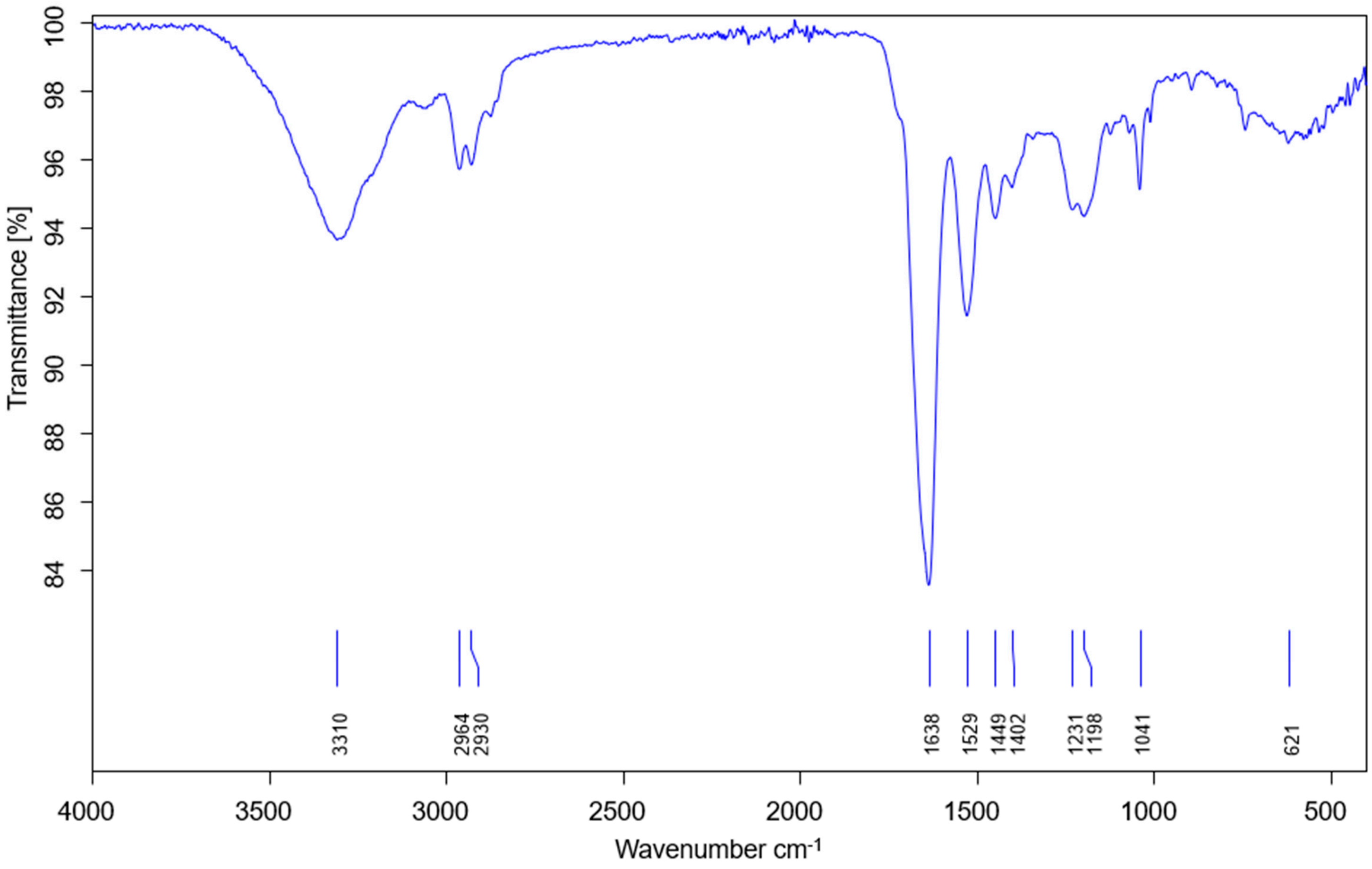

Figure S46. IR spectrum of **5**.

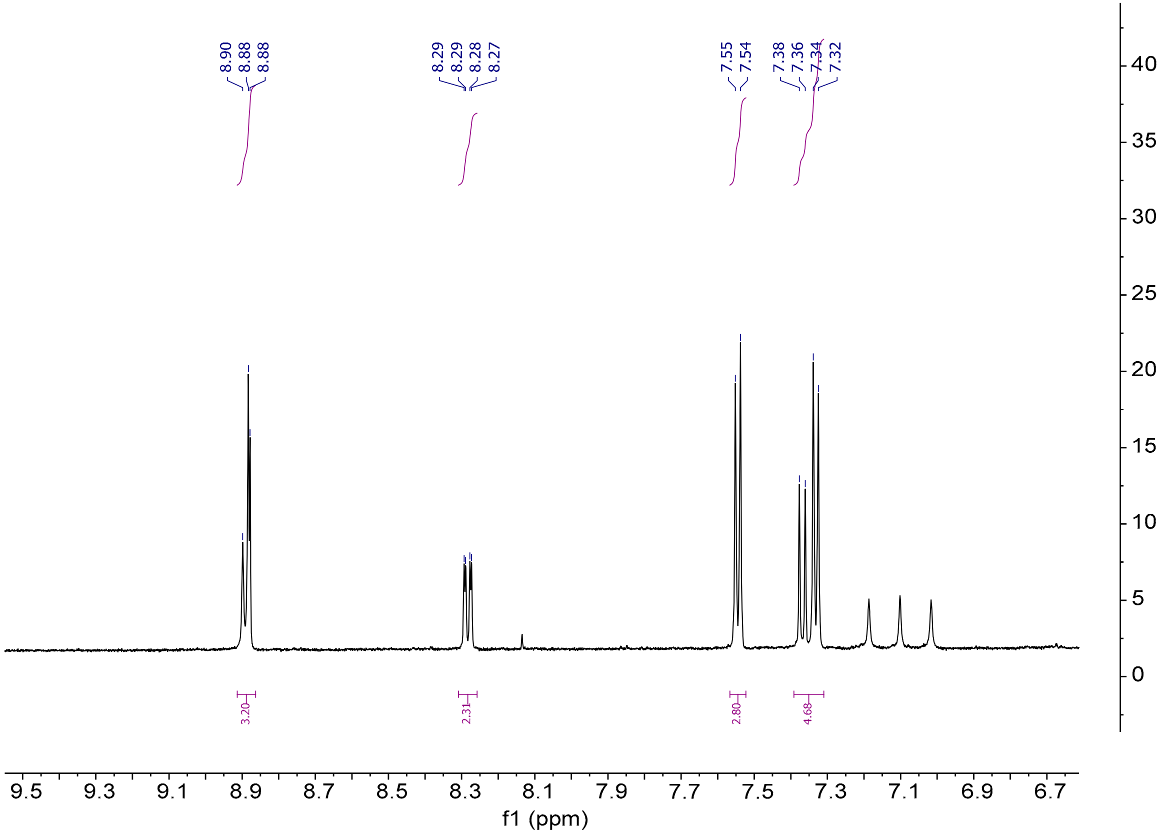

Figure S47. ^{1}H NMR spectrum (600 MHz, DMSO-d_6) of **6**. The spectrum was acquired over 4 scans.

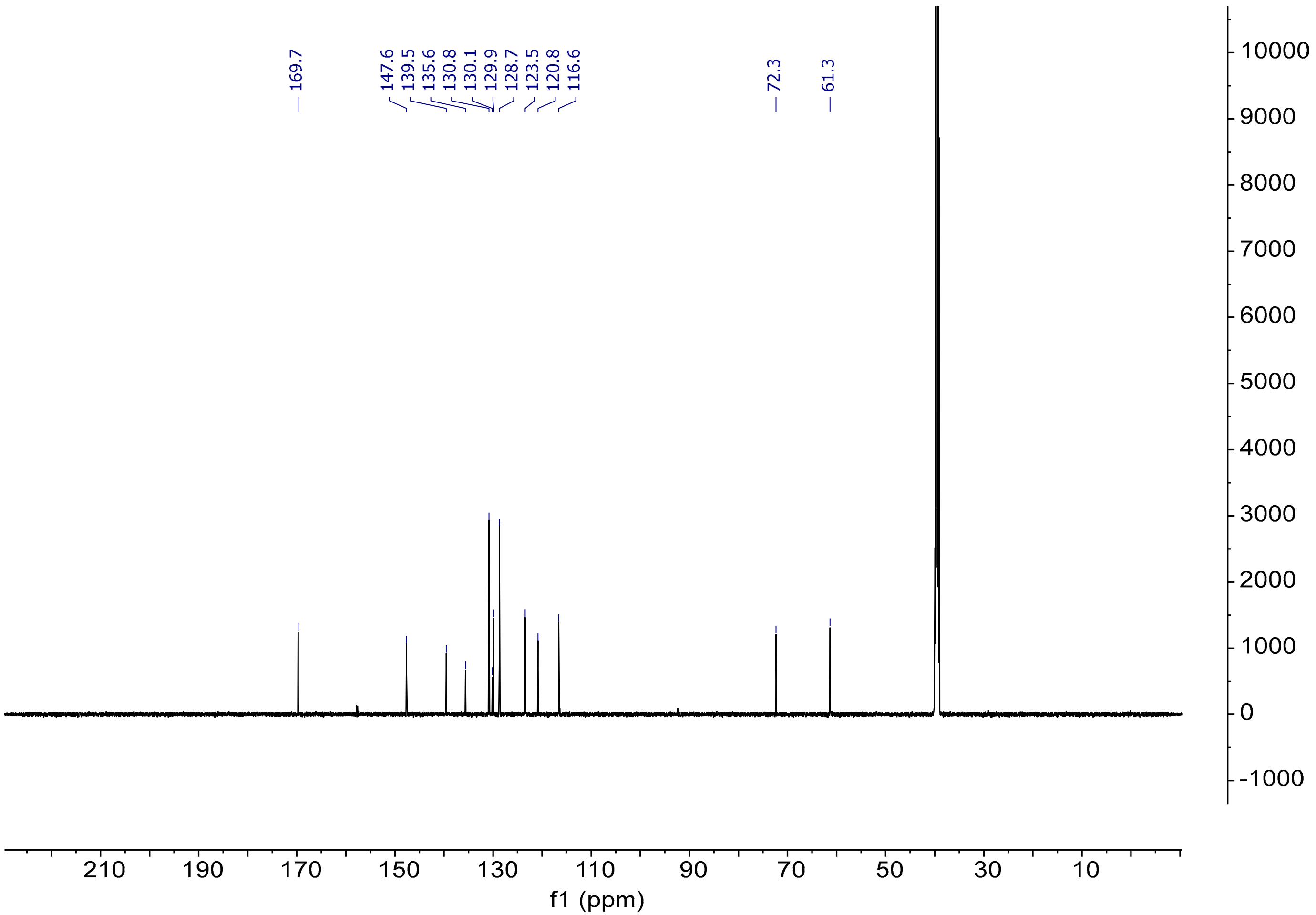

Figure S48. ^{13}C NMR spectrum (150 MHz, DMSO-d_6) of **6**. The spectrum was acquired over 15000 scans.

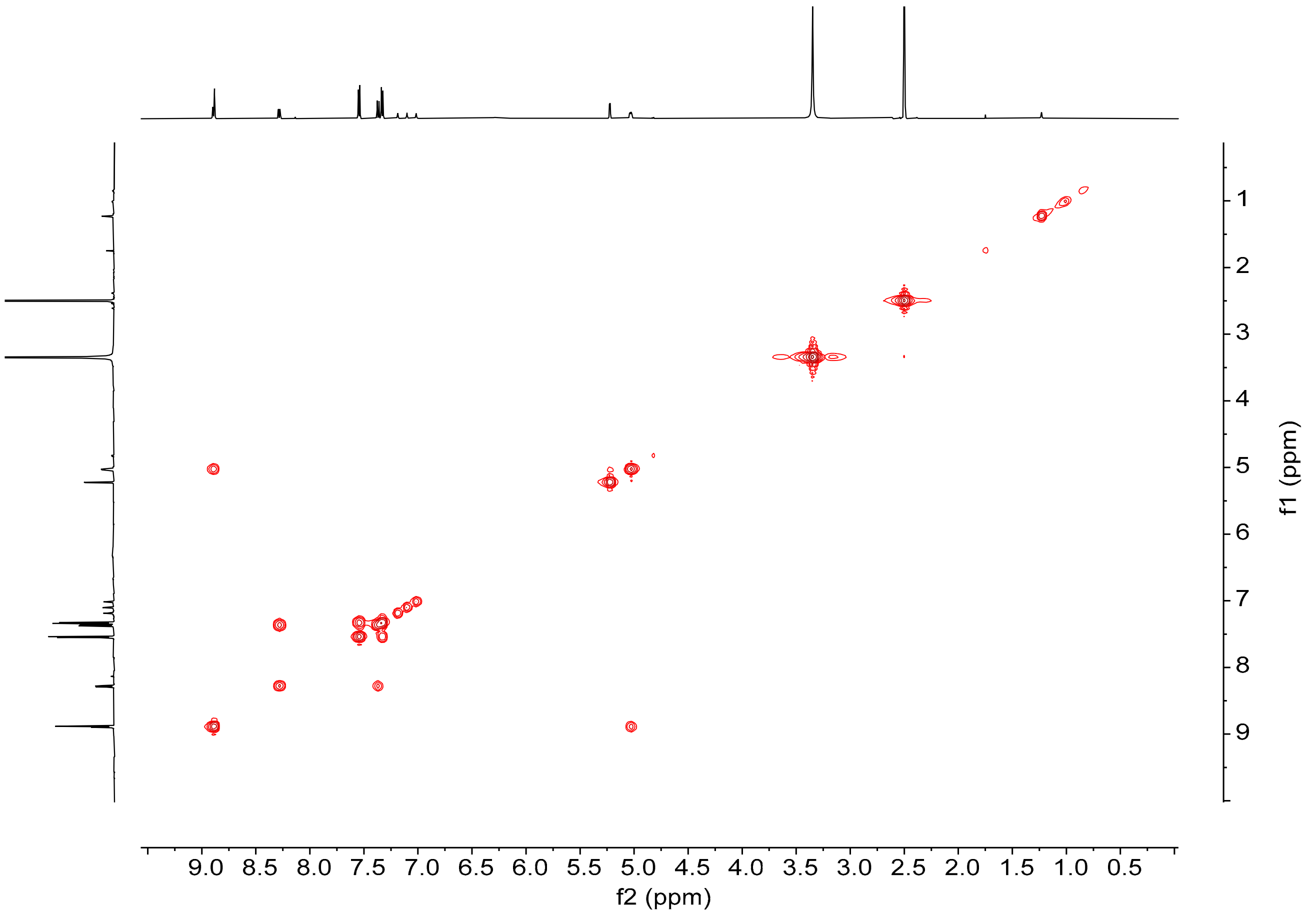

Figure S49. ^{1}H-^{1}H COSY spectrum of **6** in DMSO-d_6. The spectrum was acquired over 16 scans.

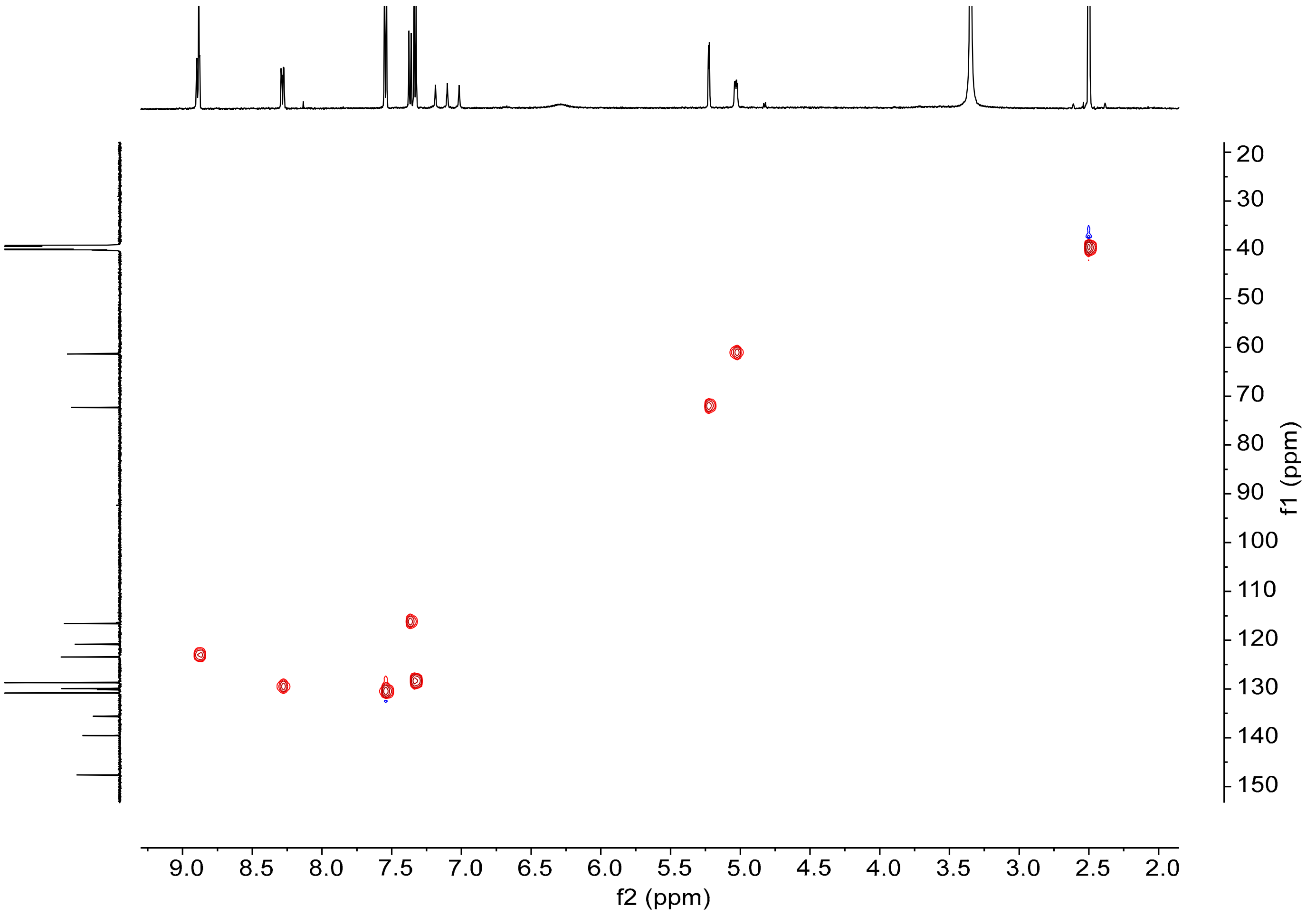

Figure S50. ^{1}H-^{13}C HSQC spectrum of **6** in DMSO-d_6. The spectrum was acquired over 32 scans.

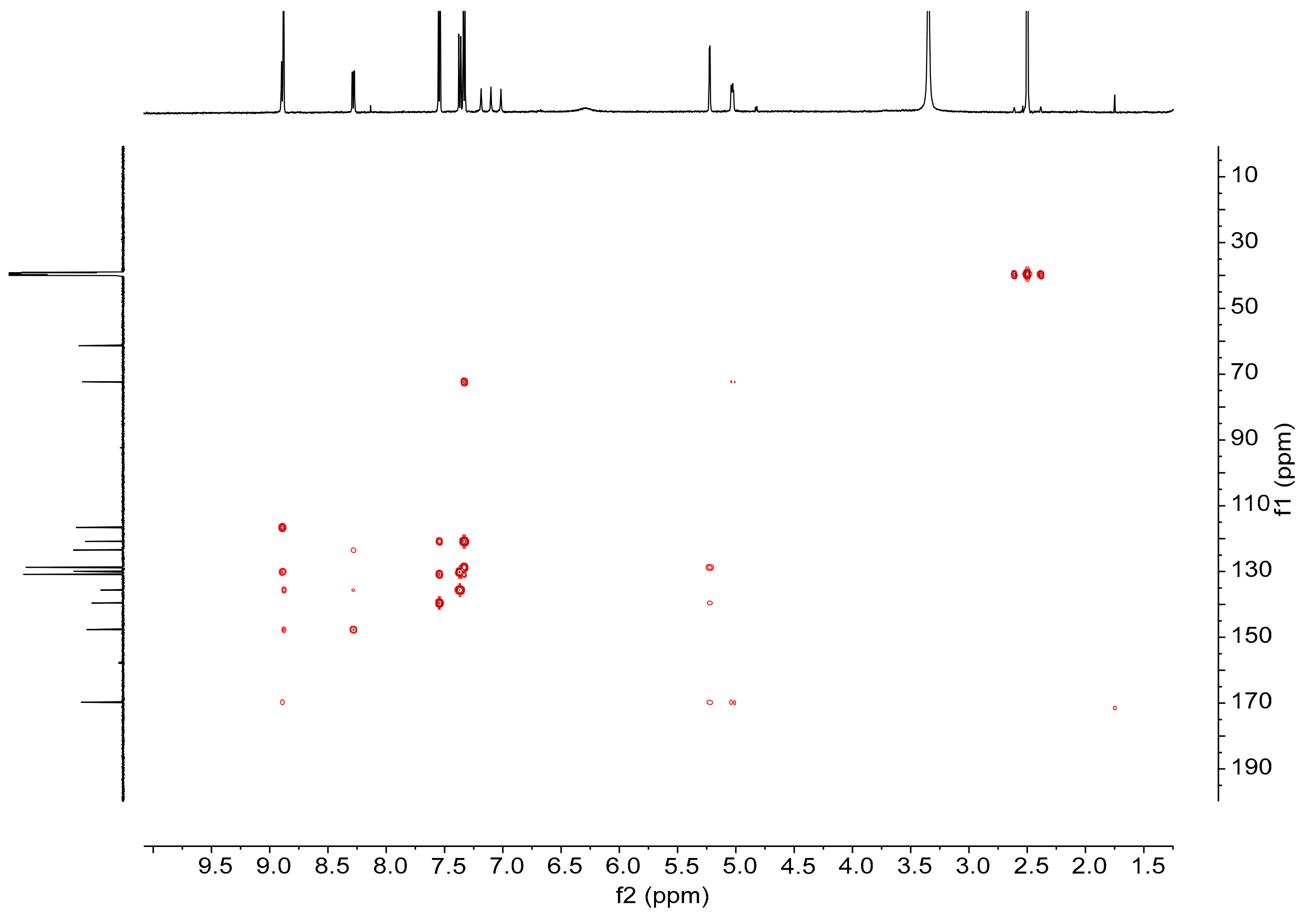

Figure S51. ^{1}H-^{13}C HMBC spectrum of **6** in DMSO-d_6. The spectrum was acquired over 420 scans.

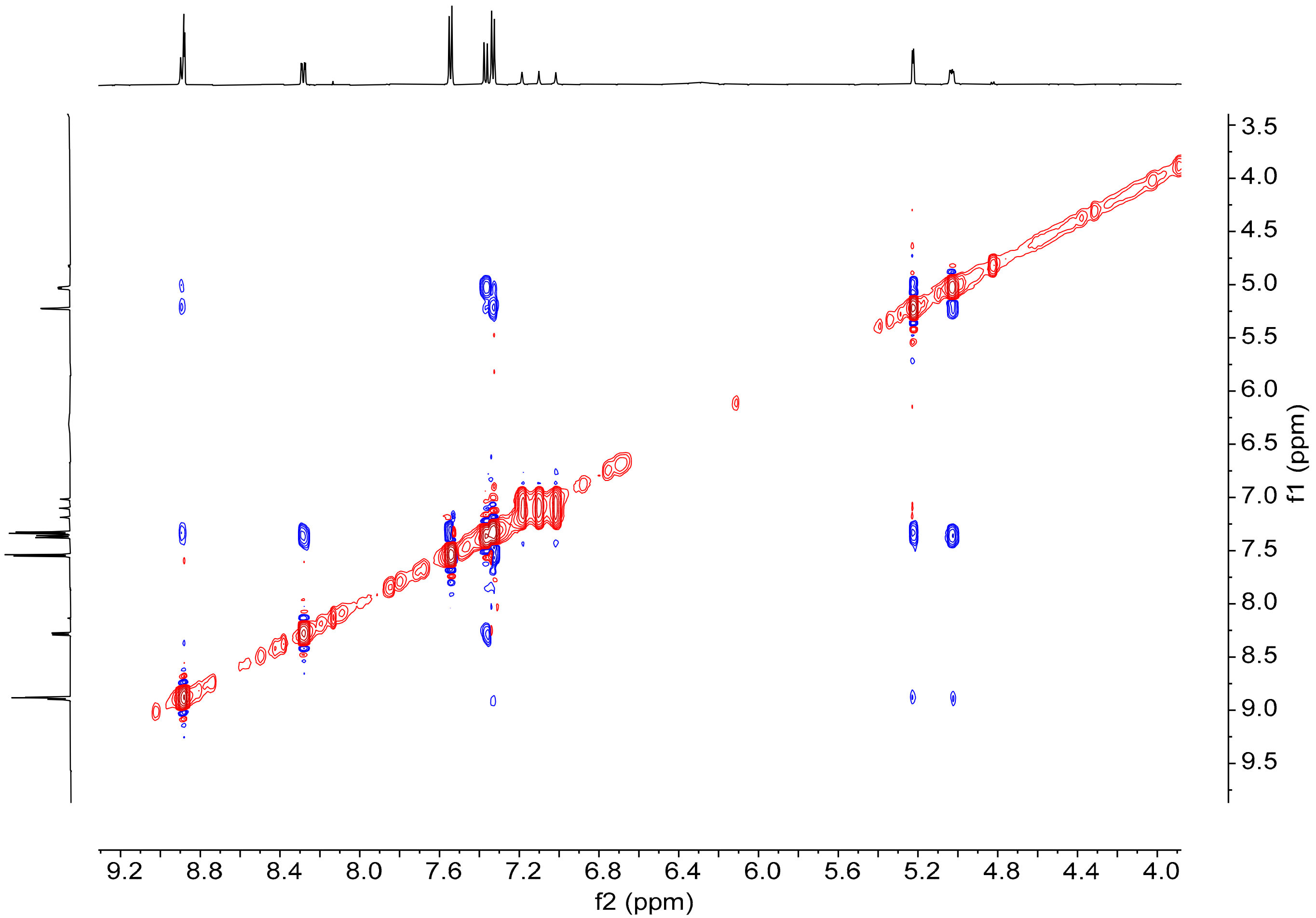

Figure S52. ^{1}H-^{1}H ROESY spectrum of **6** in DMSO-d_6. The spectrum was acquired over 160 scans.

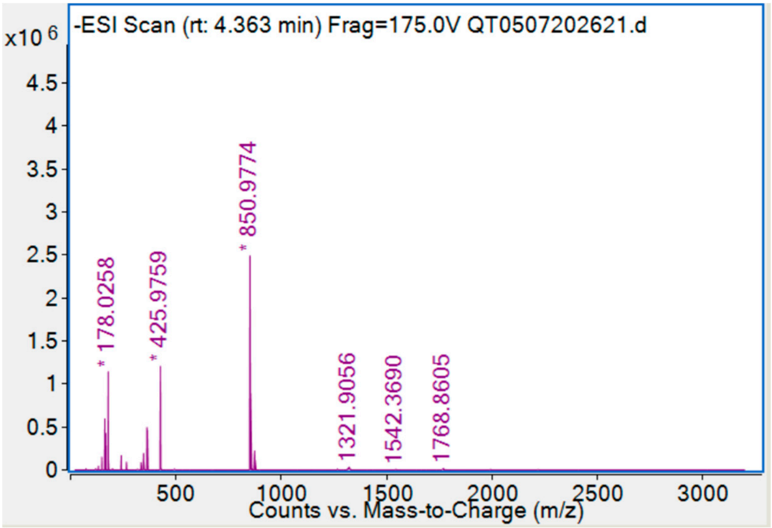

Figure S53. HRESIMS spectrum of **6**.

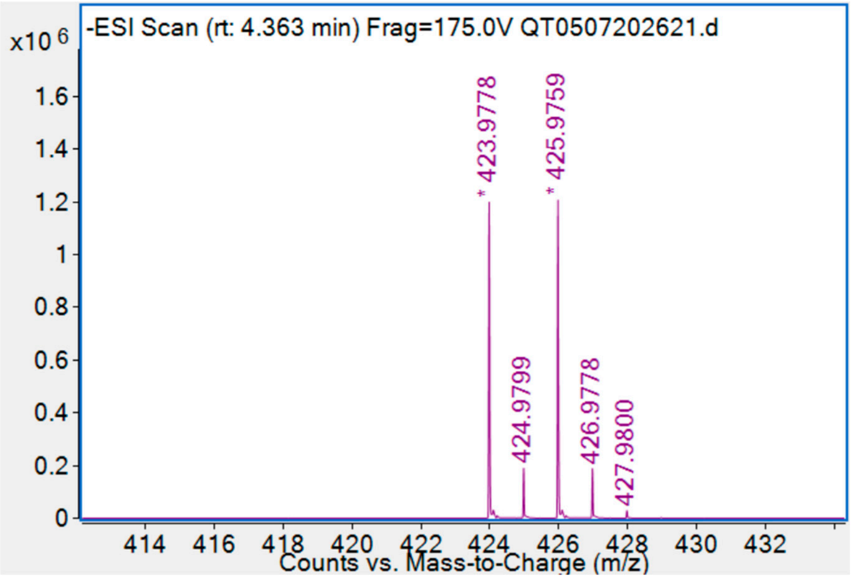

Figure S54. HRESIMS spectrum of **6** (zoomed).

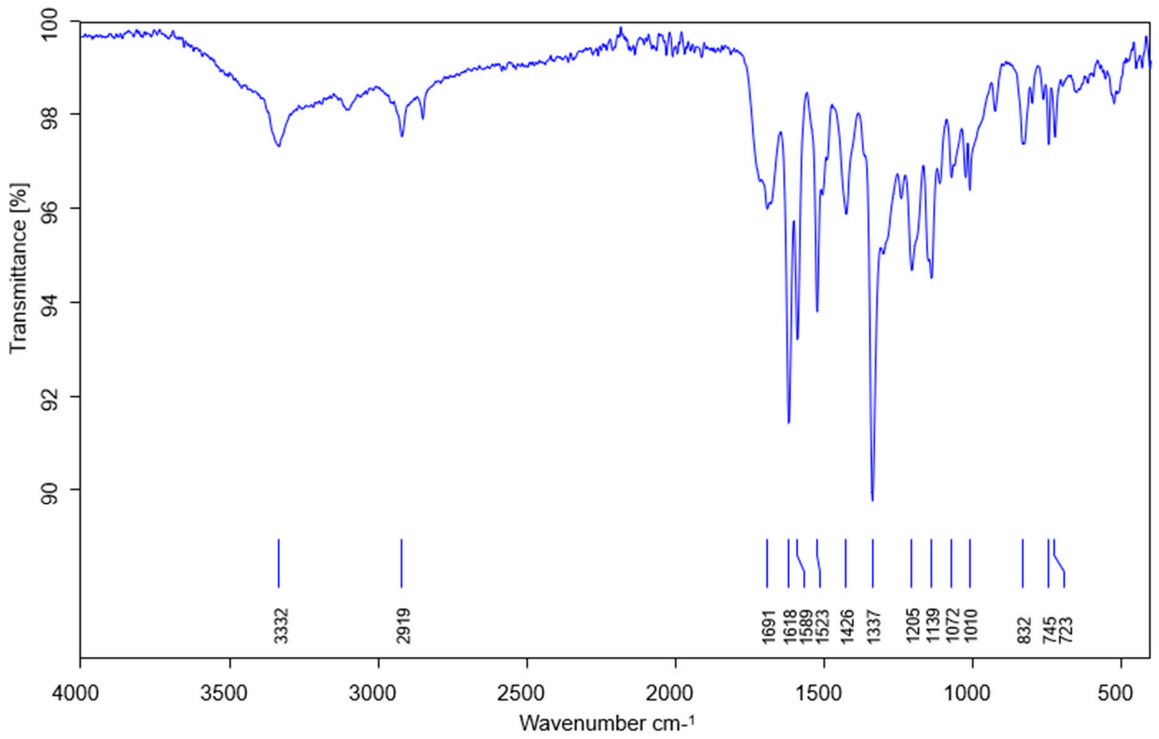

Figure S55. IR spectrum of **6**.
